# Genome-wide identification and expression profiling of SET DOMAIN GROUP family in *Dendrobium catenatum*

**DOI:** 10.1186/s12870-020-2244-6

**Published:** 2020-01-28

**Authors:** Dong-Hong Chen, Han-Lin Qiu, Yong Huang, Lei Zhang, Jin-Ping Si

**Affiliations:** 1 State Key Laboratory of Subtropical Silviculture, SFGA Engineering Research Center for Dendrobium catenatum (D. officinale), Zhejiang A&F University, Lin’an, Hangzhou, 311300 Zhejiang China; 2 Key Laboratory of Education Department of Hunan Province on Plant Genetics and Molecular Biology, Hunan Agricultural University, Changsha, 410128 China

**Keywords:** SDG, SET domain, Histone lysine methylation, Expression profiling, Environmental stress

## Abstract

**Background:**

*Dendrobium catenatum*, as a precious Chinese herbal medicine, is an epiphytic orchid plant, which grows on the trunks and cliffs and often faces up to diverse environmental stresses. SET DOMAIN GROUP (SDG) proteins act as histone lysine methyltransferases, which are involved in pleiotropic developmental events and stress responses through modifying chromatin structure and regulating gene transcription, but their roles in *D. catenatum* are unknown.

**Results:**

In this study, we identified 44 SDG proteins from *D. catenatum* genome. Subsequently, comprehensive analyses related to gene structure, protein domain organization, and phylogenetic relationship were performed to evaluate these *D. catenatum* SDG (DcSDG) proteins, along with the well-investigated homologs from the model plants *Arabidopsis thaliana* and *Oryza sativa* as well as the newly characterized 42 SDG proteins from a closely related orchid plant *Phalaenopsis equestris*. We showed DcSDG proteins can be grouped into eight distinct classes (I~VII and M), mostly consistent with the previous description. Based on the catalytic substrates of the reported SDG members mainly in Arabidopsis, Class I (E(z)-Like) is predicted to account for the deposition of H3K27me2/3, Class II (Ash-like) for H3K36me, Class III (Trx/ATX-like) for H3K4me2/3, Class M (ATXR3/7) for H3K4me, Class IV (Su (var)-like) for H3K27me1, Class V (Suv-like) for H3K9me, as well as class VI (S-ET) and class VII (RBCMT) for methylation of both histone and non-histone proteins. RNA-seq derived expression profiling showed that *DcSDG* proteins usually displayed wide but distinguished expressions in different tissues and organs. Finally, environmental stresses examination showed the expressions of *DcASHR3*, *DcSUVR3*, *DcATXR4*, *DcATXR5b*, and *DcSDG49* are closely associated with drought-recovery treatment, the expression of *DcSUVH5a*, *DcATXR5a* and *DcSUVR14a* are significantly influenced by low temperature, and even 61% *DcSDG* genes are in response to heat shock.

**Conclusions:**

This study systematically identifies and classifies SDG genes in orchid plant *D. catenatum*, indicates their functional divergence during the evolution, and discovers their broad roles in the developmental programs and stress responses. These results provide constructive clues for further functional investigation and epigenetic mechanism dissection of SET-containing proteins in orchids.

## Background

Spatiotemporal expression patterns of a set of genes associated with developmental and environmental stimuli are largely regulated by epigenetic modifications, which mainly consist of DNA methylation, non-coding RNAs, chromatin remodeling and histone modifications [1]. Histone modifications may occur post-translationally on various residues in histone tails and core regions. These modifications mainly include methylation, acylation, phosphorylation, ubiquitination, citrullination, hydroxylation, O-GlcNAcylation and ADP-ribosylation [2, 3], which constitute different combinations, that is, “histone code”, and act in chromatin-templated processes [4, 5]. Among these covalent modifications, histone methylation displays complicated features as it not only occurs on distinct residues (lysine and arginine) and positions, but also involves different numbers (1~3) of methyl groups [6]. Histone lysine modification is catalyzed by histone lysine methyltransferases (HKMTases) / SET DOMAIN GROUP (SDG) proteins which commonly possess an evolutionarily conserved SET domain as catalytic module [7], except for H3K79 methyltransferase DOT1, which lacks a SET domain [8]. SDG-catalyzed histone methylation at specific lysine residues can cause similar or opposite effects on gene expression, such as those of H3K4 and H3K36 associated with gene activation, whereas those of H3K9, H3K27, H4K20 associated with gene repression [9].

Based on the sequence homology and phylogenetic reconstruction, plant SDG proteins can be categorized into seven distinct classes: class I, E(z) homologs (H3K27me writer); class II, ASH1 homologs (H3K36me writer); class III, Trx homologs and related proteins (H3K4me writer); class IV, proteins with a SET domain and a PHD domain; class V, Su (var) homologs and relatives (H3K9me writer); class VI, proteins with an interrupted SET domain (S-ET); class VII, Ribulose-1,5-bisphosphate carboxylase/oxygenase (Rubisco) methyltransferase (RBCMT) and other SET related proteins for targeting non-histone proteins [10, 11]. *SDG* genes have been discovered in bacteria, viruses, and eukaryotes [12, 13]. The presence of *SDG* genes in bacteria was initially considered a consequence of horizontal gene transfer from eukaryotic hosts [14, 15]. However, investigation on more released genomes of prokaryotic organisms including not only pathogens and symbionts, but also free-living bacteria and archaea suggests that *SDG* genes have undergone independent evolution in prokaryotes, and this event is unrelated to the evolution of eukaryotic SDGs, on the other hand, an ancient horizontal gene transfer occurred between bacteria and archaea [13, 16].

SDG family has currently been systematically identified and classified in the genomes of Arabidopsis (49 members) [10, 17], *Brassica rapa* (49) [11], *Vitis vinifera* (33) [18], *Populus trichocarpa* (59) [19], *Zea mays* (43) [20], *Oryza sativa* (43) [21], *Solanum lycopersicum* (52) [22], *Citrus sinensis* (47) [23], *Gossypium raimondii* (52) [24], C4 panicoid model *Setaria italica* (53) [25], and *Litchi chinensis* (48) [26]. However, the SDG family in orchid species, which constitute an extremely evolutionary branch, remains elusive.

SDG proteins and related histone methylation marks are widely involved in diverse growth and developmental processes, such as seed dormancy, repression of vegetative-to-embryonic reversion, shoot branching, root system architecture, chloroplast development, flowering time, vernalization, floral organ development, ovule and anther development, embryo and endosperm development, plant senescence, carotenoid biosynthesis, and thigmomorphogenesis [27–32]. They are also implicated in the response to biotic and abiotic stresses. SDG8 is required for plant defense against necrotrophic fungal pathogens by regulating a subset of genes within jasmonic acid (JA) and/or ethylene signaling pathway [27] and for basal and R protein-mediated resistance to bacterial pathogens in Arabidopsis [33]. Loss-of-function mutant *sdg8* results in enhanced susceptibility to the fungal and bacterial pathogens. *ARABIDOPSIS* TRITHORAX-LIKE PROTEIN1 (ATX1) as H3K4me3 writer orchestrates expression of defense response genes in antagonistic salicylic acid (SA)/JA signaling pathways by directly activating the expression of the SA/JA signaling mediator *WRKY70* gene through establishing H3K4me3 marks on its nucleosomes [34]. In addition, ATX1 is involved in drought stress response, and its disruption results in decreased tolerance to dehydration stress in *atx1* plants [35]. ATX1 modulates dehydration stress signaling in both abscisic acid (ABA)-dependent and -independent pathways. During ABA-dependent pathway, dehydration stress induces ATX1 binding to *NCED3* locus, which encodes the rate-limiting enzyme in ABA biosynthesis. Subsequently the deposition of H3K4me3 mark and recruitment of RNA polymerase II are increased, leading to enhanced *NCED3* expression and ABA production [36]. Dehydration stress causes dynamic and specific changes in global histone H3K4me1/2/3 patterns in *Arabidopsis*, especially H3K4me3 marks with broad distribution profiles on the nucleosomes of stress-induced genes [37]. Similarly, drought stress triggers massive changes in H3K4me3 enrichments on numerous loci (respectively including 3927/910 genes with increased/decreased depositions) in rice seedlings, showing positive correlation with their transcript changes in response to drought stress [38]. However, when Arabidopsis exposed to cold temperatures, H3K27me3 deposition gradually decreases in the chromatins of two cold-responsive genes, *COR15A* and *ATGOLS3* [39].

*Dendrobium catenatum* (also known as *Dendrobium officinale*) belongs to the Orchidaceae family, is a rare and precious Chinese medicinal herb. The stem of *D. catenatum* is the major medicinal part used for relieving upset stomach, promoting body fluid production, and nourishing Yin and antipyresis in traditional remedy and health care [40]. Furthermore, the plant stem contains bioactive extracts with anticancer, hepatoprotective, hypolipidemic, antifatigue, antioxidant, anticonstipation, hypoglycemic, gastric ulcer-protective, and antihypertensive effects, and immunoenhancement, as confirmed by modern pharmacology [41]. However, given its long-time extensive demand and over-exploitation, *D. catenatum* suffers from near extinction, and was once defined as an endangered medicinal plant in China [42]. In the past 20 years, *D. catenatum* has been successfully cultivated and became an important economic crop for health care. Unfortunately, environmental stresses, such as drought, cold, and high temperature, extremely restrict its growth, resulting in heavy yield loss [43]. Hence, it is necessary to screen and identify the candidate genes conferring resistance to differential environmental stresses in *D. catenatum* molecular breeding.

To obtain a detailed understanding on the SDG family in medicinal orchid plants, we identified SDG members throughout the genome of *D. catenatum*, and subsequently performed comprehensive assessments on the phylogenetic relationship, gene structure, domain organization, gene expression profiling, and response to environmental stresses. Our results provide insights into the evolution and function of *SDG* genes in medicinal orchid plants.

## Results

### Identification of SDG proteins in the *D. catenatum* genome

**Fig. 1:**
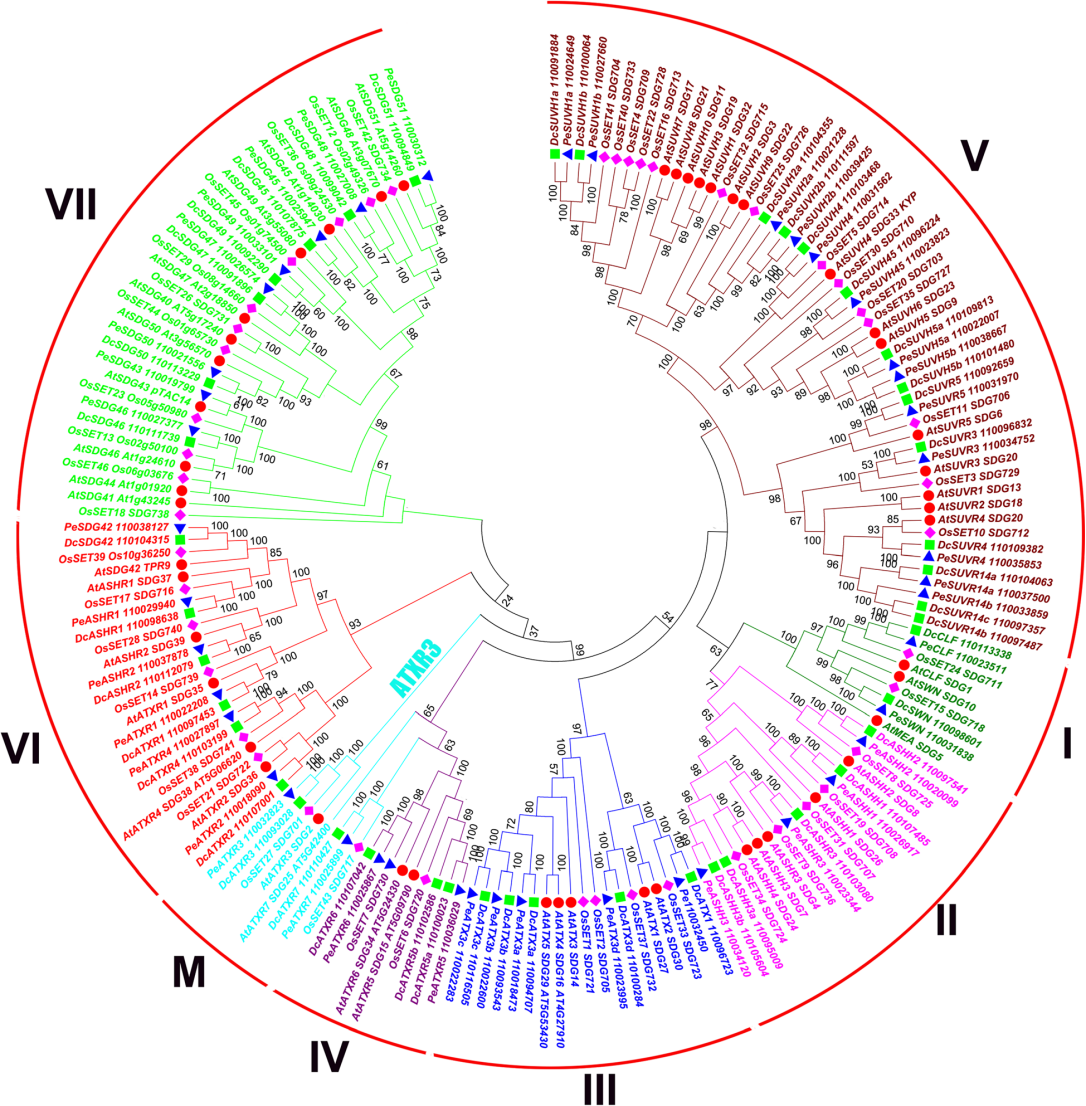
Phylogenetic analysis of SDGs in *D. catenatum*, *P. equestris*, Arabidopsis and rice. This tree includes 44 SET domain-containing proteins from *D. catenatum*, 42 from *P. equestris*, 49 from *A. thaliana* and 46 from *O. sativa.* The 181 SET domain-containing proteins were divided into eight classes combined with the further phylogenetic analysis in Fig. 4. SDGs amino acid sequences were aligned using Clustalx, and the phylogenetic tree was constructed using MEGA7 with the following settings: Tree Inference as Neighbor-Joining (NJ); Include Sites as Pairwise deletion option for total sequence analyses; Substitution Model: p-distance; and Bootstrap test of 1000 replicates for internal branch reliability. Bootstrap values > 50% are shown. Dc, *D. catenatum*; Pe, *P. equestris*; At, *A. thaliana*; Os, *O. sativa*

To obtain all the members of SDG proteins in *D. catenatum*, we performed BLASTP search using known Arabidopsis and rice SDG proteins as queries against the *D. catenatum* genome (INSDC: JSDN00000000.2). First, we checked the SDG genes of Arabidopsis and rice in the Superfamily 1.75 database (http://supfam.org/SUPERFAMILY/). We discovered 49 genes in *Arabidopsis thaliana*, corresponding to those reported in literature (Additional file 1) [10, 17]. On the other hand, 46 genes were identified in *Oryza sativa* (Additional file 1), three (Os01g65730/OsSET44, Os01g74500/OsSET45, Os06g03676/OsSET46) more than the 43 reported genes [21]. Reciprocal BLAST was carried out to confirm that the hits from *D. catenatum* and its close relative *Phalaenopsis equestris* belong to the SDG family. Finally, we obtained 44 SDG genes in *D. catenatum* (Table 1 and Fig. 1) and 42 in *P. equestris* (Additional file 2), and they were named after their Arabidopsis homologs.

**Table 1:** Classification of SDG genes in *D. catenatum*

No.	Name	Gene ID	Clade	exon number	Protein (aa)	Isoform
1	DcCLF	LOC110113338	I	16	918	2
2	DcSWN	LOC110098601	I	17	895	0
3	DcASHH1	LOC110107485	II	10	498	0
4	DcASHH2	LOC110097541	II	18	1992	0
5	DcASHH3a	LOC110095009	II	9	377	0
6	DcASHH3b	LOC110105604	II	9	235	0
7	DcASHR3	LOC110103080	II	11	487	1
8	DcATX1	LOC110096723	III	25	1081	0
9	DcATX3a	LOC110094707	III	22	980	0
10	DcATX3b	LOC110093543	III	24	1060	0
11	DcATX3c	LOC110116505	III	26	942	4
12	DcATX3d	LOC110100284	III	19	2038	0
13	DcATXR3	LOC110093028	M	21	2360	0
14	DcATXR7	LOC110110427	M	24	1304	6
15	DcATXR5a	LOC110100023	IV	7	413	3
16	DcATXR5b	LOC110102586	IV	8	373	3
17	DcATXR6	LOC110107042	IV	4	268	0
18	DcSUVH1a	LOC110091884	V	3	682	0
19	DcSUVH1b	LOC110100064	V	3	680	1
20	DcSUVH2a	LOC110104355	V	3	664	1
21	DcSUVH2b	LOC110111597	V	2	640	0
22	DcSUVH4	LOC110103468	V	20	752	4
23	DcSUVH45	LOC110096224	V	2	765	1
24	DcSUVH5a	LOC110109813	V	2	1048	1
25	DcSUVH5b	LOC110101480	V	2	1099	0
26	DcSUVR14a	LOC110104063	V	13	783	8
27	DcSUVR14b	LOC110097487	V	9	668	0
28	DcSUVR14c	LOC110097357	V	8	785	0
29	DcSUVR3	LOC110096832	V	2	337	0
30	DcSUVR4	LOC110109382	V	13	723	8
31	DcSUVR5	LOC110092659	V	14	1658	2
32	DcASHR1	LOC110098638	VI	14	496	0
33	DcASHR2	LOC110112079	VI	3	385	2
34	DcATXR1	LOC110097453	VI	1	521	0
35	DcATXR2	LOC110107001	VI	15	494	0
36	DcATXR4	LOC110103199	VI	8	339	0
37	DcSDG42	LOC110104315	VI	14	765	4
38	DcSDG45	LOC110107875	VII	6	490	0
39	DcSDG46	LOC110111739	VII	6	494	1
40	DcSDG47	LOC110091896	VII	11	556	2
41	DcSDG48	LOC110099042	VII	12	499	0
42	DcSDG49	LOC110092290	VII	16	476	5
43	DcSDG50	LOC110113229	VII	8	482	2
44	DcSDG51	LOC110094849	VII	13	503	0

To characterize and classify the SDG family in *D. catenatum*, we used SDG proteins in the dicot model plant *A. thaliana*, the monocot model plant *O. sativa*, and its close relative *P. equestris* as references for phylogenetic analysis. The results showed that the 181 SDG proteins from the above four species could be clustered into eight classes (I~VII and M), mostly corresponding to the classification criteria in Arabidopsis [10]. By contrast, ARABIDOPSIS TRITHORAX RELATED 3 (ATXR3) branch was previously classified into Class III [10], but it was separated from the other branches and near ATXR7-like proteins in Class IV in this study (Fig. 1). Considering their similar substrate specificities, we combined ATXR3 branch and the neighboring ATXR7-like proteins and categorized them under class M, as supported by the further phylogenetic analysis among Classes III, IV, and M (Fig. 4). Given the high limited reports on Class VI and VII, which feature potential functions for non-histone and histone methylation, we mainly focused on the roles of Classes I~V and M with well-investigated histone methylation specificity in this study.

### Class I: E(z)-like (H3K27me2/3)

**Fig. 2:**
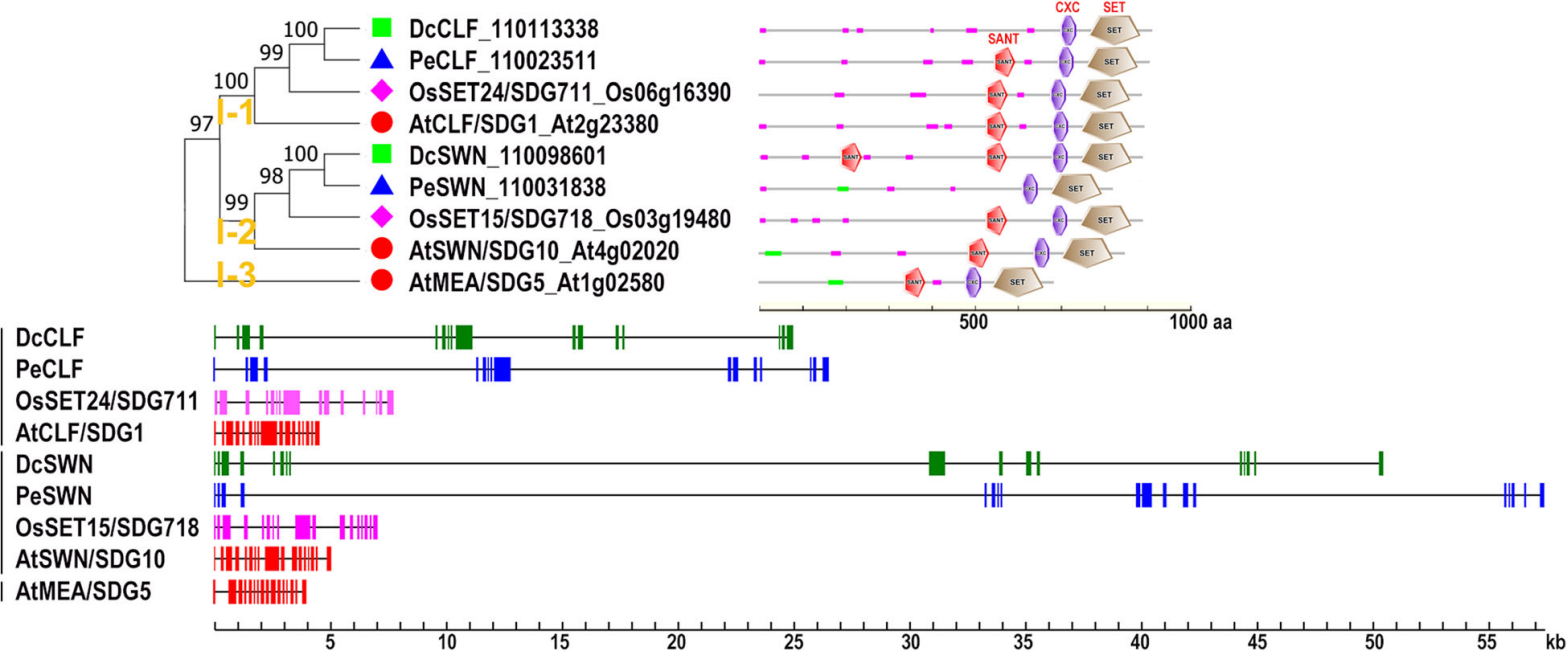
Domain organization and gene structure of the Class I DcSDGs. The NJ tree was generated using MEGA7 with parameter settings as Fig. 1 based on full-length amino acid sequences of Class I SDG proteins in *D. catenatum*, *P. equestris*, Arabidopsis and rice. The number along the tree branch indicates bootstrap value. Different conserved protein domains (SANT, CXC, and SET) are colored as indicated. Gene structures of SDGs in each species were indicated in distinct colors. The solid boxes represent exons and black lines represent introns

Class I contains two E(z) homologs in each of monocot plants *D. catenatum*, *P. equestris* and rice, and three well-characterized homologs, namely, CURLY LEAF (CLF), SWINGER (SWN), and MEAEA (MEA), that represent three distinct clades in the dicot plant Arabidopsis (Fig. 2). The genes in this class contain 15~16 introns, which are extremely longer in the two orchid species compared with those of Arabidopsis and rice. This result suggests that overall intron length positively correlated with the corresponding genome size. A similar phenomenon related to intron/exon proportion was also observed in the members of the other classes as will be mentioned later. The three Arabidopsis E(z) proteins act as the catalytic subunits of the evolutionarily conserved Polycomb Repressive Complex 2 (PRC2), which is involved in the deposition of H3K27me3 repressive mark on the target gene locus [44]. CLF (dominant H3K27me3 writer) and SWN act redundantly in vegetative and reproductive development, whereas MEA functions exclusively in suppression of central cell proliferation and endosperm development [45–47]. Rice E(z) homologs SDG711 and SDG718 participate in mediating accurate photoperiod control of flowering time [48]. Clades I-1 (CLF-like) and I-2 (SWN-like) each contain one ortholog in the examined species, but Clade I-3 (MEA-like) is confined to Arabidopsis. Plant E(z)-like proteins generally harbor highly conserved domain organization at the C-terminal region, which includes a SANT domain, the cysteine rich CXC domain, and the signature SET domain, except for DcCLF and PeSWN, which lack the SANT domain, and DcSWN, which possesses an additional SANT domain at the N-terminus.

### Class II: ash-like (H3K36me)

**Fig. 3:**
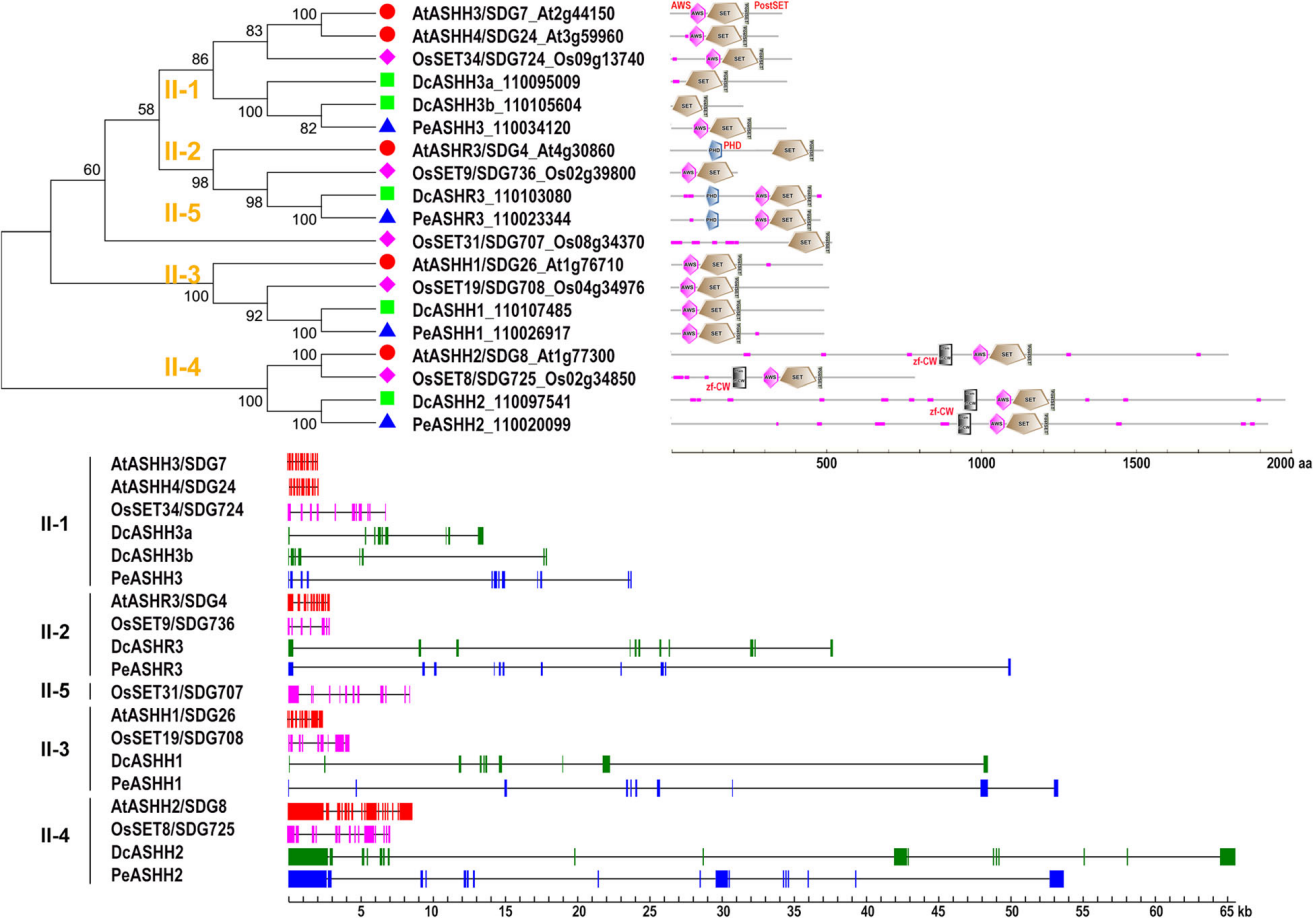
Domain organization and gene structure of the Class II SDGs. The NJ tree was generated using MEGA7 with parameter settings as Fig. 1 based on full-length amino acid sequences of Class II SDG proteins in *D. catenatum*, *P. equestris*, Arabidopsis and rice. The number along the tree branch indicates bootstrap value. Different conserved protein domains (AWS, PHD, zf-CW, SET and PostSET) are colored as indicated. Gene structures of SDGs in each species were indicated in distinct colors. The solid boxes represent exons and black lines represent introns

Class II can be further divided into five clades, each of which consists of a single member per plant species, except for two members of Clade II-1 in Arabidopsis and *D. catenatum* (Fig. 3). Clades II-1 to II-4 exist in all the examined species, but Clade II-5 is only found in rice and contains a single member, SDG707, with unknown function. Class II proteins generally share three conserved domains: an Associated with SET (AWS), SET, and PostSET domains [10, 49].

Clade II-1 (ASHH3-like) members are relatively shorter than their homologs in the four other clades. Arabidopsis ASHH3/SDG7 is required for proper timing in response to vernalization [50]. SDG7 lacks detectable HKMTase activity [51], but rice ortholog SDG724/LVP possesses H3K36 methylation activity. The loss of *SDG724* leads to late flowering [52]. Notably, DcASHH3a/3b in *D. catenatum* lack AWS domain, different from PeASHH3 from its close relative *P. equestris* and ASHH3 orthologs in Arabidopsis and rice, and their functional divergence during speciation is interesting to investigate.

Clade II-2 (ASHR3-like) members are characterized by an additional PHD domain near the N-terminus, except for rice SDG736. ASHR3/SDG4 participates in regulating pollen tube growth and stamen development, and its overexpression leads to growth arrest and male sterility [53, 54]. ASHR3 harbors catalytic activities on H3K36me1 and possible H3K36me2, which is involved in regulating cell division competence in the root meristem [55].

Clade II-3 (ASHH1-like) members display uniform protein length and highly conserved AWS-SET-PostSET domain combination at the N-terminus. Arabidopsis *ASHH1*/*SDG26* knockout leads to a late-flowering phenotype through decreasing H3K4me3 and H3K36me3 level at the *SOC1* locus [56, 57]. Similarly, the knockdown of rice ortholog *SDG708* causes a late-flowering phenotype and a genome-wide decrease in H3K36me1/2/3 levels during early growth stages [58]. Predictably, *D. catenatum* DcASHH1 harbors a similar function.

Clade II-4 (ASHH2-like) proteins are considerably longer than the others, and characterized by an additional CW domain near to the N-terminal triple domain combination. Arabidopsis ASHH2/SDG8 acts as the major H3K36me2/3 writer [57, 59], and its knockout leads to pleiotropic phenotypes in vegetative and reproductive stage [60]. Consistently, the knockdown of rice ortholog *SDG725* causes wide-ranging defects, including dwarfism, erect leaves and small seeds [32]. In the aspect of protein architecture, ASHH2 ortholog in *D. catenatum* or *P. equestris* is more like Arabidopsis SDG8 than rice SDG725.

### Class III: Trx/ATX-like (H3K4me2/3)

**Fig. 4:**
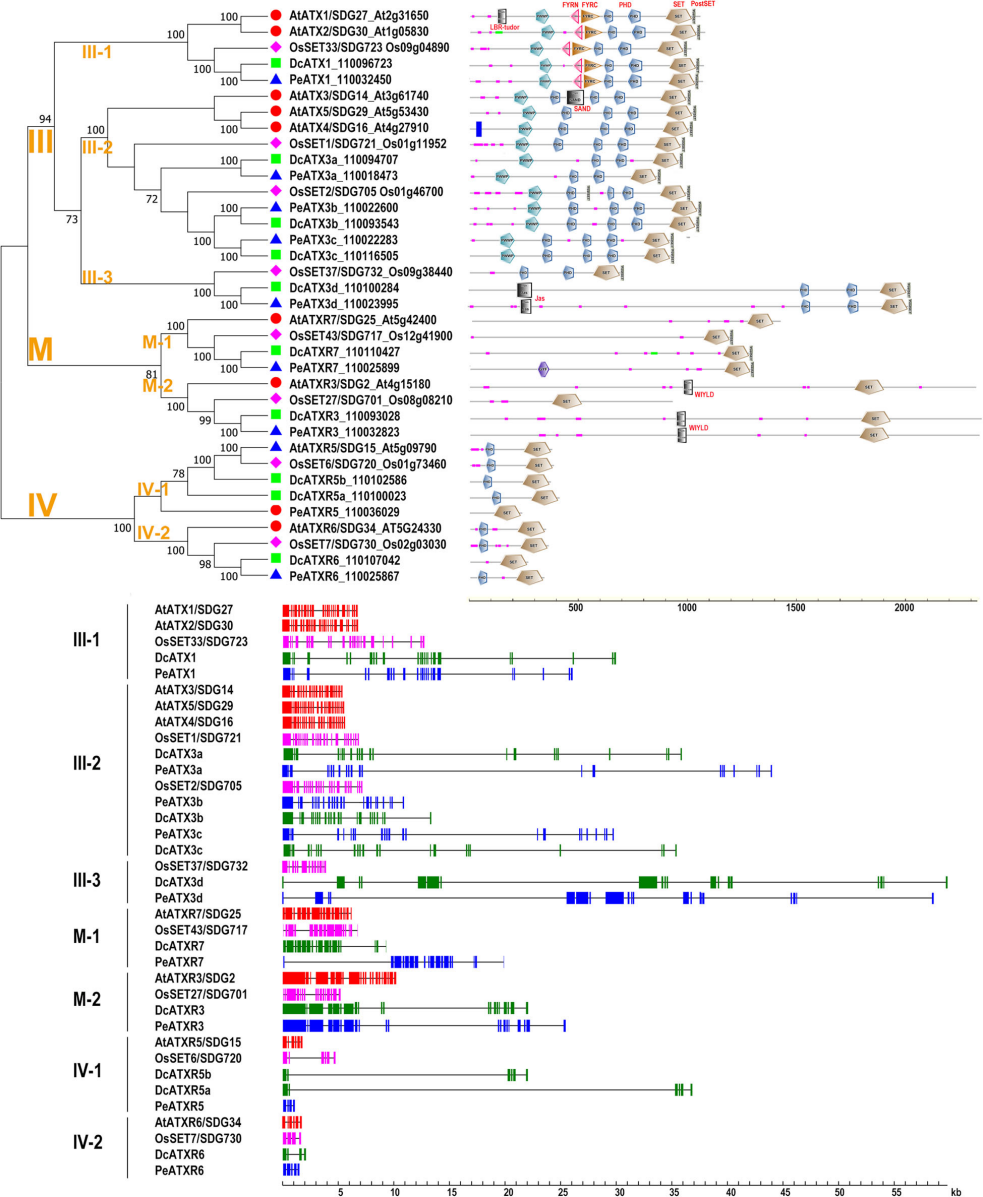
Domain organization and gene structure of the DcSDGs in Classes III, M and IV. The NJ tree was generated using MEGA7 with parameter settings as Fig. 1 based on full-length amino acid sequences of Class III/M/IV SDGs in *D. catenatum*, *P. equestris*, Arabidopsis and rice. The number along the tree branch indicates bootstrap value. Different conserved protein domains are colored as indicated. Gene structures of SDGs in each species were indicated in distinct colors. The solid boxes represent exons and black lines represent introns

Class III consists of five members, which can be further divided into three clades in each examined plant species (Fig. 4). Class III proteins are characterized by tandem PHD domains in the middle region and SET*–*PostSET domain combination at the N-terminus. Moreover, several clades contain additional distinct domains, such as PWWP domain specific to Clade III-1/2, and FYRN*–*FYRC domain combination specific to Clade III-1.

Clade III-1 (ATX1-like) contains two members in Arabidopsis, and one in each of the three other species. In Arabidopsis, ATX1 and ATX2 paralogs exhibit similar domain architectures [61], but have distinct expression patterns in most cases and influence the expressions of largely nonoverlapping gene sets [62]. For the shared targets, ATX1 and ATX2 account for the deposition of H3K4me3 and H3K4me2 marks, respectively [62]. Different from Arabidopsis *atx1* with early-flowering phenotype [62], rice ortholog mutant *ostrx1*/*sdg723* exhibits late-flowering through decreased H3K4me3 levels at the central flowering time integrator *Ehd1*. OsTRX1 can rescue Arabidopsis *atx1* phenotype [63–65], suggesting that ATX1-like proteins demonstrate conserved biochemical and molecular functions during evolution. However, ATX1-like proteins produce specific phenotypes in distinct species due to the differences in developmental context. Thus, DcATX1 and PeATX1 in orchid may play important roles in flowering time control.

Clade III-2 (ATX3-like) includes three members in each tested species. Arabidopsis ATX3/4/5 are clustered together and separated from the monocot orthologs. The orthologs from *D. catenatum*, and *P. equestris* are consistently clustered together, concordant with their close relationship. In Arabidopsis, ATX3/4/5 exhibit a common evolutionary origin, and function redundantly in genome-wide H3K4me2/3 profiles. Furthermore, *atx3 atx4 atx5* triple mutant displays dwarfism and reduced fertility [66]. In rice, ATX3-like proteins SDG721 and SDG705 function redundantly in modulating H3K4 methylation levels. The loss of both genes results in semi-dwarfism [67]. Considering the dwarf phenotype of ATX3-like mutants in Arabidopsis and rice, the homologs in *D. catenatum* and *P. equestris* might be involved in regulating plant architecture.

Clade III-3 shows specificity toward the examined monocots and contains one copy per species. *D. catenatum* DcATX3d and *P. equestris* PeATX3d are characterized by an additional Jas domain at the C-terminus, in contrast with the rice ortholog OsSET37/SDG732. Further survey of this clade will provide insights into the evolution of SDG family in monocots.

### Class M: ATXR3/7 (H3K4me)

Class M comprises of two clades, namely, Clade M-1 (ATXR7-like) and M-2 (ATXR3-like). Each clade contains one copy per plant species (Fig. 4). ATXR7-like proteins usually lack extra domains, except for PeATXR7 with a C-terminal GYF domain. Arabidopsis ATXR7/SDG25 acts as the writer of H3K4 monomethylation (H3K4me1), and its knockout results in early flowering [59, 68]. ATXR3-like proteins also contain only one copy in each species, are characterized by the presence of DUF4339 domain in the middle region, except for OsSET27/SDG701. Arabidopsis ATXR3/SDG2 is the major H3K4me3 writer, whose depletion leads to pleiotropic development defects [28, 69, 70]. *D. catenatum* DcATXR3 and *P. equestris* PeATXR3 feature a more similar protein architecture to Arabidopsis ATXR3/SDG2 than rice SDG701. This finding suggests that ATXR3-like proteins in orchid may retain their ancestral role, whereas rice ortholog may functionally diverge, as attributed to the loss of specific domain and partial sequence.

### Class IV: Su (var)-like (H3K27me1)

Class IV can be divided into two clades, Clade IV-I (ATXR5-like) and IV-II (ATXR6-like), which are characteristic of an N-terminal PHD domain in addition to the defined SET domain, except for PeATXR5 (Fig. 4). In Arabidopsis, ATXR5 and ATXR6 show largely overlapping functions, and the depletion of both results in global H3K27me1 reduction and heterochromatin decondensation [71, 72]. ATXR5/6 are involved in maintaining DNA replication [73] and repressing the expression of transposable element [74]. The overexpression of either ATXR5 or ATXR6 causes male sterility [75]. ATXR5 and ATXR6 probably perform separate roles because of ATXR5 with a dual localization in plastids and nucleus but ATXR6 solely in nucleus [75].

### Class V: Suv-like (H3K9me)

**Fig. 5:**
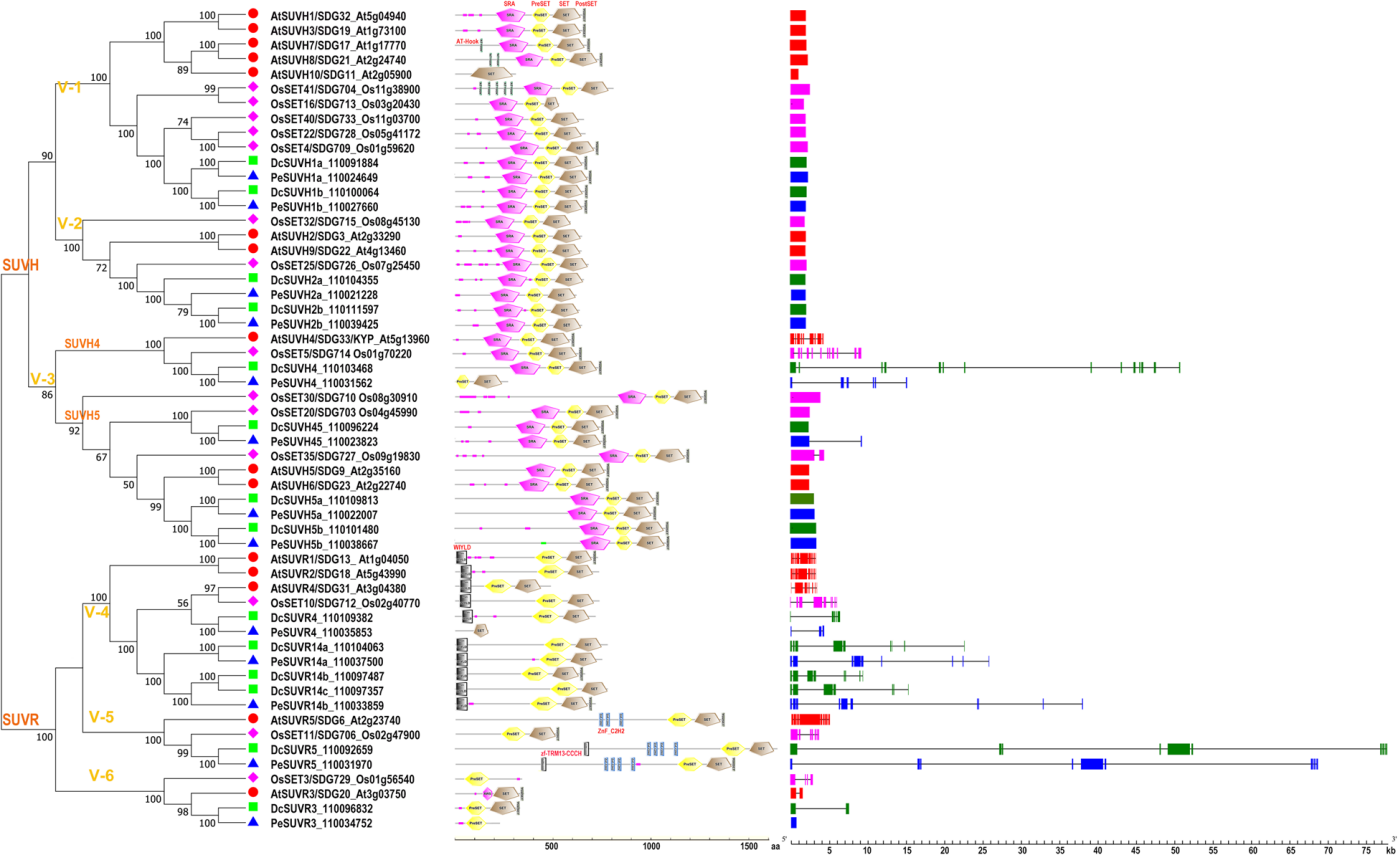
Domain organization and gene structure of the class V DcSDGs. The NJ tree was generated using MEGA7 with parameter settings as Fig. 1 based on full-length amino acid sequences of Class-V SDGs in *D. catenatum*, *P. equestris*, Arabidopsis and rice. The number along the tree branch indicates bootstrap value. Different conserved protein domains are colored as indicated. Gene structures of SDGs in each species were indicated in distinct colors. The solid boxes represent exons and black lines represent introns

Class V contains 15 members in Arabidopsis, 14 in rice and *D. catenatum*, and 13 in *P. equestris*; These members can be further divided into two subclasses, SUVH and SUVR, which include Clades V-1 to V-3 and V-4 to V-6, respectively (Fig. 5). Class V proteins are usually characterized by PreSET*–*SET*–*PostSET or PreSET*–*SET domain combinations. SUVH proteins often contain another symbolic SET- and RING-ASSOCIATED (SRA) domain, whereas SUVR proteins in Clades V-4 and V-5 often include another WIYLD domain and tandem ZnF_C2H2 domains, respectively. *SUVH* genes usually lack introns, except for the members of SUVH4 branch and two members (*PeSUVH45* and *SDG727*) of SUVH5 branch, whereas *SUVR* genes contain variable number of introns. In general, Class V members are responsible for methylation of histone H3 lysine 9 (H3K9me), in which H3K9 dimethylation (H3K9me2) is the critical mark for gene silencing and DNA methylation, and are involved in heterochromatin formation and reprogramming of gene expression [76].

### SUVH subclass

In Clade V-1, the five members SUVH1/3/7/8/10 in Arabidopsis cluster together and show distinction from the five homologs in rice and each of the two homologs in *D. catenatum* or *P. equestris*. This result indicates that duplication of these clade members occurred after divergence between dicots and monocots. However, the two orthologs in either *D. catenatum* or *P. equestris* respectively pair together, indicating that their gene duplication occurred before the split of *Dendrobium* and *Phalaenopsis*. Arabidopsis SUVH1/SDG32 performs a distinct anti-silencing function to promote the expression of DNA methylation-targeted genes. *SUVH1* knockout causes no effect on H3K9me2 levels but reduces H3K4me3 levels [77]. Furthermore, SUVH1 binds to highly methylated genomic loci targeted by RNA-directed DNA methylation (RdDM). However, rice SUVH1-like protein SDG728 retains its classical function to mediate H3K9 methylation and participates in retrotransposon repression [78]. *D. catenatum* and *P. equestris* include two SUVH1-like proteins, far less than the five members in Arabidopsis and rice. Thus, the function of SUVH1 homologs and the evolutionary mechanisms in orchids require further investigation.

Clade V-2 comprises two members for each examined species, and these members lack PostSET domains, in contrast with those in clade V-1. In Arabidopsis, SUVH2 and SUVH9 as sister paralogs show overlapping functions in RdDM and heterochromatic gene silencing [79, 80]. SUVH2 overexpression leads to ectopic heterochromatization accompanied with significant developmental defects, such as extreme dwarfism [79, 81]. SUVH2 and SUVH9 may feature inactive histone methyltransferase activity [82, 83]. However, the simultaneous absences of *SUVH2* and *SUVH9* lead to a marked decrease in H3K9me2 levels in the RdDM loci [80, 84]. SUVH2 and SUVH9 can bind to methylated DNA and facilitate the recruitment of Pol V to RdDM loci [82, 84]. Considering the highly similar domain organization among SUVH2-like proteins in these examined species, their function should be evolutionarily conserved.

Clade V-3 proteins could be further divided into two branches (SUVH4 and SUVH5). SUVH4 branch possesses one member in each species, whereas SUVH5 branch contains two members in Arabidopsis and rice, and three members in *D. catenatum* or *P. equestris*. In Arabidopsis, SUVH4/KRYPTONITE (KYP), SUVH5, and SUVH6 as H3K9 methyltransferases, are required to maintain DNA methylation [85–90]. SUVH4 as the predominant H3K9me1/2 writer [76]. SUVH5 and SUVH6 as sister paralogs in SUVH5 branch exhibit HKMTase activities with locus-specific features [76, 86, 87]. In rice, SUVH4-like protein SDG714 mediates H3K9 methylation, participating in DNA methylation, transposition of transposable elements, and genome stability [91]. Notably, PeSUVH4 protein in *P. equestris* is evidently short and lack SRA and PostSET domains, compared with SUVH4-like proteins in the three other species. The divergence of SUVH4 between orchid genus *Dendrobium* and *phalaenopsis* is worthy of investigation.

### SUVR subclass

In Clade V-4, there are 3 members in Arabidopsis and *P. equestris*, 1 in rice, and 4 in *D. catenatum*, respectively. In Arabidopsis, SUVR2 mediates transcriptional silencing in both RdDM-dependent and -independent manners [92]. SUVR4 participates in the epigenetic defense mechanism by introducing H3K9me3 marks to repress potentially harmful transposon activity [93]. SUVR4 specifically converts H3K9me1 into H3K9me3 at transposons and pseudogenes within the euchromatin [93, 94], but SUVR1 and SUVR2 show no detected histone methyltransferase activity in vitro [92, 95]. In this study, *D. catenatum* DcSUVR4, *P. equestris* PeSUVR4, and rice SDG712 were grouped together with Arabidopsis SUVR4 but not with SUVR1/2, implying that they possess ubiquitin-binding and HKMTase activities, except for PeSUVR4, which includes an obviously short sequence and lacks WIYLD and PreSET domains.

Clade V-5 contains one member in each tested species, and characterized by an additional tandem ZnF_C2H2 domain, except for SDG706. In Arabidopsis, SUVR5 lacks the SRA domain but recognizes specific DNA sequences through its zinc finger motifs and establishes the heterochromatic state through H3K9me2 deposition in a DNA methylation-independent manner [96]. The knockout of SUVR5 leads to delayed flowering, and no further enhanced phenotype occurs in the quintuple *suvr1 suvr2 suvr3 suvr4 suvr5* mutants [96, 97]. This finding suggests that SUVR5 is a dominant developmental regulator in SUVR subclass.

Clade V-6 members exist in one copy in each species, and their encoding proteins are notably shorter than those of the other clades in this class. Arabidopsis SUVR3 contains an additional AWS domain close to the SET–PostSET domain combination, and DcSUVR3 contains an intact PreSET–SET–PostSET domain combination. However, SUVR3 orthologs in rice and *P. equestris* only contain a PreSET domain, suggesting that the genes in Clade V-6 may undergo less selective pressures and become increasingly divergent during evolution. The functions of the genes in this clade remain uncharacterized thus far.

### Tissue and organ expression profiles of *DcSDG* genes

To investigate the potential roles of *DcSDGs* during growth and development in *D. catenatum*, we detected the expression profiles of *DcSDGs* by reanalyzing the RNA-seq data from different plant tissues and organs, including leaf, root, green root tip, white part of root, stem, flower bud, sepal, labellum (lip), pollinia, and gynostemium (column) [98].

**Fig. 6:**
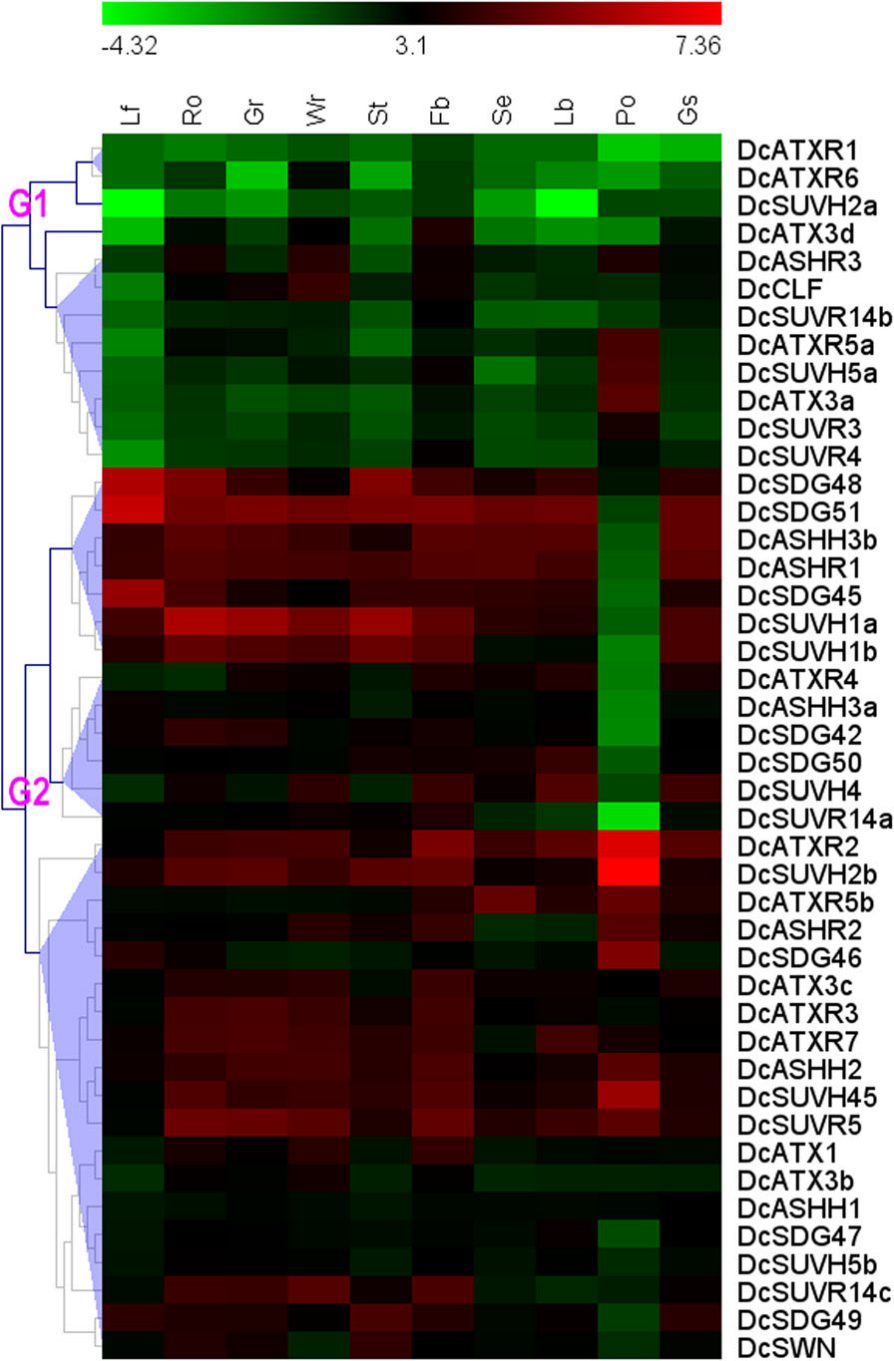
Tissue and organ expression profiles of DcSDGs across different tissues and organs. Hierarchical clustering of expression profiles of *DcSDGs* across different tissues and organs. Heat map generated using the software MultiExperiment Viewer (MeV) shows relative organ expression profiles of *DcSDG* genes in *D. catenatum*. Color scale at the top represents log 2 expression values, green and red represent low and high levels of transcript abundances, respectively. Lf: leaf, Ro: root, Gr: green root tip, Wr: white part of root, St: stem, Fb: flower bud, Se: sepal, Lb: labellum (lip), Po: pollinia, and Gs, gynostemium (column)

Based on hierarchical clustering (Fig. 6 and Additional file 3), the expression patterns of *DcSDGs* could be divided into two groups, G1 and G2. G1 genes usually feature low expressions in most tissues and organs. However, several genes are highly expressed in specific tissues and organs, such as *DcATX3d* in flower bud, *DcASHR3* in root and flower bud, and *DcATXR5a*, *DcSUVH5a*, and *DcATX3a* in pollinia. G2 genes display diverse expression profiles in different tissues and organs. The majority of G2 group genes are highly expressed in most of the detected tissues and organs, whereas several show intermediate expressions in most tissues and organs, such as *DcASHH3a*, *DcASHH1*, *DcSUVH5b*, and *DcSDG47*. Furthermore, ~ 86% of genes (38/44) present intermediate and high expressions in flower buds, compared with ~ 77% (34) in root and gynostemium, ~ 57% (25) in leaf, ~ 45% (20) in stem, ~ 43% (19) in pollinia, ~ 55% (24) in sepal, and ~ 64% (28) in labellum. These findings suggest that *DcSDG* family plays essential roles in flower bud formation. Notably, *DcSDG51* in leaf, *DcSUVH2b* in pollinia, and *DcSUVH1a* in root and stem display the most distinguished expressions, indicating their prominent functions in specific tissues.

The expression profiles of six duplicated *DcSDG* gene pairs were further compared (Additional file 4). In general, one copy showed higher expression levels than the others in all tissues, except for *DcSUVH5a/5b* pairs, suggesting that one paralog might performed a dominant function during plant growth and development. *DcASHH3a/3b*, *DcATX3b/3c*, *DcATXR5a/5b* and *DcSUVR14b/14c* exhibited similar expression patterns, whereas *DcSUVH5a/5b* pairs displayed differential expression profiles in the detected tissues and organs. These results indicate that distinct duplicated gene pairs might undergo different evolutionary pressures and diverge at varying periods.

### Expression levels of *DcSDGs* in response to environmental stresses

**Fig. 7:**
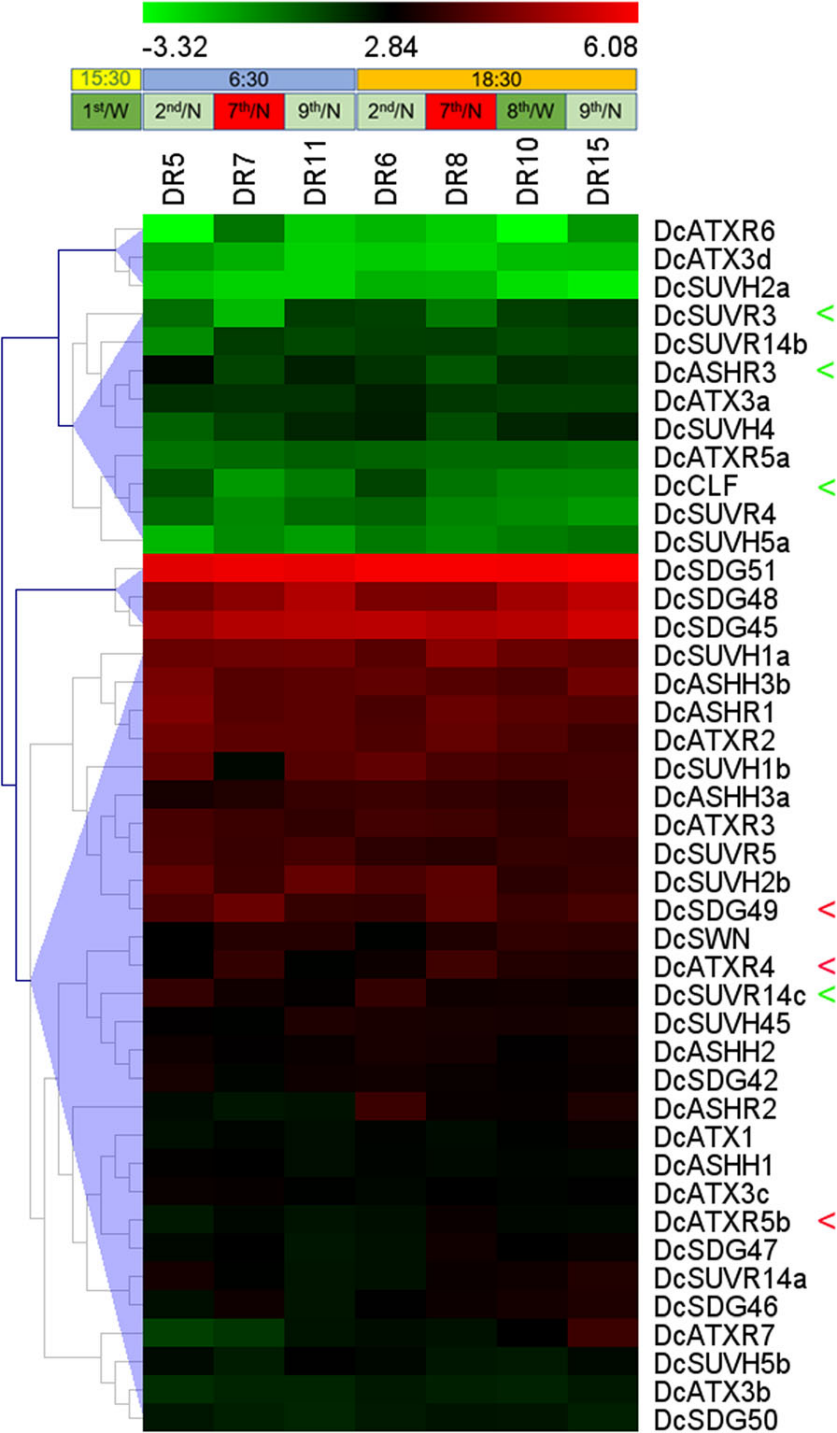
Expression of *DcSDGs* in response to drought stress. Heat map showing expression pattern of *DcSDG* genes in leaves under different drought treatments. The seedlings were watered on the 1st day, dried from the 2nd to the 7th day, and re-watered on the 8th day. Leaves were collected at different times; DR5/ DR8, DR6/DR10, and DR7/DR15 indicate sampling at 06:30 and 18:30 on the 2nd, 7th, and 9th days, respectively, and DR11 indicates sampling at 18:30 on the 8th day. The Y-axis represents the value of the relative expression level [log 2 (FPKM + 1)]

*D. catenatum* is an epiphytic orchid plant that grows on trunks and cliffs and often experiences diverse environmental stresses, such as drought, cold, and high temperature. To detect the responses of *DcSDG* genes to drought stress, the expression profiles of *DcSDGs* were assessed by analyzing the RNA-seq data from the leaves under different drought treatments [99] (Fig. 7 and Additional file 5). In brief, the seedlings were irrigated on the 1st day, and kept unwatered from the 2nd day to the 7th day, and recovered on the 8th day. Leaves were sampled at both 06:30 and 18:30 on the 2nd (DR5 and DR8), 7th (DR6 and DR10), and 9th (DR7 and DR15) days, respectively, and at 18:30 on the 8th day (DR11). The results showed that one-week of drought stress notably repressed the expressions of *DcCLF*, *DcASHR3*, *DcSUVR3*, and *DcSUVR14c*, but obviously induced the expression of *DcATXR5b*, *DcATXR4*, and *DcSDG49* when sampling at both dawn and dusk. Subsequently, rewatering restored the expression levels of *DcASHR3*, *DcSUVR3*, *DcATXR5b*, *DcATXR4*, and *DcSDG49*.

**Fig. 8:**
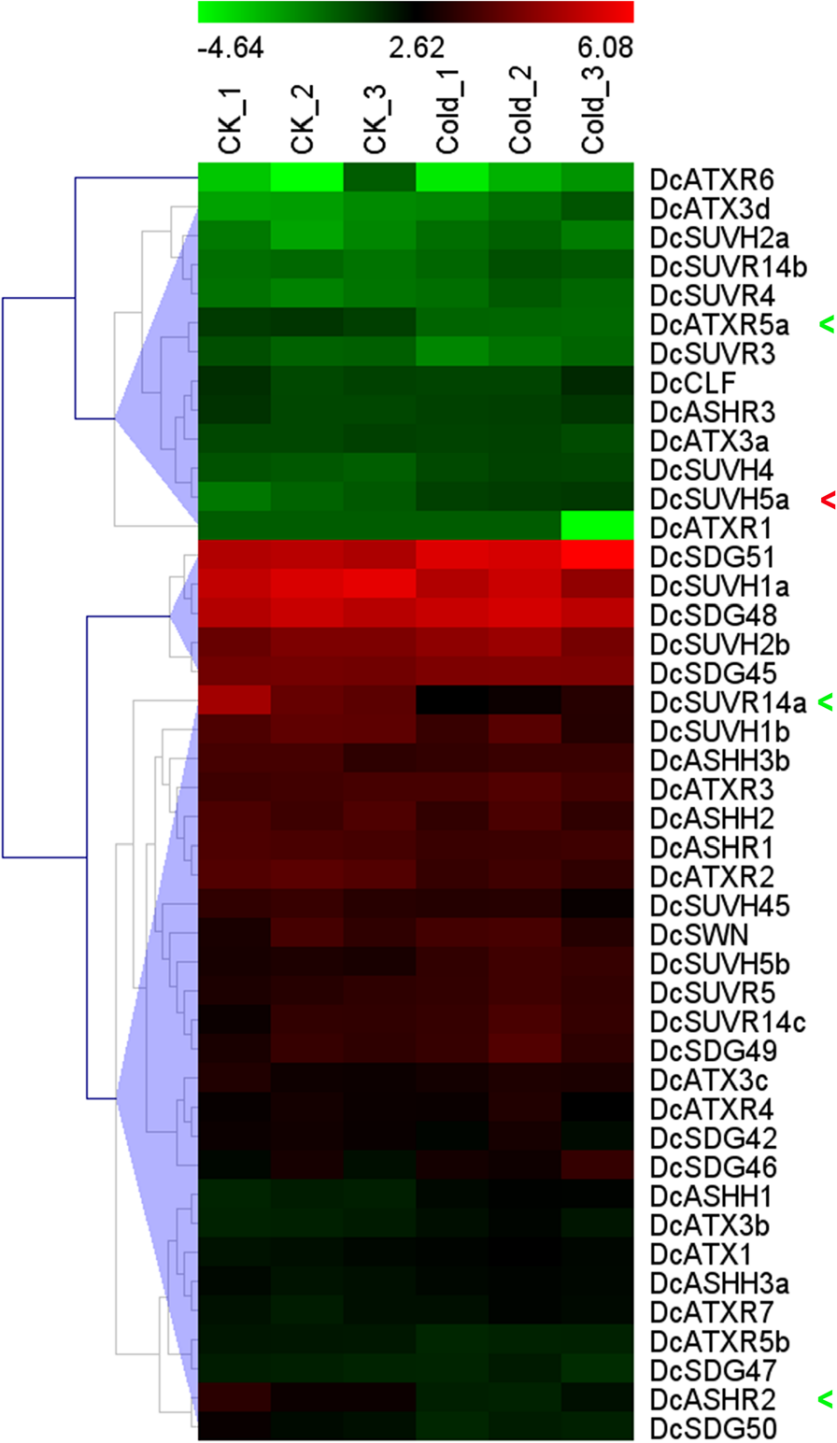
Expression of *DcSDGs* in response to cold stress. Heat map showing expression pattern of *DcSDG* genes in leaves under cold stress for 20 h. The Y-axis represents the value of the relative expression level [log 2 (FPKM + 1)]

To explore the possible roles of SDG proteins in response to cold stress, we evaluated the expression levels of *DcSDGs* through analyzing the raw RNA-seq reads from the leaves of *D. catenatum* seedlings treated at 20 °C (control) and 0 °C for 20 h, respectively [43] (Fig. 8 and Additional file 6). Data revealed that 32% of *DcSDG* genes (14) showed transcription change in response to cold stress. For example, genes with upregulated expression consisted of *DcASHH1* (II), *DcATX3b* (III), *DcSUVH4* (V), *DcSUVH5a* (V), *DcSUVH5b* (V), DcSDG45 (VII), and DcSDG51 (VII), whereas genes with deregulated expressions included *DcATXR5a* (IV), *DcATXR5b* (IV), *DcSUVR14a* (V), *DcASHR1* (VI), *DcASHR2* (VI), *DcATXR2* (VI), and *DcSDG50* (VII). The expression levels of *DcSUVH5a*, *DcATXR5a*, *DcASHR2* and *DcSUVR14a* (fold change > 2 or < 0.5) are significantly influenced by cold.

**Fig. 9:**
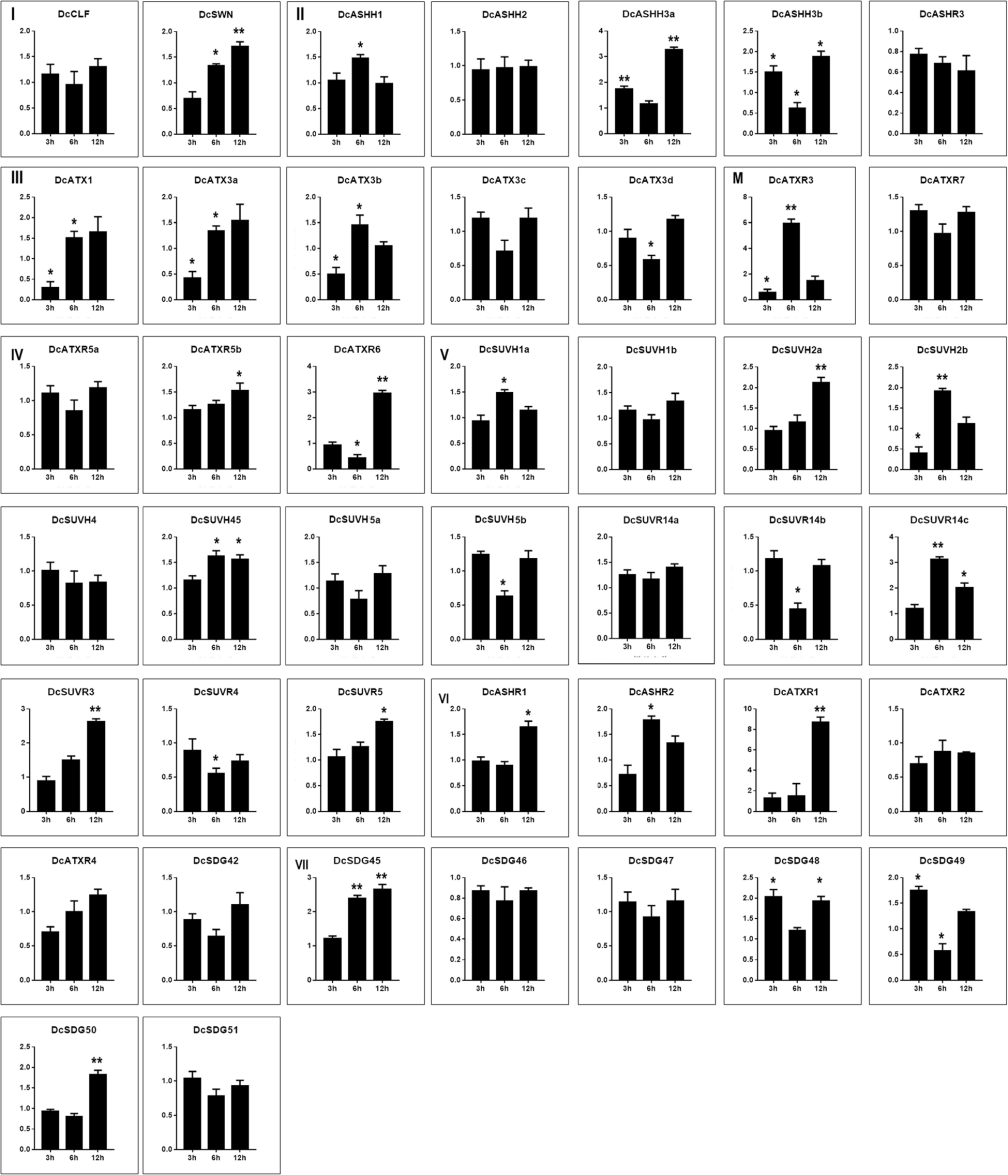
Expression of *DcSDGs* in response to high temperature stress through RT-qPCR assay. The actin gene of *D. catenatum* was used as an internal reference. The data are representative of three independent experiments. The error bars indicate SD and the asterisk shows the corresponding gene between the heat shock (35 °C) and the control (20 °C) significantly affected by Student′s *t* test (*, *P* < 0.05; **, *P* < 0.01)

To further understand the roles of DcSDG proteins in response to high temperature (35 °C) stress, the expression profiles of SDG genes in the leaves of *D. catenatum* seedlings were examined by quantitative *reverse-transcription*–polymerase chain reaction (RT-qPCR) (Fig. 9). The results show the diverse expression patterns of *DcSDG* genes during heat shock treatment. At 3 h after treatment (HAT), the number of upregulated genes (10) was slightly higher than that of downregulated genes (7). At 6 HAT, more *DcSDG* genes were induced (15 upregulated genes versus 10 downregulated genes). At 12 HAT, the number of upregulated genes (27) was evidently higher than that of downregulated genes (3). Of the genes examined upon exposure to heat shock, three Class II genes (*DcASHH3a/3b* and *DcATX3a*), five Class V genes (*DcSUVH2a/2b*, *DcSUR14b/14c*, and *DcSUVR3*), two Class VI genes (*DcATXR1* and DcASHR1), two Class VII genes (*DcSDG45/48*), and one Class M gene *DcATXR3* were distinguished from the corresponding control in at least at one time point (*P* < 0.05, Fig. 9).

## Discussion

### Characterization and classification of SDG proteins in *D. catenatum*

*D. catenatum* shows extensive application value in the food service industry, pharmaceutical, cosmetics, health products, and ornamental horticulture in China. The recent successful genome sequencing of *D. catenatum* associated with its unique developmental and living features promoted its use as a model orchid plant with considerable theoretical relevance [100]. In this study, 44 SDG proteins in *D. catenatum* was identified and divided into eight classes: Class-I (H3K27me2/3), Class-II (H3K36), Class-III (H3K4me2/3), Class-M (H3K4me), Class-IV (H3K27me1), Class-V (H3K9me), Class-VI/S-ET (undefined), Class-VII/ RBCMT (non-histone Rubisco).

To date, the understanding of Class VI and VII is highly limited. Class VI includes six clades (Additional file 7), whose members feature a long S-ET domain interrupted by a Zf-MYND domain and have not been functionally characterized in plants. In mammalian ASHR1 homologs, *SET And MYND Domain Containing 3* (SMYD3) may methylate histones H3K4 and H4K5, whereas SMYD2 can dimethylate H3K36 and repress gene transcription [101–103]. SMYD proteins also possess non-histone substrates, such as SMYD2 for p53 and estrogen receptor α and SMYD3 for VEGFR1 and MAP 3 K2, in the nucleus and cytoplasm [104–107]. Class VII consists of nine clades (Additional file 8), whose members are characterized by an additional Rubisco substrate-binding (Rubis-subs-bind) domain and conserved in plants and animals. In flowering plants, chloroplast-localized Rubisco large subunit N-methyltransferase (LSMT) performs the conserved and ancient functions to methylate the Rubisco small subunit and fructose-1,6-bisphosphate aldolase (FBA). Moreover, this enzyme evolved an extra novel role to catalyze the K14 trimethylation of Rubisco large subunit in Fabaceae, Cucurbitaceae, and Rosaceae [108, 109]. Based on the strict correlation between the presence of the His-Ala/Pro-Trp triad motif and the Rubisco methylation status [108], LSMTs in *D. catenatum* and the other three examined species only retained the ancestral function due to the absence of this triad motif. In animal, Class VII homolog SETD3 with additional Rubis-subs-bind domain exhibits a H3K36 methyltransferase activity [110]. Therefore, the members of Class VI and VII in plants may be involved in the methylation of both histone and non-histone proteins; such assumption is beyond the previous expectation.

### Evolution and function divergence of SDG proteins

Gene duplication, as a critical driving force, provides an extra copy of genetic material to tolerate random mutations to survive the natural selection and create new species during evolution. Gene duplication, including tandem duplication and polyploidy (whole genome duplication, WGD), considerably contributes to the formation of gene families [24], such as the SDG family in *D. catenatum*. *D. catenatum* has undergone at least four rounds of WGD events, including two ancient WGDs before (ζ) and after (ε) the angiosperm split from gymnosperm, a τ WGD shared by most monocots and a recent WGD specific to the Orchidaceae lineage [98, 111]. Duplicate genes usually undergo three evolutionary fates, nonfunctionalization with silencing function, neofunctionalization with novel function, and subfunctionalization with partial function [112]. *D. catenatum* contains six pairs of duplicated *DcSDG* genes. In *DcSUVH2a/2b* pairs, *DcSUVH2a* was unexpressed in all examined tissues, implying that neofunctionalization might have occurred. *DcASHH3a/3b*, *DcATX3b/3c*, *DcATXR5a/5b* and *DcSUVR14b/14c* pairs displayed similar expression patterns, indicating that they might have experienced subfunctionalization. *DcSUVH5a/5b* pairs exhibited differential expression profiles in different tissues and organs, indicating that novel expression specificity and function have developed.

### Involvement of SDG proteins in plant development programs and environmental stress responses

SDG proteins associated with the corresponding histone methylations can output precise developmental instructions through diverse mechanisms. (1) Different SDG proteins show similar catalytic specificity, but act on distinct sets of genes. As the writers of H3K4 methylation, ATX3/4/5 have different targets from *ATXR3*/*SDG2* [66]. (2) Some SDG members are involved in specific conserved complexes. Class I E(z) homologs (CLF, SWN and MEA), together with MSI1, FIE, and Su(z)12 homologs (FIS2, EMF2 and VRN2) constitute different PRC2 complexes, functioning in distinct developmental phases [113]. (3) Specific SDG proteins can interplay with other types of epigenetic regulators. SUVH4/5/6 interacts with the histone deacetylase HDA6 to silence a subset of transposons through histone H3K9 methylation and H3 deacetylation [114]. Further in-depth molecular dissection of SDG members in *D. catenatum* with specific developmental and living modes will enrich the action mechanisms of the SDG gene family.

Histone methylation established by SDG proteins is widely involved in responses to environmental stresses and pathogen challenges [27, 33, 35–38]. Studies have reported that drought stress can cause global changes of histone H3K4 methylation patterns in *Arabidopsis* and rice [37, 38]. Class III member ATX1 is implicated in drought stress response via both ABA-dependent and -independent pathways in Arabidopsis [35, 36]. Here we observed that the dynamic expression changes of *DcASHR3* (Class II), *DcSUVR3* (V), *DcATXR5b* (V), *DcATXR4* (VI), and *DcSDG49* (VII) were closely associated with drought-rewatering treatment, indicating that methylations of H3K36 and H3K9 are also involved in drought response. H3K36me3 and H3K27me3 have been proven to play antagonistic roles in the cold-induced epigenetic switch at the Arabidopsis *FLOWERING LOCUS C* (*FLC*) locus [115]. In *D. catenatum*, we identified six significant cold-responsive *DcSDG* genes, including *DcASHH1* (Class II), *DcATX3b/3d* (III), *DcATXR5a/5b* (Class M), *DcSUVH5a/5b* and *DcSUVR14a/3* (V) (Fig. 8). The results indicate diverse histone methylation marks with specific DcSDG proteins that perform certain roles during cold treatment. It will be intriguing to further investigate their direct targets by high-throughput ChIP-seq method combined with excellent commercial antibodies against various histone methylation marks. Recently, Huang et al. [24] thoroughly identified cotton *SDG* genes and noted that the expressions of most of these genes decreased under high-temperature conditions. In this study, 61% of *DcSDG* genes showed response to heat shock, but the number of upregulated genes was considerably higher than that of downregulated ones. This finding may be related to the particularly epiphytic lifestyle and crassulacean acid metabolism pathway in *D. catenatum*.

## Conclusions

In this study, we identified 44 SDG proteins in *D. catenatum* and 42 in its close relative *P. equestris*, and replenished three other SDG members (Os01g65730/OsSET44, Os01g74500/OsSET45, and Os06g03676/OsSET46) into the previous rice SDG gene family (43 members). Based on the phylogenetic relationship and substrate specificity, these genes were divided into eight classes by using well-characterized Arabidopsis SDG members as references. In addition, we analyzed the expression profiles of *D. catenatum SDG* genes in different tissues and organs and their responses to diverse environmental stresses. Our findings provide comprehensive information on the classification and expression profiles on *D. catenatum SDG* genes, and will lay the foundation for the functional characterization of the *SDG* gene family in orchids.

## Methods

### Identification of *SDG* gene family in *D. catenatum* and *P. equestris*

All the SDG sequences including genomic DNAs, CDS and proteins in *Oryza sativa* and *Arabidopsis thaliana* were retrieved from the plant genomics resource Phytozome v12 (http://phytozome.jgi.doe.gov/pz/portal.html). Of these, the protein sequences were used as queries to search homologs of *D. catenatum* and *P. equestris* against NCBI database using BLASTp tool. All the hits were further confirmed by the existence of the signature SET domain detected using on-line biosofts PROSITE (http://prosite.expasy.org/), SMART (http:// smart.embl-heidelberg.de/) and PFam (http://pfam.xfam.org/search).

### Analysis of gene structure, domain architecture and phylogenetic relationship

The gene structure including the intron-exon distribution pattern was reconstructed by Gene Structure Display Server GSDS 2.0 (http://gsds.cbi.pku.edu.cn/) [116]. The domain organization was analyzed using SMART and Pfam databases. Phylogenetic analysis was performed using MEGA7 [117]. The full-length amino acid sequences of SDG proteins were used for constructing neighbor-joining (NJ) trees with the following settings: pairwise deletion option for gaps/missing data treatment; p-distance method for Substitution model; and bootstrap test of 1000 replicates for evaluation of internal branch reliability.

### In silico expression profiling of *DcSDG* genes

For the tissue and organ expression profiling of *DcSDG* genes, The raw RNA-seq data of leaf (SRR4431601), root (SRX2938667), Green root tip (SRR4431599), white part of root (SRR4431598), stem (SRR4431600), flower bud (SRR4431603), sepal (SRR4431597), labellum (SRR4431602), pollinia (SRR5722145) and gynostemium (SRR4431596) in an individual of wild *D. catenatum* were downloaded from the NCBI Sequence Read Archive (SRA) provided by Zhang et al [98]*.* For drought stress and stress removal experiment in 8-month-old *D. catenatum* plants [99], irrigation was performed on the 1st day, omitted from the 2nd to the 7th day, and resumed on the 8th day, watering every 2 days at 15:30. Consequently, the raw RNA-seq reads were obtained from the leaves that were harvested at both 06:30 and 18:30 on the 2nd [DR5 (NCBI: SRR7223299) and DR8 (SRR7223300)], 7th [DR6 (SRR7223298) and DR10 (SRR7223296)], and 9th [DR7 (SRR7223301) and DR15 (SRR7223297)] days, respectively, and at 18:30 on the 8th day [DR11 (SRR7223295)]. For the expression analysis of *DcSDG* genes in response to cold stress, the raw RNA-seq reads of leaves under 20 °C control condition (SRR3210630, SRR3210635 and SRR3210636) and 0 °C cold treatment for 20 h (SRR3210613, SRR3210621 and SRR3210626) were obtained from NCBI provided by Wu et al [43]. Reads of all the samples were aligned to the NCBI *Dendrobium* reference genome using HISAT package [118] The mapped reads of each sample were assembled using StringTie [119]. Then, all transcriptomes from samples were merged to reconstruct a comprehensive transcriptome using perl scripts. After the final transcriptome was generated, StringTie and edgeR was used to estimate the expression levels of all transcripts. StringTie was used to perform expression level for mRNAs by calculating FPKM. *DcSDG* genes were selected and differentially expressed genes were defined with log2 (fold change) > 1 or < − 1 and with statistical significance (*p* value < 0.05) by R package. Heatmap was generated using TIGR MultiExperiment Viewer (MeV4.9) software [120].

### Plant material and heat shock treatment

*D. catenatum* cultivar “Jingpin NO. 1” (Breed NO. Zhe R-SV-DO-015-2014) was from the State Key Laboratory of Subtropical Silviculture in Zhejiang Province, China. *D. catenatum* was grown in greenhouse at 20 °C under a 12 h light/12 h dark regime. 1-year-old seedlings were treated at 35 °C heat shock for indicated time (3 h, 6 h and 12 h) in a temperature-controlled incubator, compared with the mock plants at 20 °C. Then the leaves were harvested, snap frozen in liquid nitrogen and stored at − 70 °C for further expression analysis.

### Real-time quantitative RT-PCR (RT-qPCR)

Total RNA was extracted from the leaves using TRIzol reagent (Invitrogen, USA) followed by RNase-free DNase I treatment. First strand cDNA synthesis was performed via PrimerScript RT Enzyme Mix I kit (TaKaRa, Japan), according to the manufacturer’s instructions. RT-qPCR reaction mixture (10 μl) was prepared according to the manual of SYBR® Premix Ex Taq™ II (Tli RNaseH Plus) kit (TaKaRa, Japan). Then the reaction was carried out on CFX96 Touch™ Real-Time PCR Detection System (BIO-RAD, USA) in three technical replicates for each biological triplicate using the primers listed in Additional file 9. The reaction condition was set as the following temperature profile: 94 °C for 3 m, 40 cycles of 94 °C for 20s, 60 °C for 20s, 72 °C for 20s. The constitutive *DcACTIN* was used as the reference gene. The expression value of each gene tested was normalized with the internal reference gene, and the relative expression level was calculated with 2^-ΔΔCT^ method [121].

## Supplementary information

**Additional file 1.** SDG protein sequences in Arabidopsis and rice retrieved from Phytozome 12 database.

**Additional file 2. **Identification and classification of SDG genes in *P. equestris*. Sequences and information of *P. equestris* SDG genes and proteins came from NCBI database (https://www.ncbi.nlm.nih.gov/).

**Additional file 3. **Expression data of *DcSDG* genes from different tissues and organs in *D. catenatum*. The FPKM values of *DcSDG* genes in different tissues and organs was used for expression analysis in Fig. 6. Lf: leaf, Ro: root, Gr: green root tip, Wr: white part of root, St: stem, Fb: flower bud, Se: sepal, Lb: labellum, Po: pollinia, and Gs, gynostemium.

**Additional file 4. **Expression patterns of duplicated *SDG* gene pairs. The FPKM values of the duplicated *DcSDG* genes in different tissues and organs was used for comparison. Lf: leaf, Ro: root, Gr: green root tip, Wr: white part of root, St: stem, Fb: flower bud, Se: sepal, Lb: labellum, Po: pollinia, and Gs, gynostemium.

**Additional file 5. **Expression data of *DcSDG* genes from different drought treatments. The FPKM values of *DcSDG* genes in leaves under different drought treatments were used for expression analysis in Fig. 7. The seedlings were watered on the 1st day, dried from the 2nd to the 7th day, and re-watered on the 8th day. Leaves were collected at different times; DR5/ DR8, DR6/DR10, and DR7/DR15 indicate sampling at 06:30 and 18:30 on the 2nd, 7th, and 9th days, respectively, and DR11 indicates sampling at 18:30 on the 8th day.

**Additional file 6. **Expression data of *DcSDG* genes in the absence and presence of cold treatment. The FPKM values of *DcSDGs* genes in leaves under cold stress / 20 °C (control) for 20 h were used for expression analysis in Fig. 8.

**Additional file 7. **Domain organization and gene structure of the class-VI DcSDGs. The NJ tree was generated using MEGA7 with parameter settings as Fig. 1 based on full-length amino acid sequences of Class-VI SDGs in *D. catenatum*, *P. equestris*, Arabidopsis and rice. The number along the tree branch indicates bootstrap value. Different conserved protein domains are colored as indicated. Gene structures of SDGs in each species were indicated in distinct colors. The solid boxes represent exons and black lines represent introns.

**Additional file 8. **Domain organization and gene structure of the class-VII DcSDGs. The NJ tree was generated using MEGA7 with parameter settings as Fig. 1 based on full-length amino acid sequences of Class-VII SDGs in *D. catenatum*, *P. equestris*, Arabidopsis and rice. The number along the tree branch indicates bootstrap value. Different conserved protein domains are colored as indicated. Gene structures of SDGs in each species were indicated in distinct colors. The solid boxes represent exons and black lines represent introns.

**Additional file 9.** Primers used for expression analysis of heat shock treatment in this study.

## Data Availability

All data generated or analyzed during this study are included in this published article and its Additional files. The datasets generated and analyzed during the current study are available from the corresponding author on reasonable request.

## Abbreviations

ABA: Abscisic acid
ATX1: *ARABIDOPSIS* TRITHORAX-LIKE PROTEIN1
ATXR3: ARABIDOPSIS TRITHORAX RELATED 3
ChIP-Seq: Chromatin immunoprecipitation-based sequencing
CLF: CURLY LEAF
E(Z): Zeste
FBA: Fructose-1,6-bisphosphate aldolase
FLC: FLOWERING LOCUS C
HAT: Hour after treatment
HKMTase: Histone lysine methyltransferase
JA: Jasmonic acid
KYP: KRYPTONITE
MEA: MEAEA
NJ: Neighbor-joining
PRC2: Polycomb Repressive Complex 2
RBCMT: Ribulose-1,5-bisphosphate carboxylase/oxygenase (Rubisco) methyltransferase
RdDM: RNA-directed DNA methylation
RT-qPCR: Real-time quantitative polymerase chain reaction
Rubis-subs-bind: Rubisco substrate-binding
SA: Salicylic acid
SDG: SET DOMAIN GROUP
SET: Su (var), E(z), and Trithorax
SMYD3: *SET and MYND domain containing 3*
SRA: SET- and RING-ASSOCIATED
Su (var): Suppressor of variegation
SWN: SWINGER
Trx: Trithorax
WGD: Whole genome duplication
