# Supplementary material for: Genome-wide identification and expression profiling of SET DOMAIN GROUP family in Dendrobium catenatum
Source: BMC Plant Biol. 2020 Jan 28;20:40. doi: 10.1186/s12870-020-2244-6 (PMC6986063; doi:10.1186/s12870-020-2244-6)
Supplement: Supplementary file 1 — Additional file 1. SDG protein sequences in Arabidopsis and rice retrieved from Phytozome 12 database. [file 12870_2020_2244_MOESM1_ESM.docx]

>AtSDG44_At1g01920

MAISEEEAKLERFLDWLQVNGGELRGCNIKYSDSLKGFGIFASTSTQASDEVLLVVPLDLAITPMRVLQDPLLGPECQKMFEQGQVDDRFLMILFLTLER

LRINSSWKPYLDMLPTRFGNPLWFSDDDILELKGTNLYHATELQKKKLLSLYHDKVEVLVTKLLILDGDSESKVSFEHFLWANSVFWSRALNIPLPHSFV

FPQSQDDTGECTSTSESPETAPVNSNEEKGKSLTSAQPAPSVGSGDTIWVEGLVPGIDFCNHDLKPVATWEVDGIGSVSRVPFSMYLLSVAQRPIPKKEI

SISYGNKGNEELLYLYGFVIDNNPDDYLMIKEMLVNFVLTSVVTFNNGFIQVHYPVEAIPSIPFSDSKGQLLEAQNAQLRCLLPKSVLNHGFFPRTTSVI

RESDEKETVRSCNFSWSGKRKMPTYMNKLVFPEDFMTGLRTIAMQEEEIYKVSAMLEELVESRQGEQPSETEVRMAVWEACGDSGALQLLVDLLNSKMMK

LEENSGTEEQDARLLEEACVLESHEESRDLDGRRMSRNKWSSVVYRRGQKQLTRLLLKEAEHALHLALSSDH

>AtMEA_SDG5

MEKENHEDDGEGLPPELNQIKEQIEKERFLHIKRKFELRYIPSVATHASHHQSFDLNQPAAEDDNGGDNKSLLSRMQNPLRHFSASSDYNSYEDQGYVLD

EDQDYALEEDVPLFLDEDVPLLPSVKLPIVEKLPRSITWVFTKSSQLMAESDSVIGKRQIYYLNGEALELSSEEDEEDEEEDEEEIKKEKCEFSEDVDRF

IWTVGQDYGLDDLVVRRALAKYLEVDVSDILERYNELKLKNDGTAGEASDLTSKTITTAFQDFADRRHCRRCMIFDCHMHEKYEPESRSSEDKSSLFEDE

DRQPCSEHCYLKVRSVTEADHVMDNDNSISNKIVVSDPNNTMWTPVEKDLYLKGIEIFGRNSCDVALNILRGLKTCLEIYNYMREQDQCTMSLDLNKTTQ

RHNQVTKKVSRKSSRSVRKKSRLRKYARYPPALKKTTSGEAKFYKHYTPCTCKSKCGQQCPCLTHENCCEKYCGCSKDCNNRFGGCNCAIGQCTNRQCPC

FAANRECDPDLCRSCPLSCGDGTLGETPVQIQCKNMQFLLQTNKKILIGKSDVHGWGAFTWDSLKKNEYLGEYTGELITHDEANERGRIEDRIGSSYLFT

LNDQLEIDARRKGNEFKFLNHSARPNCYAKLMIVRGDQRIGLFAERAIEEGEELFFDYCYGPEHADWSRGREPRKTGASKRSKEARPAR

>AtSUVR1_SDG13

MAPNLRIKKACDAMKLLGISETKTRAFLRKLLKTYENNWDFIEEDAYKVLLDAIFDEADAQSTEKNKKEEEKKKKEEEKKSRSVATSRGRRKAPEPLVQD

EEDDMDEDEFPLKRRLRSRRGRASSSSSSSSSYNNEDLKTQPEEEDEDDGVTELPPLKRYVRRNGERGLAMTVYNNASPSSSSRLSMEPEEVPPMVLLPA

HPMETKVSEASALVILNDEPNIDHKPVISDTGNCSAPMLEMGKSNIHVQEWDWETKDILNDTTAMDVSPSSAIGESSEHKVAAASVELASSTSGEAKICL

SFAPATGETTNLHLPSMEDLRRAMEEKCLKSYKIVHPEFSVLGFMKDMCSCYIDLAKNSTSQLLETETVCDMSKAGDESGAVGISMPLVVVPECEISGDG

WKAISNMKDITAGEENVEIPWVNEINEKVPSRFRYMPHSFVFQDAPVIFSLSSFSDEQSCSTSCIEDCLASEMSCNCAIGVDNGFAYTLDGLLKEEFLEA

RISEARDQRKQVLRFCEECPLERAKKVEILEPCKGHLKRGAIKECWFKCGCTKRCGNRVVQRGMHNKLQVFFTPNGKGWGLRTLEKLPKGAFICEYIGEI

LTIPELYQRSFEDKPTLPVILDAHWGSEERLEGDKALCLDGMFYGNISRFLNHRCLDANLIEIPVQVETPDQHYYHLAFFTTRDIEAMEELAWDYGIDFN

DNDSLMKPFDCLCGSRFCRNKKRSTKTMQILNKA

>AtATX2_SDG30

MISMSCVPKEEEGEDTQIKTELHDHAADNPVRYASLESVYSVSSSSSSLCCKTAAGSHKKVNALKLPMSDSFELQPHRRPEIVHVYCRRKRRRRRRRESF

LELAILQNEGVERDDRIVKIESAELDDEKEEENKKKKQKKRRIGNGELMKLGVDSTTLSVSATPPLRGCRIKAVCSGNKQDGSSRSKRNTVKNQEKVVTA

SATAKKWVRLSYDGVDPKHFIGLQCKVFWPLDAVWYPGSIVGYNVETKHHIVKYGDGDGEELALRREKIKFLISRDDMELLNMKFGTNDVVVDGQDYDEL

VILAASFEECQDFEPRDIIWAKLTGHAMWPAIIVDESVIVKRKGLNNKISGGRSVLVQFFGTHDFARIQVKQAVSFLKGLLSRSPLKCKQPRFEEAMEEA

KMYLKEYKLPGRMDQLQKVADTDCSERINSGEEDSSNSGDDYTKDGEVWLRPTELGDCLHRIGDLQIINLGRIVTDSEFFKDSKHTWPEGYTATRKFISL

KDPNASAMYKMEVLRDAESKTRPVFRVTTNSGEQFKGDTPSACWNKIYNRIKKIQIASDNPDVLGEGLHESGTDMFGFSNPEVDKLIQGLLQSRPPSKVS

QRKYSSGKYQDHPTGYRPVRVEWKDLDKCNVCHMDEEYENNLFLQCDKCRMMVHTRCYGQLEPHNGILWLCNLCRPVALDIPPRCCLCPVVGGAMKPTTD

GRWAHLACAIWIPETCLLDVKKMEPIDGVKKVSKDRWKLLCSICGVSYGACIQCSNNTCRVAYHPLCARAAGLCVELADEDRLFLLSMDDDEADQCIRLL

SFCKRHRQTSNYHLETEYMIKPAHNIAEYLPPPNPSGCARTEPYNYLGRRGRKEPEALAGASSKRLFVENQPYIVGGYSRHEFSTYERIYGSKMSQITTP

SNILSMAEKYTFMKETYRKRLAFGKSGIHGFGIFAKLPHRAGDMVIEYTGELVRPPIADKREHLIYNSMVGAGTYMFRIDNERVIDATRTGSIAHLINHS

CEPNCYSRVISVNGDEHIIIFAKRDVAKWEELTYDYRFFSIDERLACYCGFPRCRGVVNDTEAEERQANIHASRCELKEWTES

>AtSUVH7_SDG17

MDKSIPIKAIPVACVRPDLVDDVTKNTSTIPTMVSPVLTNMPSATSPLLMVPPLRTIWPSNKEWYDGDAGPSSTGPIKREASDNTNDTAHNTFAPPPEMV

IPLITIRPSDDSSNYSCDAGAGPSTGPVKRGRGRPKGSKNSTPTEPKKPKVYDPNSLKVTSRGNFDSEITEAETETGNQEIVDSVMMRFDAVRRRLCQIN

HPEDILTTASGNCTKMGVKTNTRRRIGAVPGIHVGDIFYYWGEMCLVGLHKSNYGGIDFFTAAESAVEGHAAMCVVTAGQYDGETEGLDTLIYSGQGGTD

VYGNARDQEMKGGNLALEASVSKGNDVRVVRGVIHPHENNQKIYIYDGMYLVSKFWTVTGKSGFKEFRFKLVRKPNQPPAYAIWKTVENLRNHDLIDSRQ

GFILEDLSFGAELLRVPLVNEVDEDDKTIPEDFDYIPSQCHSGMMTHEFHFDRQSLGCQNCRHQPCMHQNCTCVQRNGDLLPYHNNILVCRKPLIYECGG

SCPCPDHCPTRLVQTGLKLHLEVFKTRNCGWGLRSWDPIRAGTFICEFAGLRKTKEEVEEDDDYLFDTSKIYQRFRWNYEPELLLEDSWEQVSEFINLPT

QVLISAKEKGNVGRFMNHSCSPNVFWQPIEYENRGDVYLLIGLFAMKHIPPMTELTYDYGVSCVERSEEDEVLLYKGKKTCLCGSVKCRGSFT

>AtATXR1_SDG35

MRGEQFELEEDRDGPLELLQSLRSKATELLLREEWEESIKVYTEFIDLSRRQVSSTGGSDPDPDSIAKLRKSLCLALCNRAEARARLRDFLEAMRDCDQA

LEIEKTHFKTLLCKGKVLLGLSKYSLALECFKTALLDPQASDNLETVTVYIEKCKKLEFQAKTGAFDLSDWILSEFRGKCPELAEFIGSIEIKKSELSGR

GLFATKNIVAGTLVLVTKAVAIERGILGNGECGEKAQLIMWKNFVEEVTESVRKCGRTRRVVSALSTGQGEDSLEIPEIALFRPDEAFETCGDWKQSLDT

EKLLSILDVNSLVEDAVSGKVMGKNKEYYGVGLWTLASFINHSCIPNARRLHVGDYVIVHASRDIKTGEEISFAYFDVLSPLEKRKEMAESWGFCCGCSR

CKFESVLYATNQEVREFEMGLERGVDAGNAVYMVEEGMKRWKVKGKDKGLLRASYWGVYDEIYNSERLMKRWGRKIPTMEVVVDSVSDVVGSDERLMKMA

VEGMMKKHGGFSNIVEMEKIMKLGKGVYGKVVSKKKAMKTLLGIE

>AtSDG41_At1g43245

MEIRAAEDIEIRTDLFPPLSPLASSLYDSFLSSHCSSCFSLLPPSPPQPLYCSAACSLTDSFTNSPQFPPEITPILPSDIRTSLHLLNSTAVDTSSSPHR

LNNLLTNHHLLMADPSISVAIHHAANFIATVIRSNRKNTELEEAAICAVLTNAVEVHDSNGLALGIALYNSSFSWINHSCSPNSCYRFVNNRTSYHDVHV

TNTETSSNLELQEQVCGTSLNSGNGNGPKLIVRSIKRIKSGEEITVSYIDLLQPTGLRQSDLWSKYRFMCNCGRCAASPPAYVDSILEGVLTLESEKTTV

GHFDGSTNKDEAVGKMNDYIQEAIDDFLSDNIDPKTCCEMIESVLHHGIQFKEDSQPHCLRLHACHYVALNAYITLATAYRIRSIDSETGIVCDMSRISA

AYSLFLAGVSHHLFCAERSFAISAAKFWKNAGELLFDLAPKLLMELSVESDVKCTKCLMLETSNSHRDIKEKSRQILSCVRDISQVTWSFLTRGCPYLEK

FRSPVDFSLTRTNGEREESSKDQTVNVLLLSSHCLLYADLLTDLCYGQKSHLVSRFRL

>AtSUVH3_SDG19

MQGVPGFNTVPNPNHYDKSIVLDIKPLRSLKPVFPNGNQGPPFVGCPPFGPSSSEYSSFFPFGAQQPTHDTPDLNQTQNTPIPSFVPPLRSYRTPTKTNG

PSSSSGTKRGVGRPKGTTSVKKKEKKTVANEPNLDVQVVKKFSSDFDSGISAAEREDGNAYLVSSVLMRFDAVRRRLSQVEFTKSATSKAAGTLMSNGVR

TNMKKRVGTVPGIEVGDIFFSRIEMCLVGLHMQTMAGIDYIISKAGSDEESLATSIVSSGRYEGEAQDPESLIYSGQGGNADKNRQASDQKLERGNLALE

NSLRKGNGVRVVRGEEDAASKTGKIYIYDGLYSISESWVEKGKSGCNTFKYKLVRQPGQPPAFGFWKSVQKWKEGLTTRPGLILPDLTSGAESKPVSLVN

DVDEDKGPAYFTYTSSLKYSETFKLTQPVIGCSCSGSCSPGNHNCSCIRKNDGDLPYLNGVILVSRRPVIYECGPTCPCHASCKNRVIQTGLKSRLEVFK

TRNRGWGLRSWDSLRAGSFICEYAGEVKDNGNLRGNQEEDAYVFDTSRVFNSFKWNYEPELVDEDPSTEVPEEFNLPSPLLISAKKFGNVARFMNHSCSP

NVFWQPVIREGNGESVIHIAFFAMRHIPPMAELTYDYGISPTSEARDESLLHGQRTCLCGSEQCRGSFG

>AtASHH1_SDG26

MQFSCDPDQEGDELPQYEHIYQNDFSYRKHKKQKEEDISICECKFDFGDPDSACGERCLNVITNTECTPGYCPCGVYCKNQKFQKCEYAKTKLIKCEGRG

WGLVALEEIKAGQFIMEYCGEVISWKEAKKRAQTYETHGVKDAYIISLNASEAIDATKKGSLARFINHSCRPNCETRKWNVLGEVRVGIFAKESISPRTE

LAYDYNFEWYGGAKVRCLCGAVACSGFLGAKSRGFQEDTYVWEDGDDRYSVDKIPVYDSAEDELTSEPSKNGESNTNEEKEKDISTENHLESTALNIQQQ

SDSTPTPMEEDVVTETVKTETSEDMKLLSQNSQEDSSPKTAIVSRVHGNISKIKSESLPKKRGRPFSGGKTKNVAQKHVDIANVVQLLATKEAQDEVLKY

EEVKKEAAVRLSSLYDEIRPAIEEHERDSQDSVATSVAEKWIQASCNKLKAEFDLYSSVIKNIASTPIKPQDTKTKVAEAGNEDHIKLLEAK

>AtASHH2_SDG8

MDCKENGVGDASGCNIDANSLASNLAMNTNEDFYEKLSSRGQNLDSVSSLEIPQTASSVNHTIEGQRKCFTEIEQMGYGNSNSQEDAGNTDDDLYVCYNA

DDTQEQGVVSGELEQSQELICDTDLLVNCNKLDDGKESQDTNVSLVSIFSGSMQEKEAPQAKEDEGYGGTTLPIGGSGIDTESTFVNDAPEQFESLETTK

HIKPDEVESDGISYRFDDGGKEGRNGPSSDLDTGSSDDISLSQSFSFPDSLLDSSVFGCSATESYLEDAIDIEGNGTIVVSPSLAITEMLNNDDGGLCSH

DLNKITVTETINPDLKLVREDRLDTDLSVMNEKMLKNHVGDSSSESAVAALSMNNGMAADLRAENFSQSSPIDEKTLDMEANSPITDSSLIWNFPLNFGS

GGIEVCNPENAVEPLRIVDDNGRIGGEVASASGSDFCEAGMSSSRRKARDGKQCKVVQTKTSARHLRKSSRKKQSERDIESIFKCSKQKRSSLLKTSRSS

EWGLPSKTTEIFLQSNNIPYDGPPHHEPQRSQGNLNNGEHNRSSHNGNVEGSNRNIQASSGSCLRLKVKFGKSGGQNPLNITVSKVSGNSLPGNGIVKAG

TCLELPGSAHFGEDKMQTVETKEDLVEKSNPVEKVSYLQSSDSMRDKKYNQDAGGLCRKVGGDVLDDDPHLSSIRMVEECERATGTQSLDAETSPDSEVI

NSVPDSIVNIEHKEGLHHGFFSTPEDVVKKNRVLEKEDELRASKSPSENGSHLIPNAKKAKHPKSKSNGTKKGKSKFSESAKDGRKNESHEGVEQRKSLN

TSMGRDDSDYPEVGRIESHKTTGALLDADIGKTSATYGTISSDVTHGEMVVDVTIEDSYSTESAWVRCDDCFKWRRIPASVVGSIDESSRWICMNNSDKR

FADCSKSQEMSNEEINEELGIGQDEADAYDCDAAKRGKEKEQKSKRLTGKQKACFKAIKTNQFLHRNRKSQTIDEIMVCHCKPSPDGRLGCGEECLNRML

NIECLQGTCPAGDLCSNQQFQKRKYVKFERFQSGKKGYGLRLLEDVREGQFLIEYVGEVLDMQSYETRQKEYAFKGQKHFYFMTLNGNEVIDAGAKGNLG

RFINHSCEPNCRTEKWMVNGEICVGIFSMQDLKKGQELTFDYNYVRVFGAAAKKCYCGSSHCRGYIGGDPLNGDVIIQSDSDEEYPELVILDDDESGEGI

LGATSRTFTDDADEQMPQSFEKVNGYKDLAPDNTQTQSSVSVKLPEREIPPPLLQPTEVLKELSSGISITAVQQEVPAEKKTKSTSPTSSSLSRMSPGGT

NSDKTTKHGSGEDKKILPRPRPRMKTSRSSESSKRDKGGIYPGVNKAQVIPVNKLQQQPIKSKGSEKVSPSIETFEGKLNELLDAVGGISKRRDSAKGYL

KLLLLTAASRGTDEEGIYSNRDLSMILDALLKTKSKSVLVDIINKNGLQMLHNIMKQYRGDFKRIPIIRKLLKVLEYLATRKILALEHIIRRPPFAGMES

FKDSVLSFTEHDDYTVHNIARSFRDRWIPKHFRKPWRINREERSESMRSPINRRFRASQEPRYDHQSPRPAEPAASVTSSKAATPETASVSEGYSEPNSG

LPETNGRKRKSRWDQPSKTKEQRIMTILSQQTDETNGNQDVQDDLPPGFSSPCTDVPDAITAQPQQKFLSRLPVSYGIPLSIVHQFGSPGKEDPTTWSVA

PGMPFYPFPPLPPVSHGEFFAKRNVRACSSSMGNLTYSNEILPATPVTDSTAPTRKRELFSSDIGTTYFRQQKQSVPPWLRNNGGEKTANSPIPGNLTLE

KKLNS

>AtSUVH10_SDG11

MGLVGLHSGTIDMEFIGVEDHGDEEGKQIAVSVISSGKNADKTEDPDSLIFTGFGGTDMYHGQPCNQKLERLNIPLEAAFRKKSIVRVVRCMKDEKRTNG

NIYIYDGTYMITNRWEEEGQNGFIVFKFKLVREPDQKPAFGIWKSIQNWRNGLSIRPGLILEDLSNGAENLKVCLVNEVDKENGPALFRYVTSLIHEVIN

NIPSMVDRCACGRRSCGSKHVFREKLSVSSSLVISAKKSGNVARFMNHSCSPNVFWQSIAREQNGLWCLYIGFFAMKHIPPLTELRYDYGKSRGGGKKMC

LCRTKKCCGSFG

>AtASHR1_SDG37

MADLQRFLQDRCLGVSNLPQKGRSLFTARDFRPGEVILSQKPYICVPNNTSSESRCDGCFKTNNLKKCSACQVVWYCGSSCQKSEWKLHRDECKALTRLE

KEKRKFVTPTIRLMVRLYIKRNLQNEKVLPITTTDNYSLVEALVSHMSEIDEKQMLLYAQMANLVNLILQFPSVDLREIAENFSKFSCNAHSICDSELRP

QGIGLFPLVSIINHSCSPNAVLVFEEQMAVVRAMDNISKDSEITISYIETAGSTLTRQKSLKEQYLFHCQCARCSNFGKPHDIEESAILEGYRCANEKCT

GFLLRDPEEKGFVCQKCLLLRSKEEVKKLASDLKTVSEKAPTSPSAEDKQAAIELYKTIEKLQVKLYHSFSIPLMRTREKLLKMLMDVEIWREALNYCRL

IVPVYQRVYPATHPLIGLQFYTQGKLEWLLGETKEAVSSLIKAFDILRISHGISTPFMKELSAKLEEARAEASYKQLALH

>AtSDG47_At2g18850

MVGALNIMSETGDLCVELPKDDPFYHHKKKFLSCKGLCVKETLNLSGSLSQQLLNAALEKLLHFGRIVNLDKVEVYFGEDACTPAGIYSVRNEISALSWI

LSLIPVSCKMQTQVDTFEALRAALKGRINEVVGAEKEKARVVDSYRCEKESKLVEWGQDNGVKTKLQIAQIDGYGRGAIASEDLKFGDVALEIPVSSIIS

EEYVYNSDMYPILETFDGITSETMLLLWTMREKHNLDSKFKPYFDSLQENFCTGLSFGVDAIMELDGTLLLDEIMQAKELLRERYDELIPLLSNHREVFP

PELYTWEHYLWACELYYSNSMQIKFPDGKLKTCLIPVAGFLNHSIYPHIVKYGKVDIETSSLKFPVSRPCNKGEQCFLSYGNYSSSHLLTFYGFLPKGDN

PYDVIPLDFDVIDDEDIETEFSWTTHMLRGTWLSSNHNIFHYGLPTPLLNYLRKAHGLVHHSETDLWKNLEVEIGVLENLQSTFDDMMQNLGDADSIDRE

NADWDVKLAMEFKERQRKIVSSILDSCSAGIKLVQESITNPPV

>AtASHR2_SDG39

MINDGGAKPETLLRVAEIGGRGRSLVAAQSLRAGQVILRESPLLLYSAFPFLSSSVSPYCDHCFRLLASSAHQKCQSCSLVSFCSPNCFASHTPWLCESL

RRLHQSSSSAFSDQPSDRQVQARFLLSAYNLAAASPSDFQILLSLQGSGSSNGDPSCSAGDSAAAGFLHSLLSSVCPSLPVSISPDLTAALLSKDKVNAF

GLMEPCSVSNEKRSVRAYGIYPKTSFFNHDCLPNACRFDYVDSASDGNTDIIIRMIHDVPEGREVCLSYFPVNMNYSSRQKRLLEDYGFKCDCDRCKVEF

SWSEGEEDENEIMEEMEDQDEQEEMEDSVGENEEEVCGNGVDDESNFPHAYFFVRYMCEKENCFGTLAPLPPKTHDASRVLECNVCGSVKEDEVGVNQ

>AtSUVH6_SDG23

MEMGVMENLMVHTEISKVKSQSNGEVEKRGVSVLENGGVCKLDRMSGLKFKRRKVFAVRDFPPGCGSRAMEVKIACENGNVVEDVKVVESLVKEEESLGQ

RDASENVSDIRMAEPVEVQPLRICLPGGDVVRDLSVTAGDECSNSEQIVAGSGVSSSSGTENIVRDIVVYADESSLGMDNLDQTQPLEIEMSDVAVAKPR

LVAGRKKAKKGIACHSSLKVVSREFGEGSRKKKSKKNLYWRDRESLDSPEQLRILGVGTSSGSSSGDSSRNKVKETLRLFHGVCRKILQEDEAKPEDQRR

KGKGLRIDFEASTILKRNGKFLNSGVHILGEVPGVEVGDEFQYRMELNILGIHKPSQAGIDYMKYGKAKVATSIVASGGYDDHLDNSDVLTYTGQGGNVM

QVKKKGEELKEPEDQKLITGNLALATSIEKQTPVRVIRGKHKSTHDKSKGGNYVYDGLYLVEKYWQQVGSHGMNVFKFQLRRIPGQPELSWVEVKKSKSK

YREGLCKLDISEGKEQSPISAVNEIDDEKPPLFTYTVKLIYPDWCRPVPPKSCCCTTRCTEAEARVCACVEKNGGEIPYNFDGAIVGAKPTIYECGPLCK

CPSSCYLRVTQHGIKLPLEIFKTKSRGWGVRCLKSIPIGSFICEYVGELLEDSEAERRIGNDEYLFDIGNRYDNSLAQGMSELMLGTQAGRSMAEGDESS

GFTIDAASKGNVGRFINHSCSPNLYAQNVLYDHEDSRIPHVMFFAQDNIPPLQELCYDYNYALDQVRDSKGNIKQKPCFCGAAVCRRRLY

>AtCLF_SDG1

MASEASPSSSATRSEPPKDSPAEERGPASKEVSEVIESLKKKLAADRCISIKKRIDENKKNLFAITQSFMRSSMERGGSCKDGSDLLVKRQRDSPGMKSG

IDESNNNRYVEDGPASSGMVQGSSVPVKISLRPIKMPDIKRLSPYTTWVFLDRNQRMTEDQSVVGRRRIYYDQTGGEALICSDSEEEAIDDEEEKRDFLE

PEDYIIRMTLEQLGLSDSVLAELASFLSRSTSEIKARHGVLMKEKEVSESGDNQAESSLLNKDMEGALDSFDNLFCRRCLVFDCRLHGCSQDLIFPAEKP

APWCPPVDENLTCGANCYKTLLKSGRFPGYGTIEGKTGTSSDGAGTKTTPTKFSSKLNGRKPKTFPSESASSNEKCALETSDSENGLQQDTNSDKVSSSP

KVKGSGRRVGRKRNKNRVAERVPRKTQKRQKKTEASDSDSIASGSCSPSDAKHKDNEDATSSSQKHVKSGNSGKSRKNGTPAEVSNNSVKDDVPVCQSNE

VASELDAPGSDESLRKEEFMGETVSRGRLATNKLWRPLEKSLFDKGVEIFGMNSCLIARNLLSGFKSCWEVFQYMTCSENKASFFGGDGLNPDGSSKFDI

NGNMVNNQVRRRSRFLRRRGKVRRLKYTWKSAAYHSIRKRITEKKDQPCRQFNPCNCKIACGKECPCLLNGTCCEKYCGCPKSCKNRFRGCHCAKSQCRS

RQCPCFAADRECDPDVCRNCWVIGGDGSLGVPSQRGDNYECRNMKLLLKQQQRVLLGISDVSGWGAFLKNSVSKHEYLGEYTGELISHKEADKRGKIYDR

ENCSFLFNLNDQFVLDAYRKGDKLKFANHSPEPNCYAKVIMVAGDHRVGIFAKERILAGEELFYDYRYEPDRAPAWAKKPEAPGSKKDENVTPSVGRPKK

LA

>AtSUVR5_SDG6

MEVKMDELVLDVDVEEATGSELLVKSEPEADLNAVKSSTDLVTVTGPIGKNGEGESSPSEPKWLQQDEPIALWVKWRGKWQAGIRCAKADWPLTTLRGKP

THDRKKYCVIFFPHTKNYSWADMQLVRSINEFPDPIAYKSHKIGLKLVKDLTAARRYIMRKLTVGMFNIVDQFPSEVVSEAARDIIIWKEFAMEATRSTS

YHDLGIMLVKLHSMILQRYMDPIWLENSFPLWVQKCNNAVNAESIELLNEEFDNCIKWNEVKSLSESPMQPMLLSEWKTWKHDIAKWFSISRRGVGEIAQ

PDSKSVFNSDVQASRKRPKLEIRRAETTNATHMESDTSPQGLSAIDSEFFSSRGNTNSPETMKEENPVMNTPENGLDLWDGIVVEAGGSQFMKTKETNGL

SHPQDQHINESVLKKPFGSGNKSQQCIAFIESKGRQCVRWANEGDVYCCVHLASRFTTKSMKNEGSPAVEAPMCGGVTVLGTKCKHRSLPGFLYCKKHRP

HTGMVKPDDSSSFLVKRKVSEIMSTLETNQCQDLVPFGEPEGPSFEKQEPHGATSFTEMFEHCSQEDNLCIGSCSENSYISCSEFSTKHSLYCEQHLPNW

LKRARNGKSRIISKEVFVDLLRGCLSREEKLALHQACDIFYKLFKSVLSLRNSVPMEVQIDWAKTEASRNADAGVGEFLMKLVSNERERLTRIWGFATGA

DEEDVSLSEYPNRLLAITNTCDDDDDKEKWSFSGFACAICLDSFVRRKLLEIHVEERHHVQFAEKCMLLQCIPCGSHFGDKEQLLVHVQAVHPSECKSLT

VASECNLTNGEFSQKPEAGSSQIVVSQNNENTSGVHKFVCKFCGLKFNLLPDLGRHHQAEHMGPSLVGSRGPKKGIRFNTYRMKSGRLSRPNKFKKSLGA

VSYRIRNRAGVNMKRRMQGSKSLGTEGNTEAGVSPPLDDSRNFDGVTDAHCSVVSDILLSKVQKAKHRPNNLDILSAARSACCRVSVETSLEAKFGDLPD

RIYLKAAKLCGEQGVQVQWHQEGYICSNGCKPVKDPNLLHPLIPRQENDRFGIAVDAGQHSNIELEVDECHCIMEAHHFSKRPFGNTAVLCKDISFGKES

VPICVVDDDLWNSEKPYEMPWECFTYVTNSILHPSMDLVKENLQLRCSCRSSVCSPVTCDHVYLFGNDFEDARDIYGKSMRCRFPYDGKQRIILEEGYPV

YECNKFCGCSRTCQNRVLQNGIRAKLEVFRTESKGWGLRACEHILRGTFVCEYIGEVLDQQEANKRRNQYGNGDCSYILDIDANINDIGRLMEEELDYAI

DATTHGNISRFINHSCSPNLVNHQVIVESMESPLAHIGLYASMDIAAGEEITRDYGRRPVPSEQENEHPCHCKATNCRGLLS

>AtSUVH8_SDG21

MVSTPPTLLMLFDDGDAGPSTGLVHREKSDAVNEEAHATSVPPHAPPQTLWLLDNFNIEDSYDRDAGPSTGPVHRERSDAVNEEAHATSIPPHAPPQTLW

LLDNFNIEDSYDRDAGPSTSPIDREASHEVNEDAHATSAPPHVMVSPLQNRRPFDQFNNQPYDASAGPSTGPGKRGRGRPKGSKNGSRKPKKPKAYDNNS

TDASAGPSSGLGKRRCGRPKGLKNRSRKPKKPKADDPNSKMVISCPDFDSRITEAERESGNQEIVDSILMRFDAVRRRLCQLNYRKDKILTASTNCMNLG

VRTNMTRRIGPIPGVQVGDIFYYWCEMCLVGLHRNTAGGIDSLLAKESGVDGPAATSVVTSGKYDNETEDLETLIYSGHGGKPCDQVLQRGNRALEASVR

RRNEVRVIRGELYNNEKVYIYDGLYLVSDCWQVTGKSGFKEYRFKLLRKPGQPPGYAIWKLVENLRNHELIDPRQGFILGDLSFGEEGLRVPLVNEVDEE

DKTIPDDFDYIRSQCYSGMTNDVNVDSQSLVQSYIHQNCTCILKNCGQLPYHDNILVCRKPLIYECGGSCPTRMVETGLKLHLEVFKTSNCGWGLRSWDP

IRAGTFICEFTGVSKTKEEVEEDDDYLFDTSRIYHSFRWNYEPELLCEDACEQVSEDANLPTQVLISAKEKGNVGRFMNHNCWPNVFWQPIEYDDNNGHI

YVRIGLFAMKHIPPMTELTYDYGISCVEKTGEDEVIYKGKKICLCGSVKCRGSFG

>AtATX1_SDG27

MACFSNETQIEIDVHDLVEAPIRYDSIESIYSIPSSALCCVNAVGSHSLMSKKVKAQKLPMIEQFEIEGSGVSASDDCCRSDDYKLRIQRPEIVRVYYRR

RKRPLRECLLDQAVAVKTESVELDEIDCFEEKKRRKIGNCELVKSGMESIGLRRCKENNAFSGNKQNGSSRRKGSSSKNQDKATLASRSAKKWVRLSYDG

VDPTSFIGLQCKVFWPLDALWYEGSIVGYSAERKRYTVKYRDGCDEDIVFDREMIKFLVSREEMELLHLKFCTSNVTVDGRDYDEMVVLAATLDECQDFE

PGDIVWAKLAGHAMWPAVIVDESIIGERKGLNNKVSGGGSLLVQFFGTHDFARIKVKQAISFIKGLLSPSHLKCKQPRFEEGMQEAKMYLKAHRLPERMS

QLQKGADSVDSDMANSTEEGNSGGDLLNDGEVWLRPTEHVDFRHIIGDLLIINLGKVVTDSQFFKDENHIWPEGYTAMRKFTSLTDHSASALYKMEVLRD

AETKTHPLFIVTADSGEQFKGPTPSACWNKIYNRIKKVQNSDSPNILGEELNGSGTDMFGLSNPEVIKLVQDLSKSRPSSHVSMCKNSLGRHQNQPTGYR

PVRVDWKDLDKCNVCHMDEEYENNLFLQCDKCRMMVHAKCYGELEPCDGALWLCNLCRPGAPDMPPRCCLCPVVGGAMKPTTDGRWAHLACAIWIPETCL

SDVKKMEPIDGVNKVSKDRWKLMCTICGVSYGACIQCSNNSCRVAYHPLCARAAGLCVELENDMSVEGEEADQCIRMLSFCKRHRQTSTACLGSEDRIKS

ATHKTSEYLPPPNPSGCARTEPYNCFGRRGRKEPEALAAASSKRLFVENQPYVIGGYSRLEFSTYKSIHGSKVSQMNTPSNILSMAEKYRYMRETYRKRL

AFGKSGIHGFGIFAKLPHRAGDMMIEYTGELVRPSIADKREQLIYNSMVGAGTYMFRIDDERVIDATRTGSIAHLINHSCVPNCYSRVITVNGDEHIIIF

AKRHIPKWEELTYDYRFFSIGERLSCSCGFPGCRGVVNDTEAEEQHAKICVPRCDLIDWTAE

>AtSUVH2_SDG3

MSTLLPFPDLNLMPDSQSSTAGTTAGDTVVTGKLEVKSEPIEEWQTPPSSTSDQSANTDLIAEFIRISELFRSAFKPLQVKGLDGVSVYGLDSGAIVAVP

EKENRELIEPPPGFKDNRVSTVVVSPKFERPRELARIAILGHEQRKELRQVMKRTRMTYESLRIHLMAESMKNHVLGQGRRRRSDMAAAYIMRDRGLWLN

YDKHIVGPVTGVEVGDIFFYRMELCVLGLHGQTQAGIDCLTAERSATGEPIATSIVVSGGYEDDEDTGDVLVYTGHGGQDHQHKQCDNQRLVGGNLGMER

SMHYGIEVRVIRGIKYENSISSKVYVYDGLYKIVDWWFAVGKSGFGVFKFRLVRIEGQPMMGSAVMRFAQTLRNKPSMVRPTGYVSFDLSNKKENVPVFL

YNDVDGDQEPRHYEYIAKAVFPPGIFGQGGISRTGCECKLSCTDDCLCARKNGGEFAYDDNGHLLKGKHVVFECGEFCTCGPSCKSRVTQKGLRNRLEVF

RSKETGWGVRTLDLIEAGAFICEYAGVVVTRLQAEILSMNGDVMVYPGRFTDQWRNWGDLSQVYPDFVRPNYPSLPPLDFSMDVSRMRNVACYISHSKEP

NVMVQFVLHDHNHLMFPRVMLFALENISPLAELSLDYGLADEVNGKLAICN

>AtSUVH5_SDG9

MVHSESSILSSLRGGDGGGIPCSKDELAINGSYTDPMGRRKSKRFKVAAESEFSPDFGSITRQLRSRRMQKEFTVETYETRNVSDVCVLSSQADVELIPG

EIVAERDSFKSVDCNDMSVGLTEGAESLGVNMQEPMKDRNMPENTSEQNMVEVHPPSISLPEEDMMGSVCRKSITGTKELHGRTISVGRDLSPNMGSKFS

KNGKTAKRSISVEEENLVLEKSDSGDHLGPSPEVLELEKSEVWIITDKGVVMPSPVKPSEKRNGDYGEGSMRKNSERVALDKKRLASKFRLSNGGLPSCS

SSGDSARYKVKETMRLFHETCKKIMQEEEARPRKRDGGNFKVVCEASKILKSKGKNLYSGTQIIGTVPGVEVGDEFQYRMELNLLGIHRPSQSGIDYMKD

DGGELVATSIVSSGGYNDVLDNSDVLIYTGQGGNVGKKKNNEPPKDQQLVTGNLALKNSINKKNPVRVIRGIKNTTLQSSVVAKNYVYDGLYLVEEYWEE

TGSHGKLVFKFKLRRIPGQPELPWKEVAKSKKSEFRDGLCNVDITEGKETLPICAVNNLDDEKPPPFIYTAKMIYPDWCRPIPPKSCGCTNGCSKSKNCA

CIVKNGGKIPYYDGAIVEIKPLVYECGPHCKCPPSCNMRVSQHGIKIKLEIFKTESRGWGVRSLESIPIGSFICEYAGELLEDKQAESLTGKDEYLFDLG

DEDDPFTINAAQKGNIGRFINHSCSPNLYAQDVLYDHEEIRIPHIMFFALDNIPPLQELSYDYNYKIDQVYDSNGNIKKKFCYCGSAECSGRLY

>AtASHH3_SDG7

MPASKKISDRNHLGQVFDKLLNQIGESEEFELPEWLNKGKPTPYIFIRRNIYLTKKVKRRVEDDGIFCSCSSSSPGSSSTVCGSNCHCGMLFSSCSSSCK

CGSECNNKPFQQRHVKKMKLIQTEKCGSGIVAEEEIEAGEFIIEYVGEVIDDKTCEERLWKMKHRGETNFYLCEITRDMVIDATHKGNKSRYINHSCNPN

TQMQKWIIDGETRIGIFATRGIKKGEHLTYDYQFVQFGADQDCHCGAVGCRRKLGVKPSKPKIASDEAFNLVAHELAQTLPKVHQNGLVNRHIDAGKSWN

NLSQRDTCSRNCIGVVIRLSRPTSDRCFGLVRHFDEYSRKHSVMFEDGVTEFVDMSREDWEIV

>AtSUVR3_SDG20

MQRLRESPPPKTRCLGEASDIIPAADRFLRCANLILPWLNPRELAVVAQTCKTLSLISKSLTIHRSLDAARSLENISIPFHNSIDSQRYAYFIYTPFQIP

ASSPPPPRQWWGAAANECGSESRPCFDSVSESGRFGVSLVDESGCECERCEEGYCKCLAFAGMEEIANECGSGCGCGSDCSNRVTQKGVSVSLKIVRDEK

KGWCLYADQLIKQGQFICEYAGELLTTDEARRRQNIYDKLRSTQSFASALLVVREHLPSGQACLRINIDATRIGNVARFINHSCDGGNLSTVLLRSSGAL

LPRLCFFAAKDIIAEEELSFSYGDVSVAGENRDDKLNCSCGSSCCLGTLPCENT

>AtSUVR4_SDG20

MISLSGLTSSVESDLDMQQAMLTNKDEKVLKALERTRQLDIPDEKTMPVLMKLLEEAGGNWSYIKLDNYTALVDAIYSVEDENKQSEGSSNGNRGKNLKV

IDSPATLKKTYETRSASSGSSIQVVQKQPQLSNGDRKRKYKSRIADITKGSESVKIPLVDDVGSEAVPKFTYIPHNIVYQSAYLHVSLARISDEDCCANC

KGNCLSADFPCTCARETSGEYAYTKEGLLKEKFLDTCLKMKKEPDSFPKVYCKDCPLERDHDKGTYGKCDGHLIRKFIKECWRKCGCDMQCGNRVVQRGI

RCQLQVYFTQEGKGWGLRTLQDLPKGTFICEYIGEILTNTELYDRNVRSSSERHTYPVTLDADWGSEKDLKDEEALCLDATICGNVARFINHRCEDANMI

DIPIEIETPDRHYYHIAFFTLRDVKAMDELTWDYMIDFNDKSHPVKAFRCCCGSESCRDRKIKGSQGKSIERRKIVSAKKQQGSKEVSKKRK

>AtATXR2_SDG36

MDSVYKTDENFAADVAALLAPLPTPQLQEYFNKLITSRRCNGIEVKNNGTIGKGVYANSEFDEDELILKDEILVGIQHSSNKVDCLVCSFCFRFIGSIEK

QIGRKLYFKNLGVSGCCDDDSSEEDECVKYNGNEEQCGGSSSSHNTLPEGVVSSLMNGEMALPHTDKFPLPSPLSCPGGCQEAFYCSESCAAADWESSHS

LLCTGERSESISREALGEFIKHANDTNDIFLLAAKAIAFTILRYRKLKAEHVDKKAKQSEPKQSLLLEAWKPVSIGYKRRWWDCIALPDDVDPTDEGAFR

MQIKNLACTSLELLKIAIFDKECEALFSLEIYGNIIGMFELNNLDLVVASPVEDYFLYIDDLPDAEKEETEEITRPFLDALGDEYSDCCQGTAFFPLQSC

MNHSCCPNAKAFKREEDRDGQAVIIALRRISKNEEVTISYIDEELPYKERQALLADYGFSCKCSKCLEDSSSI

>AtSDG49_At3g55080

MLFCISTVKLFGFQQRRNVSSLAKRFSLAGKLTLELQTQASLDNNFLPWLERIAGAKITNTLSIGKSTYGRSLFASKVIYAGDCMLKVPFNAQITPDELP

SDIRVLLSNEVGNIGMLAAVLIREKKMGQKSRWVPYISRLPQPAEMHSSIFWGEDELSMIRCSAVHQETVKQKAQIEKDFSFVAQAFKQHCPIVTERPDL

EDFMYAYALVGSRAWENSKRISLIPFADFMNHDGLSASIVLRDEDNQLSEVTADRNYSPGDEVFIKYGEFSNATLMLDFGFTFPYNIHDEVQIQMDVPND

DPLRNMKLGLLQTHHTRTVKDINIFHSSCDTFTIKEVKSAIGKGKGIPQSLRAFARVLCCIIPQELNDLSKEAAQNDGRLARLPFKDGNRELEAHKILLS

HINRLIEDHSVCIKEMEECYFVSQRFAVRRQMARDLLYGELRVLRSAAEWLNHYCTTLLSETM

>AtSDG50_At3g56570

MATRRLRAFKRWMQANGVDCSEALNLVDDENDGVSVRAFCDLKEGDVVANISKTACLTIKTSGAREMIESADLDGSLGLSVALMYERSLGEESPWAGYLQ

ILPIQEDLPLVWSLEDLDSLLSGTELHKLVKEDHVLIYEDWKENILPLTSSLPQNVDSDSFGIKEYLAAKSLIASRSFEIDDYHGSGMVPLADLFNHKTG

AEDVHFTHESDSEADESDNDDAANETTDEDEPSSKISSSPEQSFEEVPGENTDDEAKEEEEEEEEEEEGEEEEEGEEEEENSSMLQNDQSGLKMIMVKDV

SAGAEVFNTYGLMGNAALLHRYGFTELDNPYDIVNIDLELVTEWSTSSFTSRYTRARLALWRKLGYTGCESQNSEYFEVSSTGEPQTELLILLYILLLPD

DTYNKLDLAESTTGASPSKEGKRSSSYEITIGKHKFVYGESGNDILLTDGVCEALLTIVDKRESLYGSLSSLEDDIVRVKTCCLPRDRRLYHSLVLRVSE

RKILKKLRSYIHTQTNESSSGKRRKKMVPKS

>AtASHH4_SDG24_AT3G59960

MSSSKKGSDRNQIRKSLRKLKKQIGELEKLESPDRLNNVKPIFIKRNIYLKKKLKKKVKDHGIFCSCSLDPGSSTLCGSDCNCGILLSSCSSSCKCSSEC

TNKPFQQRHIKKMKLVQTEKCGYGIVADEDINSGEFIIEYVGEVIDDKICEERLWKLNHKVETNFYLCQINWNMVIDATHKGNKSRYINHSCSPNTEMQK

WIIDGETRIGIFATRFINKGEQLTYDYQFVQFGADQDCYCGAVCCRKKLGAKPCKTKNTTLEEAVKPVACKVTWKTPKLLNSEVRETNLDASGQAWNNHS

QRKICCRDCIGAYYTAQMKVLTLVVDIFQVMYEDGVTEIIDMCREVWKVVTA

>AtATX3_SDG14

MILKRTLTTFENQNLKRCKIDSEIEYGRKKGEIIVYKKRQRATVDQPCSKEPELLTSSSSSLTSKEESQQVCSDQSKSSRGRVRAVPSRFKDSIVGTWKS

SRRKGESTESSHDDDDVSLGKKVKGFSGSSKLHRSKDSKVFPRKDNGDSSEVDCDYWDVQISYDDANFGMPKKSDASRKGVYKPEEFTVGDLVWAKCGKR

FPAWPAVVIDPISQAPDGVLKHCVPGAICVMFFGYSKDGTQRDYAWVRQGMVYPFTEFMDKFQDQTNLFNYKASEFNKALEEAVLAENGNFGDAEIISPD

SSATESDQDYGPASRFQGSYHEDIRTCDGCGSVMPLKSLKRTKDSQPEELLCKHCSKLRKSNQYCGICKRIWHPSDDGDWVCCDGCDVWVHAECDNITNE

RFKELEHNNYYCPDCKVQHELTPTILEEQNSVFKSTEKTTETGLPDAITVVCNGMEGTYIRKFHAIECKCGSCGSRKQSPSEWERHTGCRAKKWKYSVRV

KDTMLPLEKWIAEFSTYTLETQMLDKQKMLSLLEEKYEPVRAKWTTERCAVCRWVEDWEENKMIICNRCQVAVHQECYGVSKSQDLTSWVCRACETPDIE

RDCCLCPVKGGALKPSDVEGLWVHVTCAWFRPEVGFLNHENMEPAVGLFKIPANSFLKVCTICKQTHGSCVHCCKCATHFHAMCASRAGYNMELHCLEKN

GVQRTRKSVYCSFHRKPDPDSVVVVHTPSGVFGSRNLLQNQYGRAKGSRLVLTKKMKLPGFQTQTQAEQSRVFDSLSAARCRIYSRSNTKIDLEAISHRL

KGPSHHSLSAIENLNSFKASFSFRAPFMSVFCFLGATFSEYLRKILISIYLVTHQEADFTSFRERLKHLQRTENFRVCFGKSGIHGWGLFARKSIQEGEM

IIEYRGVKVRRSVADLREANYRSQGKDCYLFKISEEIVIDATDSGNIARLINHSCMPNCYARIVSMGDGEDNRIVLIAKTNVAAGEELTYDYLFEVDESE

EIKVPCLCKAPNCRKFMN

>AtSWN_SDG10

MVTDDSNSSGRIKSHVDDDDDGEEEEDRLEGLENRLSELKRKIQGERVRSIKEKFEANRKKVDAHVSPFSSAASSRATAEDNGNSNMLSSRMRMPLCKLN

GFSHGVGDRDYVPTKDVISASVKLPIAERIPPYTTWIFLDRNQRMAEDQSVVGRRQIYYEQHGGETLICSDSEEEPEPEEEKREFSEGEDSIIWLIGQEY

GMGEEVQDALCQLLSVDASDILERYNELKLKDKQNTEEFSNSGFKLGISLEKGLGAALDSFDNLFCRRCLVFDCRLHGCSQPLISASEKQPYWSDYEGDR

KPCSKHCYLQLKAVREVPETCSNFASKAEEKASEEECSKAVSSDVPHAAASGVSLQVEKTDIGIKNVDSSSGVEQEHGIRGKREVPILKDSNDLPNLSNK

KQKTAASDTKMSFVNSVPSLDQALDSTKGDQGGTTDNKVNRDSEADAKEVGEPIPDNSVHDGGSSICQPHHGSGNGAIIIAEMSETSRPSTEWNPIEKDL

YLKGVEIFGRNSCLIARNLLSGLKTCLDVSNYMRENEVSVFRRSSTPNLLLDDGRTDPGNDNDEVPPRTRLFRRKGKTRKLKYSTKSAGHPSVWKRIAGG

KNQSCKQYTPCGCLSMCGKDCPCLTNETCCEKYCGCSKSCKNRFRGCHCAKSQCRSRQCPCFAAGRECDPDVCRNCWVSCGDGSLGEAPRRGEGQCGNMR

LLLRQQQRILLGKSDVAGWGAFLKNSVSKNEYLGEYTGELISHHEADKRGKIYDRANSSFLFDLNDQYVLDAQRKGDKLKFANHSAKPNCYAKVMFVAGD

HRVGIFANERIEASEELFYDYRYGPDQAPVWARKPEGSKKDDSAITHRRARKHQSH

>AtSUVH9_SDG22

MGSSHIPLDPSLNPSPSLIPKLEPVTESTQNLAFQLPNTNPQALISSAVSDFNEATDFSSDYNTVAESARSAFAQRLQRHDDVAVLDSLTGAIVPVEENP

EPEPNPYSTSDSSPSVATQRPRPQPRSSELVRITDVGPESERQFREHVRKTRMIYDSLRMFLMMEEAKRNGVGGRRARADGKAGKAGSMMRDCMLWMNRD

KRIVGSIPGVQVGDIFFFRFELCVMGLHGHPQSGIDFLTGSLSSNGEPIATSVIVSGGYEDDDDQGDVIMYTGQGGQDRLGRQAEHQRLEGGNLAMERSM

YYGIEVRVIRGLKYENEVSSRVYVYDGLFRIVDSWFDVGKSGFGVFKYRLERIEGQAEMGSSVLKFARTLKTNPLSVRPRGYINFDISNGKENVPVYLFN

DIDSDQEPLYYEYLAQTSFPPGLFVQQSGNASGCDCVNGCGSGCLCEAKNSGEIAYDYNGTLIRQKPLIHECGSACQCPPSCRNRVTQKGLRNRLEVFRS

LETGWGVRSLDVLHAGAFICEYAGVALTREQANILTMNGDTLVYPARFSSARWEDWGDLSQVLADFERPSYPDIPPVDFAMDVSKMRNVACYISHSTDPN

VIVQFVLHDHNSLMFPRVMLFAAENIPPMTELSLDYGVVDDWNAKLAICN

>AtATXR3_SDG2

MSDGGVACMPLLNIMEKLPIVEKTTLCGGNESKTAATTENGHTSIATKVPESQPANKPSASSQPVKKKRIVKVIRKVVKRRPKQPQKQADEQLKDQPPSQ

VVQLPAESQLQIKEQDKKSEFKGGTSGVKEVENGGDSGFKDEVEEGELGTLKLHEDLENGEISPVKSLQKSEIEKGEIVGESWKKDEPTKGEFSHLKYHK

GYVERRDFSADKNWKGGKEEREFRSWRDPSDEIEKGEFIPDRWQKMDTGKDDHSYIRSRRNGVDREKTWKYEYEYERTPPGGRFVNEDIYHQREFRSGLD

RTTRISSKIVIEENLHKNEYNNSSNFVKEYSSTGNRLKRHGAEPDSIERKHSYADYGDYGSSKCRKLSDDCSRSLHSDHYSQHSAERLYRDSYPSKNSSL

EKYPRKHQDASFPAKAFSDKHGHSPSRSDWSPHDRSRYHENRDRSPYARERSPYIFEKSSHARKRSPRDRRHHDYRRSPSYSEWSPHDRSRPSDRRDYIP

NFMEDTQSDRNRRNGHREISRKSGVRERRDCQTGTELEIKHKYKESNGKESTSSSKELQGKNILYNNSLLVEKNSVCDSSKIPVPCATGKEPVQVGEAPT

EELPSMEVDMDICDTPPHEPMASDSSLGKWFYLDYYGTEHGPARLSDLKALMEQGILFSDHMIKHSDNNRWLVNPPEAPGNLLEDIADTTEAVCIEQGAG

DSLPELVSVRTLPDGKEIFVENREDFQIDMRVENLLDGRTITPGREFETLGEALKVNVEFEETRRCVTSEGVVGMFRPMKRAIEEFKSDDAYGSESDEIG

SWFSGRWSCKGGDWIRQDEASQDRYYKKKIVLNDGFPLCLMQKSGHEDPRWHHKDDLYYPLSSSRLELPLWAFSVVDERNQTRGVKASLLSVVRLNSLVV

NDQVPPIPDPRAKVRSKERCPSRPARPSPASSDSKRESVESHSQSTASTGQDSQGLWKTDTSVNTPRDRLCTVDDLQLHIGDWFYTDGAGQEQGPLSFSE

LQKLVEKGFIKSHSSVFRKSDKIWVPVTSITKSPETIAMLRGKTPALPSACQGLVVSETQDFKYSEMDTSLNSFHGVHPQFLGYFRGKLHQLVMKTFKSR

DFSAAINDVVDSWIHARQPKKESEKYMYQSSELNSCYTKRARLMAGESGEDSEMEDTQMFQKDELTFEDLCGDLTFNIEGNRSAGTVGIYWGLLDGHALA

RVFHMLRYDVKSLAFASMTCRHWKATINSYKDISRQVDLSSLGPSCTDSRLRSIMNTYNKEKIDSIILVGCTNVTASMLEEILRLHPRISSVDITGCSQF

GDLTVNYKNVSWLRCQNTRSGELHSRIRSLKQTTDVAKSKGLGGDTDDFGNLKDYFDRVEKRDSANQLFRRSLYKRSKLYDARRSSAILSRDARIRRWAI

KKSEHGYKRVEEFLASSLRGIMKQNTFDFFALKVSQIEEKMKNGYYVSHGLRSVKEDISRMCREAIKDELMKSWQDGSGLSSATKYNKKLSKTVAEKKYM

SRTSDTFGVNGASDYGEYASDREIKRRLSKLNRKSFSSESDTSSELSDNGKSDNYSSASASESESDIRSEGRSQDLRIEKYFTADDSFDSVTEEREWGAR

MTKASLVPPVTRKYEVIEKYAIVADEEEVQRKMRVSLPEDYGEKLNAQRNGIEELDMELPEVKEYKPRKLLGDEVLEQEVYGIDPYTHNLLLDSMPGELD

WSLQDKHSFIEDVVLRTLNRQVRLFTGSGSTPMVFPLRPVIEELKESAREECDIRTMKMCQGVLKEIESRSDDKYVSYRKGLGVVCNKEGGFGEEDFVVE

FLGEVYPVWKWFEKQDGIRSLQENKTDPAPEFYNIYLERPKGDADGYDLVVVDAMHMANYASRICHSCRPNCEAKVTAVDGHYQIGIYSVRAIEYGEEIT

FDYNSVTESKEEYEASVCLCGSQVCRGSYLNLTGEGAFQKVLKDWHGLLERHRLMLEACVLNSVSEEDYLELGRAGLGSCLLGGLPDWMIAYSARLVRFI

NFERTKLPEEILKHNLEEKRKYFSDIHLDVEKSDAEVQAEGVYNQRLQNLAVTLDKVRYVMRHVFGDPKNAPPPLERLTPEETVSFVWNGDGSLVDELLQ

SLSPHLEEGPLNELRSKIHGHDPSGSADVLKELQRSLLWLRDEIRDLPCTYKCRNDAAADLIHIYAYTKCFFKVREYQSFISSPVHISPLDLGAKYADKL

GESIKEYRKTYGENYCLGQLIYWYNQTNTDPDLTLVKATRGCLSLPDVASFYAKAQKPSKHRVYGPKTVKTMVSQMSKQPQRPWPKDKIWTFKSTPRVFG

SPMFDAVLNNSSSLDRELLQWLRNRRHVFQATWDS

>AtATX4_SDG16_AT4G27910

MIIKRKFKTQIPSLERCKLGNESRKKKRKLNLGGGGYYYPLNLLGEIAAGIVPGNGRNGFSASWCTEVTKPVEVEESLSKRRSDSGTVRDSPPAEVSRPP

LVRTSRGRIQVLPSRFNDSVLDNWRKDSKSDCDLEEEEIECRNEKVVSFRVPKATNLKSKELDRKSKYSALCKEERFHEQHNDEARARVDEKLPNKKGTF

GPENFYSGDLVWAKSGRNEPFWPAIVIDPMTQAPELVLRSCIPDAACVVFFGHSGNENERDYAWVRRGMIFPFVDYVARFQEQPELQGCKPGNFQMALEE

AFLADQGFTEKLMHDIHLAAGNSTFDDSFYRWIQETAVSNQELNNNAPRQGLLKKHRNPLACAGCETVISFEMAKKMKDLIPGDQLLCKPCSRLTKSKHI

CGICKKIRNHLDNKSWVRCDGCKVRIHAECDQISDRHLKDLRETDYYCPTCRAKFNFDLSDSEKQNSKSKVAKGDGQMVLPDKVIVVCAGVEGVYFPRLH

LVVCKCGSCGPKKKALSEWERHTGSKSKNWKTSVKVKSSKLALEDWMMNLAELHANATAAKVPKRPSIKQRKQRLLAFLSETYEPVNAKWTTERCAVCRW

VEDWDYNKIIICNRCQIAVHQECYGARHVRDFTSWVCKACERPDIKRECCLCPVKGGALKPTDVETLWVHVTCAWFQPEVCFASEEKMEPAVGILSIPST

NFVKICVICKQIHGSCTQCCKCSTYYHAMCASRAGYRMELHCLEKNGQQITKMVSYCAYHRAPNPDNVLIIQTPSGAFSAKSLVQNKKKGGSRLISLIRE

DDEAPAENTITCDPFSAARCRVFKRKINSKKRIEEEAIPHHTRGPRHHASAAIQTLNTFRHVPEEPKSFSSFRERLHHLQRTEMDRVCFGRSGIHGWGLF

ARRNIQEGEMVLEYRGEQVRGSIADLREARYRRVGKDCYLFKISEEVVVDATDKGNIARLINHSCTPNCYARIMSVGDEESRIVLIAKANVAVGEELTYD

YLFDPDEAEELKVPCLCKAPNCRKFMN

>AtASHR3_SDG4_AT4G30860

MLDLGNMSMSASVALTCCPSFLPAASGPELAKSINSPENLAGECNGKHLPMIPPEEEVKDIKIANGVTAFTRKQNPSDRVKKGFVLDDHVKDWVKRRVAS

GVSESTCFLPFLVGAKKMVDCLVCHKPVYPGEDLSCSVRGCQGAYHSLCAKESLGFSKSSKFKCPQHECFVCKQRTQWRCVKCPMAAHDKHSPWSKEILH

LKDQPGRAVCWRHPTDWRLDTKHAVAQSEIEEVFCQLPLPYVEEEFKIDLAWKDSVVKEDPPSYVHIRRNIYLVKKKRDNANDGVGCTNCGPNCDRSCVC

RVQCISCSKGCSCPESCGNRPFRKEKKIKIVKTEHCGWGVEAAESINKEDFIVEYIGEVISDAQCEQRLWDMKHKGMKDFYMCEIQKDFTIDATFKGNAS

RFLNHSCNPNCVLEKWQVEGETRVGVFAARQIEAGEPLTYDYRFVQFGPEVKCNCGSENCQGYLGTKRKEPNCLVVSWGAKRRRLFHRPIARKPQQD

>AtSUVH1_SDG32_AT5G04940

MERNGGHYTDKTRVLDIKPLRTLRPVFPSGNQAPPFVCAPPFGPFPPGFSSFYPFSSSQANQHTPDLNQAQYPPQHQQPQNPPPVYQQQPPQHASEPSLV

TPLRSFRSPDVSNGNAELEGSTVKRRIPKKRPISRPENMNFESGINVADRENGNRELVLSVLMRFDALRRRFAQLEDAKEAVSGIIKRPDLKSGSTCMGR

GVRTNTKKRPGIVPGVEIGDVFFFRFEMCLVGLHSPSMAGIDYLVVKGETEEEPIATSIVSSGYYDNDEGNPDVLIYTGQGGNADKDKQSSDQKLERGNL

ALEKSLRRDSAVRVIRGLKEASHNAKIYIYDGLYEIKESWVEKGKSGHNTFKYKLVRAPGQPPAFASWTAIQKWKTGVPSRQGLILPDMTSGVESIPVSL

VNEVDTDNGPAYFTYSTTVKYSESFKLMQPSFGCDCANLCKPGNLDCHCIRKNGGDFPYTGNGILVSRKPMIYECSPSCPCSTCKNKVTQMGVKVRLEVF

KTANRGWGLRSWDAIRAGSFICIYVGEAKDKSKVQQTMANDDYTFDTTNVYNPFKWNYEPGLADEDACEEMSEESEIPLPLIISAKNVGNVARFMNHSCS

PNVFWQPVSYENNSQLFVHVAFFAISHIPPMTELTYDYGVSRPSGTQNGNPLYGKRKCFCGSAYCRGSFG

>AtATXR4_SDG38_AT5G06620

MSRLALNRYSRCFSRLKTLTTPLFFSSSAASNRDGDYQIGPPPIRVGLTESAGRAVFATRKIGAGDLIHTAKPVVACPSLLKLDSVCYLCLKKLMGSAKF

EDRGVSYCSQECQENSKGFLDVETRADWSSFDDYCRTHNFKYPLMVKRLCCMIISGARPADCLDILQPAVLSSEMISKIEDGYGLLWNAFRKANFKDDDV

AFLTKQWYTAILARIRINAFRIDLVGGSCGEDLLSLAAASVEGEGAVGHAVYMLPSFYNHDCDPNAHIIWLHNADARLNTLRDVEEGEELRICYIDASMG

YEARQTILSQGFGFLCNCLRCQSTD

>AtATXR5_SDG15_AT5G09790

MATWNASSPAASPCSSRRRTKAPARRPSSESPPPRKMKSMAEIMAKSVPVVEQEEEEDEDSYSNVTCEKCGSGEGDDELLLCDKCDRGFHMKCLRPIVVR

VPIGTWLCVDCSDQRPVRRLSQKKILHFFRIEKHTHQTDKLELSQEETRKRRRSCSLTVKKRRRKLLPLVPSEDPDQRLAQMGTLASALTALGIKYSDGL

NYVPGMAPRSANQSKLEKGGMQVLCKEDLETLEQCQSMYRRGECPPLVVVFDPLEGYTVEADGPIKDLTFIAEYTGDVDYLKNREKDDCDSIMTLLLSED

PSKTLVICPDKFGNISRFINGINNHNPVAKKKQNCKCVRYSINGECRVLLVATRDISKGERLYYDYNGYEHEYPTHHFL

>AtSUVH4_SDG33_KYP_AT5G13960

MAGKRKRANAPDQTERRSSVRVQKVRQKALDEKARLVQERVKLLSDRKSEICVDDTELHEKEEENVDGSPKRRSPPKLTAMQKGKQKLSVSLNGKDVNLE

PHLKVTKCLRLFNKQYLLCVQAKLSRPDLKGVTEMIKAKAILYPRKIIGDLPGIDVGHRFFSRAEMCAVGFHNHWLNGIDYMSMEYEKEYSNYKLPLAVS

IVMSGQYEDDLDNADTVTYTGQGGHNLTGNKRQIKDQLLERGNLALKHCCEYNVPVRVTRGHNCKSSYTKRVYTYDGLYKVEKFWAQKGVSGFTVYKYRL

KRLEGQPELTTDQVNFVAGRIPTSTSEIEGLVCEDISGGLEFKGIPATNRVDDSPVSPTSGFTYIKSLIIEPNVIIPKSSTGCNCRGSCTDSKKCACAKL

NGGNFPYVDLNDGRLIESRDVVFECGPHCGCGPKCVNRTSQKRLRFNLEVFRSAKKGWAVRSWEYIPAGSPVCEYIGVVRRTADVDTISDNEYIFEIDCQ

QTMQGLGGRQRRLRDVAVPMNNGVSQSSEDENAPEFCIDAGSTGNFARFINHSCEPNLFVQCVLSSHQDIRLARVVLFAADNISPMQELTYDYGYALDSV

HGPDGKVKQLACYCGALNCRKRLY

>AtSDG40_AT5g17240

MDLEHQTMETFLRWAAEIGISDSIDSSRFRDSCLGHSLSVSDFPDAGGRGLGAARELKKGELVLKVPRKALMTTESIIAKDLKLSDAVNLHNSLSSTQIL

SVCLLYEMSKEKKSFWYPYLFHIPRDYDLLATFGNFEKQALQVEDAVWATEKATAKCQSEWKEAGSLMKELELKPKFRSFQAWLWASATISSRTLHVPWD

SAGCLCPVGDLFNYDAPGDYSNTPQGPESANNVEEAGLVVETHSERLTDGGFEEDVNAYCLYARRNYQLGEQVLLCYGTYTNLELLEHYGFMLEENSNDK

VFIPLETSLFSLASSWPKDSLYIHQDGKLSFALISTLRLWLIPQSQRDKSVMRLVYAGSQISVKNEILVMKWMSEKCGSVLRDLPTSVTEDTVLLHNIDK

LQDPELRLEQKETEAFGSEVRAFLDANCLWDVTVLSGKPIEFSRKTSRMLSKWRWSVQWRLSYKRTLADCISYCNEKMNNLLGTQDRLRDL

>AtATXR6_SDG34_AT5G24330

MVAVRRRRTQASNPRSEPPQHMSDHDSDSDWDTVCEECSSGKQPAKLLLCDKCDKGFHLFCLRPILVSVPKGSWFCPSCSKHQIPKSFPLIQTKIIDFFR

IKRSPDSSQISSSSDSIGKKRKKTSLVMSKKKRRLLPYNPSNDPQRRLEQMASLATALRASNTKFSNELTYVSGKAPRSANQAAFEKGGMQVLSKEGVET

LALCKKMMDLGECPPLMVVFDPYEGFTVEADRFIKDWTIITEYVGDVDYLSNREDDYDGDSMMTLLHASDPSQCLVICPDRRSNIARFISGINNHSPEGR

KKQNLKCVRFNINGEARVLLVANRDISKGERLYYDYNGYEHEYPTEHFV

>AtATXR7_SDG25_AT5G42400

MVAVDSTFPSHGSSYSSRRKKVSALEPNYFGSMCMGVYSDDVSISAREVAQDYSCDSCGDLATVSSACCNFDELCGLDSALEMGCRSNEDCRAGQEASGS

GIASGLDKSVPGYTMYASGWMYGNQQGQMCGPYTQQQLYDGLSTNFLPEDLLVYPIINGYTANSVPLKYFKQFPDHVATGFAYLQNGIISVAPSVTSFPP

SSSNATVHQDEIQTEHATSATHLISHQTMPPQTSSNGSVLDQLTLNHEESNMLASFLSLGNEHACWFLVDGEGRNHGPHSILELFSWQQHGYVSDAALIR

DGENKLRPITLASLIGVWRVKCGDANCDEPVTGVNFISEVSEELSVHLQSGIMKIARRALLDEIISSVISDFLKAKKSDEHLKSYPPTSAVESISSRVIN

AEKSVVSNTESAGCKNTMNEGGHSSIAAESSKYTKSVGSIENFQTSCSAVCRTLHHHCMQIMWNAVFYDTVATHSSCWRKNKIWFRSSDISTVNYCKGSH

TKYSDKPESFESFTCRVDSSSSKTAYSDEFDLATNGARVRGLSSDTYGTESVIASISEHVENELFLSLKTHLTDYTSILIKDGANNTTSSARDGKMHEGS

FREQYNLEGSSKKKNGLNVVPAKLRFSNDFSDSQRLLQEGESSEQITSEDIIANIFSTALETSDIPVNDELDALAIHEPPPPGCESNINMPCLRYKYQPV

RSKESIPEIKAYVSMALCRQKLHNDVMRDWKSLFLKCYLNEFLASLKGSHQVSRKETLALKKRKTVTRNKKLVQSNISNQTAEKLRKPCVGASEKVLVKR

SKKLSDSHSMKEVLKVDTPSIDLSVRKPSQQKMRNTDRRDHCIIKDATKLHKEKVGKDAFSKVICDKSQDLEMEDEFDDALLITRLRRISRNKTKELREC

RNAAKSCEEISVTAEESEETVDCKDHEESLSNKPSQKVKKAHTSKLKRKNLSDARDEGTKSCNGAVKSFTEISGKEGDTESLGLAISDKVSHQNLSKRRK

SKIALFLFPGFENTSRKCFTKLLSPEDAAKNGQDMSNPTGNPPRLAEGKKFVEKSACSISQKGRKSSQSSILKRKHQLDEKISNVPSRRRLSLSSTDSED

AVIKEDYDVRNEEKLPCHTSDKLQKGPNKLIRRRKPLAKHTTERSPIKDLSVDDGRPKPIALKPLEKLSSKPSKKKLFLSIPKSDGCARTSINGWHWHAW

SLKASAEERARVRGSSCVHMQHFGSKSSLTQNVLSARTNRAKLRNLLAAADGADVLKMSQLKARKKHLRFQQSKIHDWGLVALEPIEAEDFVIEYVGELI

RSSISEIRERQYEKMGIGSSYLFRLDDGYVLDATKRGGIARFINHSCEPNCYTKIISVEGKKKIFIYAKRHIDAGEEISYNYKFPLEDDKIPCNCGAPNV

YCFCEQVPWIAKLKRRTWFSRRN

>AtSUVR2_SDG18_AT5G43990

MRSYNLFHESNPTISFSFVGAETMAPNLHIKKAFMAMRAMGIEDARVKPVLKNLLALYEKNWELIAEDNYRVLADAIFDSHEDQAIQESEEKKADEVKED

EGCAAEVDRGKKKLHESIEDDEDVMAESDRPLKRLRRRGEGGSALASPSLGSPTLEGPSINDEENAPILLPYHPVPIENDHDAGELILTKVEPITNMPLS

SIPDSVDRGDSSMLEIDKSNGHVEEKAGETVSTADGTTNDISPTTVARFSDHKLAATIEEPPALELASSASGEVKINLSFAPATGGSNPHLPSMEELRRA

MEEKCLRSYKILDPNFSVLGFMNDICSCYLDLATNGRDSANQLPKNLPFVTTNIDALKKSAARMAYTSQASNDVVEICSNEHMRDAENGAVGDSMALVVV

PECQLSADEWRLISSVGDISLGKETVEIPWVNEVNDKVPPVFHYIAQSLVYQDAAVKFSLGNIRDDQCCSSCCGDCLAPSMACRCATAFNGFAYTVDGLL

QEDFLEQCISEARDPRKQMLLYCKECPLEKAKKEVILEPCKGHLKRKAIKECWSKCGCMKNCGNRVVQQGIHNKLQVFFTPNGRGWGLRTLEKLPKGAFV

CELAGEILTIPELFQRISDRPTSPVILDAYWGSEDISGDDKALSLEGTHYGNISRFINHRCLDANLIEIPVHAETTDSHYYHLAFFTTREIDAMEELTWD

YGVPFNQDVFPTSPFHCQCGSDFCRVRKQISKGKNVKKRA

>AtATX5_SDG29_AT5G53430

MIIKRKLKTLKRCNSTNEEDDIVRKKRKVNLNGGGSGGDYYYPLNLLGEIGAGIVPGKNGFSVSLCKQVSCSPKVEVVEEEEEEEEIKSTRLVSRPPLVK

TSRGRVQVLPSRFNDSVIENWRKDNKSSGEEREEEIEEEACRKEKVKVSSNHSLKIKQQETKFTPRNYKYSSSSALCGEIDDEDKCEEIVRYGNSFEMKK

QRYVDDEPRPKKEGVYGPEDFYSGDLVWGKSGRNEPFWPAIVIDPMTQAPELVLRSCIPDAACVMFFGHSGTENERDYAWVRRGMIFPFVDYVERLQEQS

ELRGCNPRDFQMALEEALLADQGFTEKLMQDIHMAAGNQTFDDSVYRWVEEAAGSSQYLDHVAPSQDMKKYRNPRACVGCGMVLSFKMAQKMKALIPGDQ

LLCQPCSKLTKPKHVCGICKRIWNHLDSQSWVRCDGCKVWIHSACDQISHKHFKDLGETDYYCPTCRTKFDFELSDSEKPDSKSKLGKNNAPMVLPDKVI

VVCSGVEGIYFPSLHLVVCKCGSCGPERKALSEWERHTGSKAKNWRTSVKVKSSKLPLEEWMMKLAEFHANATAAKPPKRPSIKQRKQRLLSFLREKYEP

VNVKWTTERCAVCRWVEDWDYNKIIICNRCQIAVHQECYGTRNVRDFTSWVCKACETPEIKRECCLCPVKGGALKPTDVETLWVHVTCAWFQPEVCFASE

EKMEPALGILSIPSSNFVKICVICKQIHGSCTQCCKCSTYYHAMCASRAGYRMELHCLEKNGRQITKMVSYCSYHRAPNPDTVLIIQTPSGVFSAKSLVQ

NKKKSGTRLILANREEIEESAAEDTIPIDPFSSARCRLYKRTVNSKKRTKEEGIPHYTGGLRHHPSAAIQTLNAFRHVAEEPKSFSSFRERLHHLQRTEM

ERVCFGRSGIHGWGLFARRNIQEGEMVLEYRGEQVRGIIADLREARYRREGKDCYLFKISEEVVVDATEKGNIARLINHSCMPNCYARIMSVGDDESRIV

LIAKTTVASCEELTYDYLFDPDEPDEFKVPCLCKSPNCRKFMN

>AtSDG45_At1g14030

MSASVAVVSGFLRIPSIQKSQNPSFLFSRPKKSLVRPISASSSELPENVRNFWKWLRDQGVVSGKSVAEPAVVPEGLGLVARRDIGRNEVVLEIPKRLWI

NPETVTASKIGPLCGGLKPWVSVALFLIREKYEEESSWRVYLDMLPQSTDSTVFWSEEELAELKGTQLLSTTLGVKEYVENEFLKLEQEILLPNKDLFSS

RITLDDFIWAFGILKSRAFSRLRGQNLVLIPLADLINHNPAIKTEDYAYEIKGAGLFSRDLLFSLKSPVYVKAGEQVYIQYDLNKSNAELALDYGFVESN

PKRNSYTLTIEIPESDPFFGDKLDIAESNKMGETGYFDIVDGQTLPAGMLQYLRLVALGGPDAFLLESIFNNTIWGHLELPVSRTNEELICRVVRDACKS

ALSGFDTTIEEDEKLLDKGKLEPRLEMALKIRIGEKRVLQQIDQIFKDRELELDILEYYQERRLKDLGLVGEQGDIIFWETK

>AtSDG46_At1g24610

MANSKMAIASLAAQIRPFTCLAASLPSRLAPHPPDLIRWIKREGGFVHHAVKLSQETQFGIGLISTEQISPGTDLISLPPHVPLRFESDDSSSSSSSLLS

ALARRVPEELWAMKLGLRLLQERANADSFWWPYISNLPETYTVPIFFPGEDIKNLQYAPLLHQVNKRCRFLLEFEQEIRRTLEDVKASDHPFSGQDVNAS

ALGWTMSAVSTRAFRLHGNKKLQGGSSDDVPMMLPLIDMCNHSFKPNARIIQEQNGADSNTLVKVVAETEVKENDPLLLNYGCLSNDFFLLDYGFVIESN

PYDTIELKYDEQLMDAASMAAGVSSPKFSSPAPWQHQLLSQLNLAGEMPNLKVTIGGPEPVEGRLLAALRILLCGELVEVEKHDSDTLKSLSAVAPFGIA

NEIAVFRTVIALCVIALSHFPTKIMEDEAIIKQGVSATAELSIKYRIQKKSVIIDVMKDLTRRVKLLSSKETPTAA

>AtSDG42_TPR9

MEKLKSLIPEDLLQTVKSSSVDDLLSTSSSLLRLFLGLPQFHQAVSELANPELGCCGKNEETSLDLKRRGNHCFRSRDFDEALRLYSKALRVAPLDAIDG

DKSLLASLFLNRANVLHNLGLLKESLRDCHRALRIDPYYAKAWYRRGKLNTLLGNYKDAFRDITVSMSLESSLVGKKQLQNELKAIPDYQNNQTLEHDEY

RPSNDAGVDHLPSVQMEVKLRCVSTKEKGRGMVSECDIEEASVIHVEEPFSVVISKSCRETHCHFCLNELPADTVPCPSCSIPVYCSESCQIQSGGMLST

NEMDKHHIFQKLPDDIVEHIKGVTSADIYYFATDLIQEHQHECRGANWPAVLPSDAVLAGRIIMKLINQGKAATDLSNLQEILELSHTYSKMNPENKLEL

HLLSIVLIWCLSKSSCPNLSVCEASVTQTIILLSQIKVNSIAVARMKSSGDSFKCLPSGNISTKEPIQSLEQIRVGQALYKTGSLFNHSCKPNIHLYFLS

RGLIMQTTEFVPTGCPLELSYGPEVGKWDCKNRIRFLEEEYFFHCRCRGCAQINISDLVINGYGCVNTNCTGVVLDSNVATCESEKLNHFFTAPRNVDQQ

VQMREKVYADVGEVASSLLSKPSGSLHIEPEICLKCGSRCDIENSHAEVNKAWNHMRRVEELMNSGRANYSVLSDCSRSIAVLRTFLHMYNKDIADAEDK

VAQACYLAGELVDARKHCEASIKILKRLYEDEHVVIGNEMVKLASIQLASGDSSGAWDTTKRSSQIFSKYYGSHAETLFSYLPCLKQETAKAVNLSTS

>AtSDG48_At3g07670

MAKACLLQSTLLPAYSPLHKLRNQNITLSFSPLPLSRCRPGIHCSVSAGETTIQSMEEAPKISWGCEIDSLENATSLQNWLSDSGLPPQKMAIDRVDIGE

RGLVASQNLRKGEKLLFVPPSLVISADSEWTNAEAGEVMKRYDVPDWPLLATYLISEASLQKSSRWFNYISALPRQPYSLLYWTRTELDMYLEASQIRER

AIERITNVVGTYEDLRSRIFSKHPQLFPKEVFNDETFKWSFGILFSRLVRLPSMDGRFALVPWADMLNHNCEVETFLDYDKSSKGVVFTTDRPYQPGEQV

FISYGNKSNGELLLSYGFVPREGTNPSDSVELALSLRKNDKCYEEKLDALKKHGLSTPQCFPVRITGWPMELMAYAYLVVSPPDMRNNFEEMAKAASNKT

STKNDLKYPEIEEDALQFILDSCETSISKYSRFLKESGSMDLDITSPKQLNRKAFLKQLAVDLSTSERRILYRAQYILRRRLRDIRSGELKALRLFSGLR

NFFK

>AtSDG43_pTAC14

MASSVSLQFLTNTFISKPQGFCNGIVSAPRPRSNLLRDRQNGVRPIKVASIETQPFPLFQSPASEESSSSELETADPDFYKIGYVRSVRAYGVEFKEGPD

GFGVYASKDIEPRRRARVIMEIPLELMITIRQKHPWMFFPDIVPIGHPIFDIINSTDPEIDWDIRLACLLLFSFDRDDHFWRLYGDFLPAADECSSLLLA

TEEDLAELQDPDLVSTIRQQQKRILDFWEKNWHSGVPLKIKRLAEDPERFIWAVSMAQTRCISMQTRVGALVQELNMMIPYADMLNHSFEPNCFLHWRPK

DRMLEVMSNAGQDIKKGEEMTINYMPGQKNNMLMERYGFSTPVNPWDAIKFSGDSRIHLNSFLSVFNIYGLPEEYYHDSELSRGDTFVDGAVIAAARTLP

TWSDIDLPPIPSAERKAVKELQDECRKMLAEYPTTAEQDQKLLDSMSEARTTFATAVKYRMHRKMFIGKIIKALDIYQERLLY

>AtSDG51_At5g14260

MEGVITCFHTKCVSLPIRSFPLSRVSSLPRWRNNKLISSSRSVHLRSLCVSSSDTLVASGSPKEDERQSKVSSKKEGDDSEDLKFWMDKNGLPPCKVILK

ERPAHDQKHKPIHYVAASEDLQKGDVAFSVPDSLVVTLERVLGNETIAELLTTNKLSELACLALYLMYEKKQGKKSVWYPYIRELDRQRGRGQLDAESPL

LWSEAELDYLTGSPTKAEVLERAEGIKREYNELDTVWFMAGSLFQQYPFDIPTEAFSFEIFKQAFVAIQSCVVHLQNVGLARRFALVPLGPPLLAYCSNC

KAMLTAVDGAVELVVDRPYKAGDPIVVWCGPQPNAKLLLNYGFVDEDNPYDRVIVEAALNTEDPQYQDKRMVAQRNGKLSQQVFQVRVGKEREAVQDMLP

YLRLGYMSDPSEMQSVISSQGPVCPMSPCMERAVLDQLANYFMRRLSGYPTTPKEDDALLADPSLSPRKRVATRLVQLEKKILVACLTTTVDLLNQLPDT

AISPCPAPYAPSLK

>OsSET1_SDG721

MLASRIPLKRCTAAAVVPLPGELEMEEGPTPPAGGGEGSGAAVVPAKRRRERVVPSRFRD

SVVSLPLPPAKKGRPAKKAAAREGGDGEVYDVEVRAVEQQGATAAAFGAVETAVWPGDER

PAQTEEELYRACRNIRRSSSSSGFSGSVVTSLSNAGGSVAPEGKPVVVVECKPKREGGDK

KEDFYWPEDFVLGDVVWARSGKKCPAWPAVVIDPLLHAPAVVLNSCIPGALCVMFFGFSS

GGHGRDYGWIKQGMIFPFVDYLDRFQGQALYKLKANRFRQAIEEAFLAERGFCELEMDEG

CSLEKSVNDQSVPDGLQEGSGSNNDQECQSEAQVVGKSTGCCDSCGNRVPPKIAKKKKQA

GEQLLCRHCDKLLQSKQYCGICKKIWHHTDGGNWVCCDECQIWVHVECDLTCINMEDLEN

ADYFCPDCKSKRKTVPPVEQMNTPNSSECASTSKEKLPEMIPVFCFGMDGMYLPKKHMIL

CQCNSCKERLMSLSEWERHTGSRKKNWKMSVKLKSNGDPLVTLLDDIPCANVKSSTPSIN

KEELLKLLANSFRPVNARWTTERCAVCRWVEDWDYNKIIICNRCQIAVHQECYGARDVQD

FTNWVCRACELPKQKRECCLCPVKGGALKPTDIDQLWVHVTCAWFQPKVSFPVDETMEPA

MGILSIPSEYFKKACVICKQMHGACTQCYKCSTYYHAMCASRAGYRMELQYSEKNGRNIT

RMVSYCAFHSTPDPDNVLIVKTPEGVFSTKFLPQNNEKQSGTRLVRKENLQEKVLPAKIS

DCPAARCLPYEMLKNKKEPGEAIAHRIMGPRHHSQESIEGLNACMDQKDEKSFATFRERL

RYLQKIENKRVSCGRSGIHGWGLFAAKKIQEGQMVIEYRGDQVRRSVADLREARYHREKK

DCYLFKISEDVVVDATEKGNIARLINHSCMPNCYARIMSVGDEKSQIILIAKRDVSAGEE

LTYDYLFDPDESEDCRVPCLCKALNCRGYMN*

>OsSET2_SDG705

MIFKRNQRSEILSLRRCNAGGGAGVGEDDGDGGERRPKRRRGDEFFPVELLGDVPVAGIP

YAAFGFRWCEEAEVASPAAASRAAAAAAAASRPPVVRTSRGRAQVLPSRFNDSVLIDPWK

KDKPAKPPVPAKAVQLVPKSEVLYRKGAIADRSLTMSELDENGDDDYEEGHNFVASRKYS

MSLSTVTSVHGEPYSYYHRKGLMKRQYDDDDDDDDDDDDDDDDEDEEAEEEEEEEEEEEE

ELSYWRNDFVYGDIVWARLGKRQPVWPGVVVDPAQPAAAQALPPQPRSGAVLCVMLFGWA

AEFGDEKKFIWVRQGGIFPFVDYMDRFQGQTELSSCKPGDFQRALEEAFLADQGFFEVPM

DGNTTGQPAVCQSFPADLEEVTGSNELECQSQIKRYKRALQCESCGNCFPNKDPSMMVYV

MEQLACRQCAKILRSKEYCGVCLKSWQHKCGGRWVCCHGCESWVHAECDKKCSNLKDLRD

NSYFCPYCRVKQNSNLSSKKTKSYEHRTDNSTQKSSKPDKVAVICFGMEGTYLRDLELIS

CHCGPCKGQKFLFNEWERHAGCRSKNWKSSIKIKDTLMPFGKWIEQHQSSSYSTNPAKRS

SQKMKKQKLLDLLSEPYDTVNVKWTTERCAVCRWVEDWDYNKIVICNRCQIAVHQECYGV

RGKQDFTSWPQVAFASDELMEPAIGILNITPLLFMKMCVICRQIHGSCTQCYRCSTYYHA

ICASRAGYRMEIRCLEKNGKQTTNKISYCAHHRAPNPDNVLIIQTPAGTISSKKLVQSNG

TVAASRLIRKDLPKDSVSEVEISENLSAARCRFYVKKELKRSREGAIAHRVRGSCQHRWD

EIDSLNPPREERDPESFSTFKERLHYLQKTEHTRVCFGRSGIHRWGLFARRGIQEGEMVL

EYRGEQVRRSVADLREEQYRVQGKDCYLFKISEEVVVDATDKGNVARLINHSCTPNCYAR

IMSVGHDESRIVLIAKKNVSAGEELTYDYLFDPDEADDRKVPCLCQTANCRKFMN*

>OsSET3_SDG729

MRNSATPGAVGELAELVLPWLPPQDLAAAASASRALRAAASSVSAGRAADAAHGLEPHPI

PFDNLVDGKPYAYFLYTPFSLTPSSASASPRRAQPWGRTWARPPGPTWPRSDLGGFPSSG

CACAQGACGGARGCPCADPEAEAVGLGSEAGMGSLRECGDGCACGPSCGNRRTQLGVTVR

LRVVRHREKGWGLHAAEVLRRGQFVCEYAGELLTTEEARRRQGLYDELASVEISSKEKSS

PSVMVMLDLGRMACRAFALGMHFPLQESGGDEDESFDKEDSWREWRTQQKNNLVPLQSFI

QSTLPFPLSLAFVSSVQQPQLMPMPPLPMPHVPFALALQVLSE*

>OsSET4_SDG709

MNRESNFMPTPDQDVLEVKPLRTLAPMFPAPLGIDVLNRLTAPPLVFVAPAGQFPGGFGS

LNIPAVRSFAAFGGQDASGGKTAGGGDQDASGGKTAAGGDQDAGRGETAAFGGQETVRGE

FVANGTPNVGASATGPIDATPISACKSTQPSVISLDDDDNDDDEPYGGNQTSASGRKIKR

PSHLKGYNVSDGLGTDSSNGTKKRPKTSNRKAATDNEISLMPPSSDPREVVEVLLMTFEA

LRRRHLQLDETQETSKRADLKAGAIMLASNLRANIGKRIGAVPGVEVGDIFYFRMELCII

GLHAPSMGGIDYMNKFGDEDDSVAICIVAAGVYENDDDDTDTLVYSGSGGISRNSEEKQD

QKLERGNLALERSLSRKNVIRVVRGYKDPACLTGKVYIYDGLYKIHESWKERTKTGINCF

KYKLQREPGQPDAVAIWKMCQRWVENPAARGKVLHPDLSSGAENLPVCLINDVNSEKGPG

HFNYITQVKYLKPLRSMKPFQGCRCTSVCLPGDTSCDCAQHNGGDLPYSSSGLLVCRKLM

VYECGESCRCSINCRNRVAQKGVRIHLEVFRTTNRGWGLRSWDPIRAGSFICEYVGEVVD

DTKVNLDGEDDYLFRTVCPGEKTLKWNYGPELIGEHSINISADTFEPLPIKISAMKMGNV

ARFMNHSCNPNTFWQPVQFDHGEDGYPHIMFFALKHIPPMTELTYDYGDIGCESRGVGSR

AKNCLCGSSNCRGFFS*

>OsSET44_Os01g65730

MEASASTSTARRLRAFRRWMRDHGVVCSNALRLDAAEDGGGGVYVRALAALREGDLVATI

PRGACLTPRTSGAAEAIEAAELGGPLALAVAVMYERARGAESPWDAYLRLIPEREPVPLV

WPADEAERLLAGTELDKIVKQDRQFICEDWKECIEPLILSGELEVDPDDFSLENYFSAKS

LLSSRSFRIDSYHGSGMVPLADLFNHKTGGEHVHFTSVLEASDSDSEDGEDPNNASADEQ

STIENSADIPSGDDDEDLEMIVVRDVNEGEEVFNTYGTMGNAALLHRYGFTEMDNSYDIV

NIDLALVTKWCSSKYSRRYARARVSLWHNLGYSGCTSQDADYFEISYDGEPQLELLILLY

IISLKSDAYDKLASVAHDLIGDDEVDSISSVLKVVRVTSSNQHPDISGLEKLPDVKKLLL

NESVCSALVSLVDMRESLYGSNTLEDDRQKLQACSSVNERNLYHSLVLRVSERTILHKLK

KHASSWSKTKKRKQL*

>OsSET5_SDG714

MEVMDSVAVMEVSPVPKPPLEAALALRRSVRCLNRTRRPTYVEQEEPKESAGRRRGGKRK

REEEKKEPVAQHAMKPVRMGDAASERKPSSEGKPMPAIAAEPVSCAGFARPAAEDDVLGN

GKSAKLRVKETLRAFTSHYLHLVQEEQKRAQAVLQEGQKRPSKRPDLKAITKMQESNAVL

YPEKIIGELPGVDVGDQFYSRAEMVVLGIHSHWLNGIDYMGMKYQGKEEYANLTFPLATC

IVMSGIYEDDLDKADEIIYTGQGGNDLLGNHRQIGSQQLQRGNLALKNSKDNGNPIRVIR

GHISKNSYTGKVYTYDGLYKVVDDWVQNGVQGHVVFKYKLKRLEGQPSLTTSEVRFTRAE

APTTISELPGLVCDDISGGQENLPIPATNLVDDPPVPPTGFVYSKSLKIPKGIKIPSYCN

GCDCEGDCANNKNCSCAQRNGSDLPYVSHKNIGRLVEPKAIVFECGANCSCNNNCVNRTS

QKGLQYRLEVFKTASKGWGVRTWDTILPGAPICEYTGVLRRTEEVDGLLQNNYIFDIDCL

QTMKGLDGREKRAGSDMHLPSLHAENDSDPPAPEYCIDAGSIGNFARFINHSCEPNLFVQ

CVLSSHNDVKLAKVTLFAADTILPLQELSYDYGYVLDSVVGPDGNIVKLPCFCGAPYCRK

RLY*

>OsSET6_SDG720

MGRRALPPSSSSSSSSSTTTTSPELRRKRTAAPPPPPSPRRYRSISDVMRRSLPVDAAPP

VARAYESTRCDVCGSGERDEELLLCDGCDRGRHTFCLRPIAARVPTGPWFCPPCAPRSKP

VKRFPMTQTKIVDFFRIQKGAEDAEAEKYGLFQDVKKRRKRSLVMHKKRRRILPYVPTED

KVQRLKQMASLATAMTSSKMKFSNELTYMPGMAGRSCNQATLEEGGMQILPKEDKETIEL

CRTMQKRGECPPLLVVFDSREGFTVQADADIKDMTFIAEYTGDVDFLENRANDDGDSIMT

LLLTEDPSKRLVICPDKRGNISRFINGINNHTLDGKKKKNIKCVRYDIDGESHVLLVACR

DIACGEKLYYDYNGYEHEYPTHHFV*

>OsSET45_Os01g74500

MRFRFAPRRCAAAAAASASKGGGGGGDCDCSVFLRWLRSKSGTHISSVLSLGTSSAFGRS

LFASEPIQEGDCIMQVPYHVQLTLDKLPQKFNTLLDHAVGDTSKLAALLIMEQHLGNVLW

DLNELHAVQNSSIYDEAIEHKEQAKKEFLALKPALDHFPHLFGEVKLGDFMHASALDFLN

HDGVFGSVLIYDEQKDVCEIIADRNYAVGEQVMIRYGKYSNATLALNFGFTLARNIYDQA

LIRIDMPVQDPLYKKKLDIWQKHRLPIFEDMCNLSSATSFVINMLNLEIE*

>OsSET7_SDG730

MGPATPLRRRTRARPAATRAEGGSGGDGDDDDVRCEACGSGESAAELLLCDGCDRGLHIF

CLRPILPRVPAGDWFCPSCASPSPHSKKSHAAKKPKQFPLVQTKIVDFFKIQRGPAAALA

AAAESSEGKKRKRKVGGIRLVSKKKRKLLPFNPSDDPARRLRQMASLATALTATGAVFSN

ELTYVPGMAPRAANRAALESGGMQVLPKEDVETLNLCKRMMARGEWPPLLVVYDPVEGFT

VEADRFIKDLTIITEYVGDVDYLTRREHDDGDSMMTLLSAATPSRSLVICPDKRSNIARF

INGINNHTPDGRKKQNLKCVRFDVGGECRVLLVANRDISKGERLYYDYNGSEHEYPTHHF

V*

>OsSET8_SDG725

MRKGRNVGKQECRKKDGKKGKNINKNRSSTKISSSEASKLVSFSNDSPSLDPSELLLHTR

PPKFGSCSKVVTSAIHDVGMHGYDNMRPFGIDNDDEGSAFDNVKSLRRKKKDSHGGKKGK

VRDPHGKGRSKKKNIADNTYGLPAQLTDLSEPRMNKQSDLIPAAELVFKNSSAVSVELPA

VVACKTDGASVPPAPAWVCCDDCEKWRCIPTELADKISKENLRWTCKENEDKTFANCSIP

QEKTDDEINAELGLSDASADEANGDGSNSKASGEPNFALLRSNLFLHRNRRTQSIDESMV

CNCKPPHDDRMGCRDGCLNRILNIECTKRTCPCGEHCSNQQFQRRTYAKLGKFHTGKKGY

GLQLKEDVSEGRFLIEYVGEVLDITAYESRQRYYASKGQKHFYFMALNGGEVIDACTKGN

LGRFINHSCSPNCRTEKWMVNGEVCIGIFAMRNIKKGEELTFDYNYVRVSGAAPQKCFCG

TAKCRGYIGGDISGADMITQDDAEAGTFEPMAVQEDAEEVLGANGLSSHGTHLDIVDHEA

STKTEDSNDCPSVNPPELESEQQTSGTLFDTSEPENSLEALSPQDDEDVVRTPVHVSRTV

ESTSRQFPEYGTRSSEILQRAPCTLDGPKVPSTTNGIPPSSDLGSHWVPGFHANKKTNVK

HHLILNPSSAPIDSEHILGVEGRLNSLLDVNGGISKRKDATNGYLKLLLVTAAEGDNAGG

TSKSVRDLSLILDALLKTRSNSVLLDIINKNGLQMLHNILKQNKSDFHRIPIIRKLVKVC

ISFLCYVLLH*

>OsSET9_SDG736

MLMKTSRLIQPSGISLQLSANHHTLRLSDAKKRPDSRAEAGCTNCSADSTCKDDCECRGL

YMSCSKNCHCSDMCTNKPFRKDKKIKAVKTKRCGWGAISLEPLEKGDFIIEYVGEVINDA

TCEQRLWDMKRRGDKNFYMCEISKDFTIDATFKGNTSRFLNHSCDPNCKLEKWQVDGETR

VGVFASRSIQVGEHLTYDYRYSSCIEKLLSCNQLWLTMI*

>OsSET10_SDG712

MAKPNGKEKTGDTGLSMAPPKISKDRFDAAIRAMADIGILKETAAPVLNNLLNLFDYNWV

HIEADNYLALADAIFCDSDPKEGQKRQANETNLDADQSNKKLKTKKRSQNPTSKMHGNDN

REFVEAPPQQGRGTLSARTVNGKKVTRAHLELPSSQLLIKEPHTCPSIAKNTTIVENNSA

VLCHGQDLQTFEVPVATTCPQVVAPSTRKDARRTSGARHDQKHEGVSGAHERNRAVACSN

QEIVSSKDSPSNIEVVLSNYGAGKLSFTYNSSLANRSDFHLPDIKLICKKMEARCLRKYK

SLEPNFSFKNLIKDTCQCIVESSGPRHEGIIQTVPALDILSKPSVPQILQSNQANSAFMP

PNNVMSLGGTSSSCTVAGVSQNSSNMPVVPHQLHIGANRPPHDVNDITKGEERLRIPIIN

EYGNGILPPPFHYIPHNITLQEAYVNISLARIGDDNCCSDCFRDCLAQSLPCACAAETGG

EFAYTTDGLLKGAFLDSCISMIREPLKHPHFYCKICPNERMKIEVNSDSSNTEMNPGPCK

GHLTRKFIKECWRKCGCTRNCGNRVVQRGITRHLQVFLTPEKKGWGLRSTEKLPRGAFVC

EYVGEILTNIELYDRTIQKTGKAKHTYPLLLDADWGTEGVLKDEEALCLDATFYGNVARF

INHRCFDANIIGIPVEIETPDHHYYHLAFFTTRIIEPFEELTWDYGIDFDDVDHPVKAFK

CHCGSEFCRDKTRRSKSRARV*

>OsSET11_SDG706

MMFDLLPDLGHHHQVAHTNSGTVSDIPSGREKYQFNRGRHYYSAFKKSLRPSGSLKKRTS

SGVEKHFKAQSLDLSMDTSHIVESETTTLGRLLDFQCSDVALTLFSKIQKTRPHPSNLDI

LSIARSVCCKTSLRAALKAKYGILPDNIFVKAAKLCSDVGIQIDWHQEEFFCPKGCKSRS

SSNSLLPLQPTQVDFVMSPPIGDEIWGMDEYHYVLDSEHFGWNLKNEMVIVCEDVSFGRE

KVPVVCAIDVDAKEFPYMKPGEILQSENSLPWQGFHYVTKRLMDSSLVDSENTMVGCACS

HAHCSPEECDHVSLFDSIYENLVDLHGVPMRGRFAYDENSKVILQEGYPIYECNSSCTCD

ASCQNKVLQRGLLVKLEVFRTENKGWAVRAAEPIPQGTFVCEYIGEVLKMKDDGAIRHVE

REAKSGSSYLFEITSQIDRERVQTTGTTAYVIDATRYGNVSRFINHSCSPNLSTRLVSVE

SKDCQLAHIGLFANQDILMGEELAYDYGQKLLPGDGCPCHCGAKNCRGRVY*

>OsSET12_Os02g49326

MATAAALALHTQFRPPRSPRRLRQHLALPSGVLIRSPVRASAASASASAPAQREAAAAGV

PWGCEIESLESAVSLERWLTDSGLPEQRLGIQRVDVGERGLVALKNIRKGEKLLFVPPSL

VITADSEWGCPEVGNVLKRNSVPDWPLIATYLISEASLESSSRWSSYIAALPRQPYSLLY

WTRPELDAYLVASPIRERAIQRITDVVGTYNDLRDRIFSKHSDLFPEEVYNLETFRWSFG

ILFSRLVRLPSMDGRVALVPWADMLNHSPEVETFLDYDKSSGGIVFTTDRSYQPGEQVFI

SYGKKSSGELLLSYGFVPKEGTNPNDSVELLVSLNKSDKCYKEKLQALKRNGLSEFESFP

LRVTGWPVELMAYAFLVVSPPEMSQRFEEMAVAASNKSPSKPGLNYPELEEQALQFILDC

CESNIAKYTKFLEGSSGSLQLSTNSKQANRTLLLKQLARDLCISERRILYRTQYILRRRL

RDMRGGELKALSLFNGLRKLFK*

>OsSET13_Os02g50100

MAAAAAAGATPATARKALLTTTATLLSSSLARSRRSLSCSAAAASAAPRIAPQPPDLLRW

VQREGGFVHPALRVVDHPEHGLGVSAAAAEGDIPPGDVLIALPGRLPLRLRRPAGAADAV

LVQLADQVPEELWAMRLGLRLLQERAKSDSFWWPYIANLPETFTVPIFFPGEDIKNLQYA

PLLHQVNKRCRFLLEFEKEVKHKLGTVPLEDHPFCGQDVNSSSLGWAMSAASTRAFRLHG

EIPMLLPLIDMCNHSFNPNARIVQEGNVDSPDMSVKVVAETKIDQNAAVTLNYGCYPNDF

FLLDYGFVITSNSYDQVELSYDGTLLDAASMAAGVSSPNFSAPAKWQQDILSQLNLYGEG

AILKVSIGGPEIVDGRLLAALRVIIAADPDAVSGHDLKTLMSLKEKAPLGPAVEASALRT

VLALCTFALQHFHTKIMEDEAILKGEPPLTTELAVQFRLQKKLLLLDVIQNLSRRIKMLA

LDKSTV*

>OsSET14_SDG739

MATPGLDDDSLQQLRSRATQLLLKENWTEYIAVCSLIIEAFDAAAACKDRRVLCSTLAHR

ADARARLGDAPGALADCDAALAADPAHPGALLSKGAVLRGLGRYSRAAECFRAALAVSGT

DEVREMVEQCKRLDAQARSGAVDLSEWVLAGFSGKCPDLAEHVGAVEVRRSAHGGRGVFA

VKNIEAGANLVISKAVAIGRGVIPDAADSGEKMVVWKDLVDKVLDAAEKCPRTASLIYTL

STGEEPEDELPIPDMAHFKQETEELDDGTAMAPKASLDVDKILKVLDVNCLTEDAAPSAN

LLGSNGVVNCGVGLWILPAFINHSCHPNARRTHVGDHAIVHASRDIKAGEEITFAYFDVL

TPASKRREAARAWGLECQCDRCRFEASDAIVGQELTKLENELVNGRGGDMGALVVRLEER

MRKSMVKERRKAFLRASFWSAYSALFDSDKLVRKWGRRVPGEAAVAESVAGAIGGNESVL

RAMLRGADNGNGCGNRLEVEDKVVRIGRATYGRVVKRQAMRALFRLTLDADSNKSL*

>OsSET15_SDG718

MASSSSKASDSSSQRPKRPDQGPSGKDAAGLVALHGKLAQLKRQVQSTRLAAIKERVEAN

RKALQVHTCALFDVAAAAEVASRGAEGGNALSRGAAEGHRRFVGWDSASGPGERELVHVQ

EENLVAGTLVLSSSGGSGASHRTVVQLVKLPVVDKIPPYTTWIFLDKNQRMADDQSVGRR

RIYYDPIVNEALICSESDDDVPEPEEEKHVFTEGEDQLIWKATQDHGLSREVLNVLCQFV

DATPSEIEERSEVLFEKYEKQSQSSYKTDLQLFLDKTMDVALDSFDNLFCRRCLVFDCRL

HGCSQNLVFPSEKQPYGHELDENKRPCGDQCYLRRREVYQDTCNDDRNACTTYNMDSRSS

SLKVSATILSESEDSNRDEDNIKSTSIVETSRSKITNSEYADKSVTPPPGDASETENVSP

DMPLRTLGRRKISKHASKSNDHSPDKRQKIYSSPFPFAMSVLNKQSVPEIGETCPDSIES

AVDQLPSLDDPNKKISTKDMCAGSTTNTTENTLRDNNNNLFISNKEHSISHWSALERDLY

LKGIEIFGKNSCLIARNLLSGLKTCMEVASYMYNNGAAMAKRPLSGKSILGDFAEAEQGY

MEQDLVARTRICRRKGRARKLKYTWKSAGHPTVRKRIGDGKQWYTQYNPCGCQQMCGKDC

ACVENGTCCEKYCGCSKSCKNRFRGCHCAKSQCRSRQCPCFAASRECDPDVCRNCWVSCG

DGSLGEPLARGDGYQCGNMKLLLKQQQRILLGKSDVAGWGAFIKNPVNRNDYLGEYTGEL

ISHREADKRGKIYDRANSSFLFDLNEQYVLDAYRKGDKLKFANHSSNPNCYAKVMLVAGD

HRVGIYAKDRIEASEELFYDYRYGPDQAPAWARRPEGSKKDEASVSHHRAHKVAR*

>OsSET16_SDG713

MESNQHKASDPQDSMVHLDLDEDKIMVTSALPCPSMSVGKSVMRKRGRPSRHARGTSLSS

VTPEGCKKMEGRSYNLRSDSTILLRNSCLLIADGSTKQKRSWGLDKDDLHIPFFQISDNP

REAVDDILMTFGGLHRRIMQLIDVKMASKQLVFQALNLMRKVGYHVNKDKRVGEVPGVKI

GDIFYSRIEMLLVGLHSNINRGIEFMSGAFINKEDKIATCIVSSGMYENGDDDPYTLVYN

GQGKVHHKLERGNYSLNQSFIRRNHIRLIRSEPNPLVRLGSKEKIYIYDGLYKIEEKYRQ

TTKSRSNLKFNKLVRELGQPNGIVVWKNTQKWRENPSCRDHVIMPDMSNGAEIARVCVVN

NIDSEDAPNNFTYSTKLDNGNHMVSANKMCVCKCTSSCLGEDNCSCLKTNGSYLPYNSSG

ILVCRKTMIYECNDSCACTINCSNRVVQRGSYLHFEVFKTMDRGWGLRSWDPIPAGAFVC

EYVGVVIDKDSLVEEDEYIFEVTRPEHNLKWNYLPELIGEPSFYDMNDTFKKLC*

>OsSET17_SDG716

MNLFLFFFSNLLERRGVGAGGMASWEEQLRDELAGRDLAVASVPGKGRGLFAARSFFPGE

VVISQEPYASTPNKISVGSNCDNCFASRNLRKCSVCRVAWYCGSACQREEWKLHQLECRA

IAALTEDRKKMLTPTIRLMVRLVLRRKLQDDKAIPSSGTDNYNLVDALESHISEVDKNQL

VLYAQMANLVQLILPSFELDLKEITHTFSKFACNAHTICDPELRPLGTGLYPVLSIINHS

CVPNAVLIFEGRTAYVRALQPISKNEEVSISYIETAATTMKRQDDLKHYYFTCTCPRCVK

DSEEDALLEGYRCNDQKCDGFLLPNAGNKGYTCQKCSTSRDGEELQKMASDVLLLSDKVS

SLVSSGIDNSEVGSMYKTIEELERKLYHPLSITLLHTRETLLKIYMELQDWQTALMYCRL

TIPVYERIYPPFHPMIGLQFYTCGKLEWLLEYTEDALMSLTRAADILRITHGTKSEFMKE

LLGKLEEVRAEASFRLSAGDEQ*

>OsSET18_SDG738

MAMGMRARESVNMSEDLTQAIAPYATALHDASLQSHCSSCFHRIPAQSPHDMSCTMCGSV

RYCCSDCLISDCEVHSSSGECCFFVKHLREASPSTLTEETSDIRAALRLLYSLETRGLVS

SDSVSSSNRIGGLSASGIREVLEEGGEIAEGVLEGSLLMLSARKSRMKNYVGLSNGLTIE

KVALWAVMTNSVEVQISEEQSLGIAVYGPSFSWFNHSCCPSASYRFVLVPQNEGCTSNKP

ESCVVPVSKGAAPDAWHAWQNEEAGFAHAQCKYGPRVVVRCTKPINKGDEVFITYIDLLQ

TRIVKYCYRIGLVKVQGLRCDARNLKSPHNAVTDPAIEDLDNNLQQAISEYSFLDDSKAC

CDVIESMLSENLMNDLQQEELSPRKYILHPLHHISVSSFMILASAYRCSAFKSSTDNLHG

ENCDFIFRMTKAAAAYSIVLAGATHHLFLSECSFVTLLSHFLLSTGQSILDFAECIKGET

RKNMPEAIFSFASCSTNSAKHDSVRYNQFRSTCEKFGKPLLSLSLQCWPFLAQGLPCLEK

IKNPIDFSWLGPAIFQAFQLSEEDSFNLSGKHAPATLIEQQKECILSLAVCCITYSKYLA

RSKSFLFVTLGRLEESSSDWAVKIFDLNNGAKVYSRMQEYYLHFAKCLLIPTSINFFRVM

SVPNDQLTNIGPTYKYGKNHFEHVDRRSGFPMSMIAAATEGLVLMTRKVATTRPTLEIMA

AAVTTTVVTLASSTSPATTTMRVATRTPTLMNMAALVATTSPTPTT*

>OsSET19_SDG708

MEPPPPPPYIHIETNDFLHRRHKRQKEEDIAVCECQYNLLDPDSACGDRCLNVLTSTECT

PGYCLCGVYCKNQRFQKSQYAATRLVKTEGRGWGLLADENIMAGQFVMEYCGEVISWKEA

KRRSQAYENQGLTDAYIIYLNADESIDATKKGSLARFINHSCQPNCETRKWNVLGEVRVG

IFAKQDIPIGTELSYDYNFEWFGGAMVRCLCGAGSCSGFLGAKSRGFQEATYLWEDDDDR

FSVENVPLYDSADDEPTSIPKDILIKDEPNTQDGNNNTIQNTGIPIIASSSEFTPMNVEP

SIASSNEFTPMNVEPLNVSSNELTPMTIEPLNAIPMGVDFTQNGSIEYGAQCAEDALQNS

TRGVANLQNQSAPRDNNHTELVAVKRRPTLRGGKAKRGMRKQLNVVGICDRLASEVAREE

ILYCEEMKNEAAAEIDSLYDEIRPAIEEHERDSQDSVATSLAEKWIEASCCKYKADFDLY

ASIIKNLASTPLRSKEDAAPTEQNGLMYLENGP*

>OsSET20_SDG703

MGIPEVVVPPRAAGPRRYKGLVPWRFQPGFVRPPPVKPPAAAAAVAGGGVAGTPGGKGRG

LGASGEGVGSSGGRGDPQSRRCTRSASAKGSGDARSVEEGGPRVAGDDGGSGKSGVAAEG

SGFEGLRNGRGGGVGTAAAEDCGLEKSNPDGIVGDADVHLESGSDARDGECVSEGLKKPC

VNNSNGSSAADCAPKVKKGNDSGNGGADECNAAAKSSNLACPGNNGDETNRKGRKVVLPW

RFQVGFKRSFSKAFCSDSESSGPSGTQFYRAQDSSTPCTPATRSSVRCYASAHSGVRVSA

MRDFSVKGEKETSTPYKKSKTGMDGPSQGMPKNGVVLARENIMGSLQNFRLIYRDLLDEE

EEKSTEAVIRPDLQAYRIFRERFITDCDEKKYIGNVPGIKVGDIFHLRVELCVVGLHRPH

RVGVDHIKQEDGTCIAVSIVSYAQSSDIKNNLDVLVYSGAMTAIANQKIEGTNLALKKSM

DTNTPVRVIHGFVTHLNGNCQRKKIPTYIYGGLYIVEKYWREKEGNDRYVYMFRLRRMAG

QKHIDIQDILNSGQAESYGGIIIKDISRGLEKIPVSVVNSISDEYPMPYRYIAHLQYPRN

YQPAPPAGCGCVGGCSDSKRCACAVKNGGEIPFNDKGRILEAKPLVYECGPSCKCPPTCH

NRVGQHGLRFRLQVFKTKLMGWGVRTLDFIPSGSFVCEYIGEVLEDEEAQKRSTDEYLFA

IGHNYYDEALWEGLSRSIPSLQKGPDKDEEAGFAVDASKMGNFAKFINHSCTPNLYAQNV

LYDHDDKSVPHIMFFACEDIPPRQELSYHYNYTIDQVHDANGNIKKKKCLCGSIECDGWL

Y*

>OsSET21_SDG722

MFISTPLPQVRDKVSRTKPPKPHGGGGERRRKKQPQEAAARAGGGMGGSSASPCDLDREF

APQIAQLLATPPLQPAQEYYNGLIQSRKHDGIRVNFSSKHGKGVCANKEFAEGDLILKDQ

ILVGAQHSLNKIDCAVCSYCFRFIGSIEFQIGRRLYWQSVGSSSDCTNRRHCHESDLGSS

ASSSGATKENSSTLPEEVLGSLITGDMSLPFTDHFSLPQVVPCRGCEEERYCSQSCADSD

WETYHSLLCTGSKTEPSQRSALQKFIEHANGSNDIFLVAAKAITFTLLRYKKLKTQPEFQ

NNTDESNFSLLMEAWKPLSMGYKKRWWDSVALPEDVDSCDEDTFRQQIRDLALTSLQLLK

DAIFDSECAPLFSLDVYGHLIGMFELNNLGLVVPSPVEDYFIHIDDLPDDEKEEAEKVTR

PFLDALGEDYAAPCEGTAFFPLQSCMNHSCCPNAKAYKRDEDTDGNAVIIALEPIKKDDE

ITISYIDEDVSYEERQAELADYGFICTCPRCQEEKPN*

>OsSET22_SDG728

MDRASNFIPGPYQELVDAKPIRSLAPMFPAPLGINVNQSSTPPLVCVTPVGQFPVGFGSG

ILPTFGSTTAFTTTANGVSYTSYTNNGAIDATPISAYKTRPGIVSLDGDEPYSGSASGRK

SKRSSGSAADGSNGVKFKRPKPVYKNFVAGKELAFLPPSSSDPREVVEAVHMTFEALRRR

HLQLDEIQETSKRADLKAGAIMMASNIRANVGKRVGLVPGVEIGDIFYFRMELCIIGLHA

PSMGGIDYMSAKFGSDEDSVAICIVAAGGYENVDDDTDTLVYSGSGGNSRNSEERHDQKL

ERGNLALERSLHRKNEIRVVRGFRDPFCLTGKIYIYDGLYKIQESWKERTKSGINCFKYK

LLREPGQPDGAALWKMTQGWIDNPASRGRVILPDLSSAAEALPVCLVNEVDHEKGPGHFT

YASQVKYLRPLSSMKPLQGCGCQSVCLPGDPNCACGQHNGGDLPYSSSGLLACRKPIIYE

CGDACHCTTNCRNRVTQKGVRFHFEVFRTANRGWGLRCWDPIRAGAFICEYTGEVIDELK

VNLDDSEDDYIFQTVCPGEKTLKFNFGPELIGEESTYVSADEFEPLPIKISAKKMGNVSR

FMNHSCSPNVFWQPVQHDHGDDSHPHIMFFALKHIPPMTELTFDYGVAGSESSGSRRTKN

CFCGSSNCRGVF*

>OsSET23_Os05g50980

MATPAASPLLLPLPLPLPASTFPPRRAVPCARRLVLRPPRAGRPRLRDPPPAAPPPAAEE

VGEEEEDDDAPPLRLLEPPQEDDPFPPEMEPADPDFYRIGYARMMRAYGVEFLEGPDGMA

VYASRDVDPLRRARVIMEIPLELMLTITQKRPWMFFPDIIPLGHPIFDIIESTDPETDWD

LRLACLLLYAFDVEDNFWQLYGDFLPSVDECTSLLLAPKHKTIPLKLKRLAPDHERFLWA

LSIVQSRSVNLKLRMGAFLQDANVLVPYADMLNHSPDANCFLHWRFKDRMVEVMIKAGHA

VKKGDEMTIDYMSGVNSSFMERYGFSSPTNPWELINFSSDAKIHLDSFLSVFNIAGLHDE

LYHNAALTSGENNFVDGGVVAAARTLPTWSEGDVPAIPSLERKSAQALQEECHTMLESFS

TTIQQDQEILDSDGHIRRTREIAIKYRLHRKLLLQKIIDALDIYQDKILF*

>OsSET46_Os06g03676 Evalue0.002

MAAAAAAAAVPGDAKLDSFLQWLQANGADLRGCTIRRCGREGYGVFSTAAEAGATDEVVM

VVPLDLAITPMRVLQDPLVGPRCRALFEEGGVDDRLLVMLFLMVERLRPSSLWKPYLDML

PSTFGSSIWFTEDELAELEGTTLHRATVMQRKSLQTLFDNKVKGLVGELLNVDESGSSIE

VRFEDFLWANSIFWTRALNIPLPRFYVFPESLDEKRANIGDDCGDSSLSAPQGTGTAITA

KNISGNDNPKSSNTESIWVEGLVPGIDFCNHNVKALATWEVDSMGHVTGCPSSMYLVLAD

KSFVKAETEICINYGNKGNEELLYLYGFVIDNNPDDYLMIHYPVEALRQVQSADIKMKLL

EIQNAELRCLLPRSLLENGFFGSCSGENKENKNNTSPFSSYSWSGQRKVPSYIEKIVFSQ

EFISTLRTIALQEHELEHTASLLGEIGSNEDRDDELRSAIWEVSGDNGALSLLVDLLRVK

MTELEEGTGTEASDSQLLEKFDLSDSEDATSDESNETKSKVNIRTCIVYRRGQKQLTKLF

LREAEHLLELSSKEEN*

>OsSET24_SDG711

MAGDSRNEPMFCEEGSSESGYVLCVIDSLKKKITSDRFVYIQKRVEENSIKLSPITLHSH

NLSKNRQTSTSNSTDLVSNLLTKRKEDALCAVNSRESSPDESEGANCQDECSSTVIVGGN

LSARNSVRPIRLPEVATLPPYTTWIFLDRNQRMQEDQSVLGRRRIYYDTNCGEALICSDS

EDEAVEDEEEKKEFKDSEDCIIRMTIQECGMSDAVLETLARDIERAPDDIKARYEILQGE

KPEGSSKKVSELNVKMEDVYGDKDLDAALDSFDNLFCRRCLVFDCKLHGCSQDLVFPTEK

QAPLCSSDEGTPCGIHCYKLVSKPDAIMEIDSHLLVDVEEPTSDNLKDQIGSNKKKLGSS

GQKTKSQQSESSSTARVSSESSESEVQLLSNKSPQHSPGLSKNKLGAKGGIKKSTNRRIA

ERILMSVKKGQQEMSPDSNSIVNGCHWPRDMKLRSDTRSGIKDSVVSSQCNSPSTRSFRK

KGTLQMENNSSFVDAQSDSMEDTNNEHSATDGCDSSRKEECVDESICRQEAHGRSWKVIE

QGLLLKGLEIFGKNSCLIARNLLGGMKTCTDVFQYMNYIENSSASGALSGVDSLVKGYMK

GNELRTRSRFVRRRGRVRRLKYTWKTAGYHFIRKRITERKDQPCRQYTPCGCQSACGKQC

PCLTNGTCCEKYCGCPKMCKNRFRGCHCAKSQCRSRQCPCFAADRECDPDVCRNCWVGCG

DGTLGVPNQRGDNYECRNMKLLLKQQQRVLLGRSDVSGWGAFLKNSVGKHEYLGEYTGEL

ISHKEADKRGKIYDRENSSFLFNLNNEYVLDAYRMGDKLKFANHSPDPNCYAKVIMVAGD

HRVGIFAKERISAGEELFYDYRYEPDRAPAWARKPEGPGAKDDAQPSTGRAKKLAH*

>OsSET25_SDG726

MEMDTSPSSSAPSSPAASSDSIDLNFLPFLKREPKSEPASPERGPLPLPAAAPPPPPPPP

PPPPPPQVQAATVATPVPATPDLSAAAVMTPLQSLPPNPEEETLLAEYYRLATLYLSSAG

AAGVIVPAAAPEASAGAVAQPGSGSGAKKRRPRSSELVRVSSLSVQDQIYFRDLVRRARI

TFESLRGILLRDDERAEVLGLTGVPGFGAVDRRRVRADLRAAALMGDRDLWLNRDRRIVG

PIPGISVGDAFFFRMELCVLGLHGQVQAGIDFVTAGQSSSGEPIATSIIVSGGYEDDDDR

GDVLVYTGHGGRDPNLHKHCVDQKLEGGNLALERSMAYGIEIRVIRAVKSKRSPVGKVYF

YDGLYKVVDYWLDRGKSGFGVYKYKMLRIEGQESMGSVNFRLAEQLKVNALTFRPTGYLG

FDISMGREIMPVALYNDVDDDRDPLLFEYLARPIFPSSAVQGKFAEGGGGCECTENCSIG

CYCAQRNGGEFAYDKLGALLRGKPLVYECGPYCRCPPSCPNRVSQKGLRNRLEVFRSRET

GWGVRSLDLIKAGTFICEFSGIVLTHQQSEIMAANGDCLVRPSRFPPRWLDWGDVSDVYP

EYVAPNNPAVPDLKFSIDVSRARNVACYFSHSCSPNVFVQFVLFDHYNAAYPHLMIFAME

NIPPLRELSIDYGMIDEWVGKLTM*

>OsSET26_SDG731

MEALLRWAAELGVSDSPSAPSPSSCLGRSVLIADFPDAGGRGLAAARDLRRGELVLRAPR

AALLTSGRVMDDDPRIASSVASHLPRLSSVQTLIICLLSEVGKGKSSNWYLYLSQLPSYY

TILATFNDFETEALQVDEAIWVAQKALRGIRSDWEEATPLMKGLGFKPKLLMFKSWIWAF

ATVSSRTLHIAWDDAGCLCPIGDLFNYAAPNDDNSSTDEDRDDMMHQETNKMLDQTDFDS

SEKLTDGGYEDVNEYRLYARKRYRKGEQVLLAYGTYTNLELLEHYGFLLGENPNEKIYIP

LDLDLCMIGSWPRDSLYILPNGHPSFALLCALRLWTTPRNRRKALSHQIYSGSLLSVENE

LEILKWLVKKCKETLQQLPTTIEFDDNLLVLLCKLQNSTSCITEMNRSIFEQEFAPFFRF

HGFKLDCSIHSKLPVRLLRSLERWGLAVQWRCNYKRTLTKCIVHCKSLVHELSLQQNQQ*

>OsSET27_SDG701

MKSSRFDFFVPKVAKIEVRLKNGYYARHGFSYIKNDIRSMCRDALRYKGRSDLGDMKQIV

VAFIQLAKKLENPRLISDRDGTAVQKDSSDMSQYSSDLKLKKKQSKTMSERRGANWTTAG

ADPSSRAFDREIKRSLSKLKKRDIDSGSETSDDDDGYSEGDETESETTVSDTESDLDVNS

GAWDLKGNGMKLFESSESLTDDRGWGARMTKASLVPPVTRKYEVIEKYLIVADEEEVLRK

MRVALPDDYSEKLLSQKNGTENLELPEVKDYQPRKVPGDEVLEQEVYGIDPYTHNLLLEM

MPTELDWPSSDKHTFVEELLLNTLNKQVRQFTGSGNTPMVYPLKPVIEEIQKSAEESGDR

RTSKMCLGMLKAMRNHPEYNYVAYRKGLGVVCNKTGGFGVDDFVIEFFGEVYPSWRWYEK

QDGIKHIQNNSDDQAPEFYNIMLERPKGDRDGYDLVFVDAMHKANYASRICHSCNPNCEA

KVTAVDGHYQIGIYTVRPIAEGEEITFDYNSVTESKEEHEASVCLCGSQICRGSYLNFSG

EGAFEKVLMEFHGVLDRHSLLLQACEANSVSQQDLIDLGRAGLGTCLLAGLPGWLVAYTA

HLVRFIFFERQKLPHEIFKHNVDEKRQFFTDINMDSEKNDAEVQAEGVLNSRLQNLTHTL

DKVRYVMRCIFGDPKNAPPPLVRLTGRSLVSAIWKGEGSLVDELLESMEPHVEEDVLTDL

KAKIRAHDPSGSEDIEGEIRSSLLWLRDELRTLSCTYKCRHDAAADLIHMYAYTKCFFRV

RDYKTVKSPPVLISPLDLGPKYADKLGPGFQEYCKTYPENYCLGQLIYWYSQNAEPESRL

TRARKGCMSLPDVSSFYVKSVKPTQERVYGSRTVRFMLARMENQAQRPWPKDRIWVFKSD

PRFFGTPMMDAVLNNSPLDKEMVHWLKTRSNVFLG*

>OsSET28_SDG740

MAGDALRAADLPGRGRGLLAARSIREGEVILTEQPLLLYPASLASLPSFCSACFRSLSAA

ASPCPSCRAAGFCSPSCAAASHPRLLCTALSGGGGNGNLASAAEPHQEPLLFLLSAYSLP

EPSLRVLLSLSSAATPPPSDQDPGSLHAMVAALVPPQMLPPGFSPDLTAALLSKDRTNSF

SIMEPYRPEVPQPLRKARAYAVYPRASLLNHDCLPNACHFDYADRPGPGNTDIVVRALHD

ITEGREVCLSYFAANWQYKDRQQRLLEDYGFRCECERCQVESKWKQDDDSDGGDGDDTME

EEEEDGNGGEGGDDGMEQEEGDGGSDSDDDFPHSYFFVRYLCNHGECYGMLAPLPPLPNG

EPSHVFECNVCGNLKNEDEIDAPDGGDSSMAD*

>OsSET29_Os08g14660

MAAAAAEVAAAGGGGEMVVVRLPPLSQDDPLFQDKKRILDSRNLSCLFQVPNSCSAADAF

KVLDRIIQAARIAHMDELELYFTGDDDFGPLSTRNELESLNLLLKILNTLLLTANVGAMG

VLQVLRDEILIRLRSLELEDNDQMVVQIRNQNMEDSLLKWGEQHGVKTKLQIAFFEGAGR

GMVASENIDVGDIALEIPESSIISEELLCQSGMFLALKDLDSITTETMLLLWSIRERYNP

SSKFKIYFEALPANFNTGLSFGIDALAALEGTLLFDELMQARQHLRQQYDELFPMLCIKF

PDIFKQDVYTWDNFLWACELWYSNSMMVVLSSGKLTTCLIPIAGLLNHSVSPHILNYGRV

DKVTKSLKFPLSRPCKAGEQCFLSYGKHPGSHLITFYGFLPRDNPYDVIPLDLDTSVDEE

DSSSPSVTTSQTSHMVRGTWLSRLRGPPTYGLPHRLVSHLHAILGCNQNESAPEADNKEN

DRMVLETLLSIFTPMLEGLGEPDDFDRENACWDVNLALDYKDLQRRIVLSIVTSCTSGLA

MLDS*

>OsSET30_SDG710

MSGLDLREMLSSAARRHTWRRSGWGAEWGGGDDARSAALPARCPPPPPPRRPAAAGGRGD

EGSGGVRGRGEEGTSRGGSGARREDAAGTGRNAVAVVAGGSATSGAERVAARAEDGRRSM

DGPTLELSEMMLHAAQPWRSRCTQRDVRPGAVPPRPVAADGRGEGTSTVRGRVLEGTTRG

GGRGGGMEREREVVAPARNAVAVAGDLATHGGERVAGPLVAKEKRNGGGELGTKRGLEKR

APLPPPKRRVVSAKRQFPPDFGRDSAVPLGRGRGRGGGVRPSDGAPARAVLGEKVASAGN

GDSMANVHHHAVMDTVLMKSSHASDENLVAFKVGSPENGAEGAARGKGAHNGELLGKREV

LAQAVNLLPMRRTVSATHRFTAGCGRDAAAPLARREEGKVGSGLEVMPVDVGGGVSKEVM

ATDGSKHSVNQCTANIVGAVGVLDGTVQYQELEEGEVADEAYCDVESQKVVGCDSFDDSA

GERHEGVVPVTFAVTEVLTSHAYDEMMQIKALQEGGSDAAQETEHDLPMGGKCETILPDA

SPKCSFGGPSNEIVHGKRVLGSHGMKGEVPSLAIEDHGGIAQIDQELEDVEMTTGEYRVQ

DAQIATHVIPHESTTGRHEGGLCASAAAEDVKVMNKYKGTLPKGAAKSSMNIATGVFGDG

IMRSKILSTARKVVKPPVRASHKPPLNTLHRPFSTNSASFGHKKLKVKRPDQSKDIPMKI

ASTSGLAGKDNLIDEKALSLEDDDILKALVVHDGKLEVYLNVPSCVQLHRQHGSGNADDR

SKIRMLCRRFQFICRALLHAVEQGSLKIRRVDLAADKIIRKLPGFTKPGPTVGNVNGVEV

GDEFMYRVELALVGLHRPYQGGIDTTDYNGVLVAISIVCSGGYPDELSSSGELIYTGSGG

KPAGKKKDEDQKLERGNLALKNCIETKTPVRVIHGFKGQNREDNSHSRAKQILTFTYDGL

YLVVDCWTEGLKGSRIFKYKLQRIPGQPELPLHIAKGLRRSLSRPGLCIADISQGKEMDP

ICVINDVSNVHPTSFQYISRIKYPSWLTKRHPQHHGCDCSDGCIDSTKCFCAVKNGGKIP

FNSNGAIVHDKPLIFECGPSCRCHSSCHNRVSQKGMKIHLEVFRTANKGWGVRSLRSISS

GSFICEYVGILLTDKEADKRTNDEYLFDISHNCDDEDCSKGRPSTISSLNSSGGCSQTME

DVCFTIDASEYGNIGRFINHSCSPNLYAQNVLWDHDDQRVPHIMFFAAENIPPLQELTYD

YNYKIGEVRDLNGRVKVKDCHCGSPQCCGRLY*

>OsSET31_SDG707

MAGPPGPAPGTSSSSSSLPSRRRRRPPPRLGRRRGGDDQQPPHPPKASSEALPCAASPPA

RCRGGDHQPPHPPEAALEALRGAASPPARRRGGDLHPSHPPEAASEALRGAASPPERRRG

GDRQPSHPTEAASEAPSGSASPPARRRGGDQQPPPAVAVAVTSEGGVGPRRSFRISLRHR

VRVVPWVKPPVARKPKDPAKPPRPSIEALAAEWAKEKAASGAPEEECVLPFLQKDAPKKL

DIVKVLGLDGFGYMDTVILSLKSYRKNIPITVGNFVIVMQSCIRLVLKKVMDAVLDMMQV

QQMEAFRRLPLPHTFQEFNIDPIKKEELDNGTEPPPYKIKIIPTGLYWLAALGLVTAQMS

ALTNHFADRKRLRSLSGILLQTQYCGWGSRALEAIEKDDFVIEFVGEVIDDETCEERLED

MRRRGDKNFYMCKVKKDFVIDATFKGNDCRFFNHSCEPNCQLQKWQVNGKTRLGVFASKA

IEVGEPLTYDYRFEQHYGPEIECFCGAQNCQGNMSTPYILNFE*

>OsSET32_SDG715

MASPPPPPRLLTPKPDPDAPLPPLPYPDPNLVQSMLFSAQSPQAQPQAPPPHIQPPASAS

AEAPSGDEKNKKKKKRARASQEMVRITNLSIADHLHYRSLVRRARLTFEALRAIYQRQDL

ATAGGIRNRFDLRASSKMLSKGLWMHRDIRTVGSIPGLLVGDSFFYRAELCVLGLHTAPQ

AGIGYIPASIVDQGHPVATSIVSSGGYLDDEDSGDVLVYSGSGGRLRNRLDHSADQTLQR

GNLALHYSCHYGIEVRVIRGHACDHSPSSKVYVYDGLYRVVTSTFGPGKSGRDVCKFKLV

RIPGQDDLGSKAWHTAAELKDALDSKIRPPKYISLDIAKGKEPFRVPLYNKLDDDRSPLF

YDYIACPDFPTTQQLLKRQTQRGCHCAELCGSRCSCERKNRGADGPVYTSDGILLRGRPL

VYECGPLCGCPMTCPNRVTQQGMKHRLEVFRSKETGWGVRTLDLIQPGAFICEYAGDVLS

LDSHSGDAPLPPMEDGSSIIDPTKFPERWREWGDASVVYPDRVPHFPLFAGARYRLDVSQ

RRNVACYISHSCSPNVFLQYVIRGNEDESYPHMMVFAMETIPPMRDLSIDYGLD*

>OsSET33_SDG723

MVIAVEGGFVHEEEEVDHPIRYLPLGRVYSSSAPCPLPKKPRSAEDGKPPVIVYYRRRRK

KPRVEGPPPSPATAPPMLHPREDDEDEEVTRRKGSLKYELLSLGQAPPALGGDGEEPARR

RCLRRSGGAERRGYFSEPKRRQRQGVHKEAASSAGRRWLELEIEAADPLAFVGLGCKVFW

PLDEDWYKGSITGYNEATKKHSVKYDDGESEDLNLADERIKFSISSEEMKCRNLKFGISN

LNKRGYDELLALAVSLHDYQGLDPGDLVWAKLTGHAMWPAVVVDESNVPANRALKPGRLD

QSILVQFFGTHDFARIKLKQAVPFLNGLLSSLHLKCKQARFYRSLEEAKEFLCTQLLPEN

MLQLQKSMEKGSSDANSNKDVHSCDNLSEDKTAESGGDYDEMTPIELGNLRVSKLGRIVT

DSDYFHNKKHIWPEGYTAFRKFRSVKDPHVVILYKMEVLRNSDIKARPLFRVTSEDGTQI

DGSTPNTCWKEIYCRLKEKQRNVASGLDRDVCQGSGSYMFGFSNPQIRQLIQELPNARSC

LKYFENAGDTFRGYRAVHVNWKDLDYCSVCDMDEEYEDNLFLQCDKCRMMVHARCYGELE

PLNGVLWLCNLCRPEAPRVSPRCCLCPVTGGAMKPTTDGRWAHLACAIWIPETCLKDVKR

MEPIDGLSRINKDRWKLLCSICGVAYGACIQCSHPTCRVAYHPLCARAADLCVELEDDDK

IHLMLLDEDEDPCIRLLSYCKKHRQPSTERPSLESNLAKPAVVVQTDAVPPSGCARTEPY

NIHGRRGQKQPQVMATASVKRLYVENMPYIVSGFCQNRVGHDAISEPIQSVGFLDVAHQE

AVGNVSSMIEKYKSMKATFRRRLAFGKSRIHGFGVFAKVSHKAGDMMIEYIGELVRPPIS

DIRERRIYNSLVGAGTYMFRIDDERVIDATRAGSIAHLINHSCEPNCYSRVISVLGDEHI

IIFAKRDINPWEELTYDYRFVSSDQRLPCYCGFPKCRGVVNDVEAEGQSAKIRVNRSELF

QQ*

>OsSET34_SDG724

MIGRYGHPPNPLLLLRPHLASFLRRLPPLRHCSLAYGLELVADPRHRRDLCLPPMPRPAK

IRKKHENVFDQLIKAIKAPVDFDLPPVLKEWKSNYYVPIKRNAYITRKRVEDDGIFCSCT

PSGSSATCDKDCQCGMLFSCCSSTCKCENKCANKPFQHRTLRKTKLIKTEKCGNGVVAEE

DIKKGEFVIEYVGEVIDDRTCEQRLWKMKRQGDTNFYLCEVSSNMVIDATNKGNMSRFIN

HSCEPNTEMQKWTVEGETRVGIFALRDIKTGEELTYDYKFVQFGADQDCHCGSSNCRKML

GITKPVNSIVLHNGNLSQDQHVRKKRKTYLENCIGEIVRLWHRRHSMYLAASIYDFNERN

GIHTLLFTDATIEEFDLREEDWDFLPDPDGPEEV*

>OsSET35_SDG727

MGGVEPPPPPAPAPAGLAAWLVARGVKRRAVSAKRSWPPLCGRFPAPPPPPPTAPVAGDD

GSKGVEGVVANQGGAEGVAVSPDRSVPLSGGPTAASSLPPPPPSIIAGNVGDNSVEGRGD

VGDKGVHGVTEGNEEEQVASAVVSSAACIGALSHGPSQPEAERMEVDEGEGRETGEAQLP

NDTDARLSDDQGMGEVLDVMPLAVAAPVSCGANVSNGSAENVRDVASLLMDREGGKWGCE

FERKEVTSDRDGRETESRVGVGQLERTNDVHDGGRKKRWLMSVLNPPPKRRAISAIRKFP

RDCGRAASTLAESGASMEELPLEATPISVATGGASMEDSLARTPISVQGASLVCGLDHSS

EAIDGKTIEDDESSKVENRIQEFQVATNVALDDFEGAKNGSTHPNDSIAKPSPSHGFVER

VNGKGSQQEKKLVARSAGDGKMVSKYEERLQKGTPETRMRDLVDVKAKKKILKSDKMNGA

LQNDARSSGDGKMKTKASSTQRGVVRSDMSLKQGDIARKVDATGKCKGGVNSLIKEATSG

KHATTNGIEENDDRDLVSDRIIVQALMAPDKCPWTRRRKSIGGSSESRTPKLKKKFGRPR

KELKDTTPREEVSPEVASCKAIKHEAIEDKEDSYFEDEGNSKASYSDVEGNSKELVRGGK

ALVVCGGKKELCVTLPPSAPFGTDPRSKIRNLLIKFHAACRKLVQVEEQHKGNIGRIDIE

AGKALKQNGFIKPGPIVGNVAGVEVGDEFNFRIELSFVGLHRPYQGGIDSTKVNGILVAI

SIVASGGYHDELSSSDELIYTGSGGKAIGNKAAGDQKLERGNLALKNSIETKTPVRVIHG

FKGHSKGEASHSKSKQISTYIYDGLYMVVDYWKEGPEGSMVYKYKLQRIPGQPELALHII

KATRKSKVREGVCVPDISQGRERIPIPAINTIDDTQPTAFKYTTEVIYPHSYAKEPLKGC

DCTNGCSDSNRCACAVKNGGEIPFNSNGAIVEAKPLVYECGPSCRCPPTCHNRVSQHGIK

IPLEIFKTGNKGWGVRSLSSISSGSFVCEYAGEVLQENGDEHVETDEYLFDIGHHYHDEV

WEDPKFEGILGLESSTSKTTEDTEGSKTTEDTEGSTIDASKCSNVGRFINHSCSPNLYAQ

NVLWDHDDMKKPHIMFFATENIPPLQELTYDYNYGKVEDKNGKEKVKPCFCGSPDCSRRL

Y*

>OsSET36_Os09g24530

MAAAIHHHHLLPPRLLSVHPQPPRLRLRRPLPRRAAASGAAAGTSSSTAAAPPPTDAALQ

EFRRWVSSHGADAGAGAAAPAAVPEGGLGLVAARDLPRGEVLAEVPKKLWLDADAVAASD

LGGAVGRGGLRPWVAVALLLLREAARGAGSPWAPYLAILPRQTDSTIFWSEEELLEIQGT

QLLSTTMGVKEYVQSEFESVEAEIISENRELFPGTVTFNDFLWAFGILRSRVFAELRGDK

LALIPFADLVNHSDDITSKESSWEIKGKGLFGRDVVFSLRTPVNVKSGEQIYIQYDLDKS

NAELALDYGFTESNSSRDAYTLTLEISESDPFYDDKLDIAELNGMGETAYFDIVLGESLP

PQMLPYLRLLCLGGTDAFLLEALFRNAVWGHLELPVSQDNEEAICQVIRNACKSALGAYH

TTIEEDEELLGSENLQPRLQIAVEVRAGEKKVLQQIDDIFKQREEELDGLEYYQERRLKD

IGLVGDNGEIIFWES*

>OsSET37_SDG732

MTTLDRTKCQQQGSRLLVRKLPKYVSLNCIVNETNSEDACSGSASIDSSLIATGITNDNR

KSPKIVPLNLILKKAKRCHAIKPLSKTENIHFSEEKSSDGSADKSSSGDRSFSPQDELWS

PKKNRYSSNVSRPHVKTDCQSPCCVLEEDEPLSLADMGTSQLSASRSRGSKNQRACISLN

RMERCEEFTNESACSPCGDKHAAVQACVTKFERYIQRPSLDASCCVCGISNLEPSNQLIE

CSKCFIKVHQACYGVLKVPRGQWFCKPCKINTQDTVCVLCGYGGGAMTRALKAQNILKSL

LRGIATAKRSDKYVYSSGNVNSECTSKLHGEYVRHDSFNGHRSRSFNAISSFGIKEASIG

SARGDIISKSWTSNRNSSLLGPRTRQWVHVVCGLWTPGTKCPNTITMSAFDISGASPAKR

NTECSMCNRTGGSFMGCRDVNCSVLFHPWCAHQRGLLQSEPEGEHNENVGFYGRCLDHAM

LDPNHVNPKKECLRSNDWTCARTEVFRGRKGDSFGANRSRKPEEKFGECSVSQEQINAWI

RINGSKSCMRGQKEYVHYKQLKGWKHLVVYKSSIHGLGLYTSEFIPRGSMVVQYVGEIVG

QCVADKREIEYQSGKRQQYKSACYFFKIGKEHIIDATRKGGIARFINHSCQPNCVAKIIS

VRNEKKVVFFAERHINPGEEITYDYHFNREDEGQRIPCFCRSRGCRRYLN*

>OsSET38_SDG741

MFHHLRRRLLCTAAAPPIRVALTESSGRGVFATRPISAGEVLHSAQPLVSHPSPPLIHEV

CYSCLRRKSGSGGGSSGSCYFCSDACREHAKGFHGVEKKADWSLFDDHCSSRGLKYPYMA

KRLACMVISGAVSADCLDILQPARLHQGTLTEMEEEFALLDSTFRKAGFQEEITTFLTKE

WYINVLARIRINAFRIELVASSYENLLSSAVASVSCDAAVGNAVYMLPSFYNHDCDPNTH

IVWLASADARLKALRNIEEGEELRICYIDASMDVDARQRILAEGFGFECRCQRCLSGD*

>OsSET39_Os10g36250

MERLKSAVPAELRRAVGEGTAADLPSTTSRLLAFLEALPLFRQVIGELTDPELALCRKDK

GRAAELKGKGNACFSKREFEQALGFYSQALRYFPISPDGTDASLIATLYVNRASTMHKLG

LLEECLRDCDRAISVSPNYAKAWYRRGMVNASFRNYSSSIHDLEVALSMEVTSSGKSNIE

QELKLILQKHQNVNEVGTASSNCINADMPHTEQQPKVILECTSTPNKGRGMSSPNDISPA

SLIHAEDPLAVIIMKSCRDTHCHYCFSEAPADVVVCPSCTIPIYCSNRCQEKAIGQMSCN

QNTHLESNNNVVDIAKLSVTSTKSKTPDSKQIAEHRHECGGACWAAVLPADIVLAGRIMA

QYIEKQLLVGKRSTISGPNLDLVHHYDQDSSASKFESHIYATVLFLCLQSYYKSGVSWAE

DSLSQLVLLICQIKVNSIAIVHMKSMDGVKALTKGFSGFSGDVMCSVEQVRVAQAIYMSG

SFFNHSCRPNIHAYFHSRTLILRSTEYIKAGSPIELSYGPQVGEMDLPERQKSLRENYYF

SCGCSSCSVLSLSDLVMNSFCCPQSNCLGAVSELIHHRHKENFVHVSIGESHVCTLSLPD

VSKFDEDIVKVGKLFFKSDTMFNIDPGFCMSCRSQLDLSSAVAMSDRATSKINRLKELPS

LDNVPEVLIAEALQSLERIEKLRHPYSKTLAQSHDTIAEAFAKVGDQEQARKHCEASIKI

LEKLYHPRHIIIAHELIKLVSIELSMGDGASAAAAFARADAIFSLYYGPDVERILPYVDV

LRRTVSERSIDSC*

>OsSET40_SDG733

MENSEDEAESDKLPLDLEPLRSLAPKFPTILGYDVETQSTDPLLVYATPSIPCSSSEQPQ

EAPASFSLPLPKSPVPIKATPISAAFPTPQHEDESSDQDYKPFCKNKKPAMPKRAKRPQQ

AEKSNDANIKRRSIRRNLDNEFNLCSSSSDNPKESVEGILMMFDSLRRRVLQLDEKEDAS

RRADLKAGTLMMQNNLRINNHKMIGHVPGVEVGDIFFFRIEMCIVGLHAPAMGGIDYISS

KNKDETLAVCIISSGGYENDDDDTDILVYTGQGGNSRHKEKHDQKLERGNLALMNSKSKK

NQIRVVRSAQDPFCNSGKIYIYDGLYRIEDTWTDTAKNGFNVFKYKLRRDPGQPDGISLW

KMTEKWKANPATREKAILLDLSSKVEHLPVCLVNDVDDEKGPSHFNYVAGVKYLRPLRKT

KPLQCCKCPSVCLPGDPNCSCAQQNGGDLPYSATGLLAKHTPMVYECSSNCQCSHNCRNR

ITQKGIKLNFEVFWTGDRGWGLRSWDPIRAGTFICEYAGEVIDETKMDIDVEEDKYTFRA

SCPGNKALSWNLGEELLEEKSTAVITKNFKKLPIIIRANNEGNVARFLNHSCSPNLLWQA

VQYDHGDDSYPHIMFFAMEHIPPMTELTYDYGTRGAPPGFEGKPFKACKLKSCLCGSKHC

RGLF*

>OsSET41_SDG704

MAGTRQTTSVPMDNAAVVDAKPLRTLTPMFPAALGLHTFTAKENSSSIVCITPFGPYAGG

TEQAMPASIPPMFASPAAPADPNQRQPYAVHLNGAAPANGTANNTGVIPDLQIAVAGTVE

SAKRKRGRPKRVQDSSVPSAHLVPSAPGGNITAVQTPPSATTDESGKKKRGRPKRVQDVP

VLSTPSAPQVDSTVFQTPASAVNESVTRKRGRPRRVQDGADTSAPPIQSKYNEPVLQTPS

AVTLPEDGKRKRGRPKRVPDGALIPLSHSGVSIDDDSGEIITGKRGRPRKIDVNLLNLPS

LFSDDPRESVDNVLMMFDALRRRLMQLDEVKQGAKQQHNLKAGSIMMSAELRANKNKRIG

EVPGVEVGDMFYFRIEMCLVGLNSQSMSGIDYMSAKFGNEEDPVAISIVSAGVYENTEDD

PDVLVYTGQGMSGKDDQKLERGNLALERSLHRGNQIRVVRSVRDLTCPTGKIYIYDGLYK

IREAWVEKGKTGFNVFKHKLLREPGQPDGIAVWKKTEKWRENPSSRDHVILRDISYGAES

KPVCLVNEVDDEKGPSHFNYTTKLNYRNSLSSMRKMQGCNCASVCLPGDNNCSCTHRNAG

DLPYSASGILVSRMPMLYECNDSCTCSHNCRNRVVQKGSQIHFEVFKTGDRGWGLRSWDP

IRAGTFICEYAGEVIDRNSIIGEDDYIFETPSSEQNLRWNYAPELLGEPSLSDSSETPKQ

LPIIISAKRTGNIARFMNHSCSPNVFWQPVLYDHGDEGYPHIAFFAIKHIPPMTELTYDY

GQSQGNVQLGINSGCRKSKNCLCWSRKCRGSFG*

>OsSET42_SDG734

MASSASVSPAAASHHRLLLPCSPRRLPRPRPRPSPRLLRSARPRLVACHADTLLPSSSPA

AAAAACASTASANGFSDWLREHGLPPGKVAILDRPVPCFREGKDLPLHYVAAGQDLEAGD

VAFEVPMSLVVTLERVLGDESVAELLTTNKLSELACLALYLMYEKKQGQDSFWYPYIKEL

DRQRGRGQLAVESPLLWTESELNYLKGSPIKDEVVARDEGIRREYNELDTLWFMAGSLFQ

QYPFDIPTEAFPFEIFKQAFVAVQSCVVHLQKVSLARRFALVPLGPPLLTYKSNCKAMLT

AVGDSVRLVVDRPYKAGEPIIVWCGPQPNSRLLLNYGFIDEDNPYDRIVIEASLNIEDPQ

FQEKRMVAQRNGKLAIQNFHVCVGKEKETIAEMLPYLRLGYISDPDEMQSILSSEGDTCP

VSPCTERAVLDQLVGYLESRLADYPTTLDEDDAMLADGNLEPKKEVATRLVRLEKKLLHG

CLQAANEFINDLPDHTVSPCPAPFAPELK*

>OsSET43_SDG717

MPHSRSDSGSGSRGADPCRGRKRGRLLMLEEEEEEEESGMEGCSAPACGDVRGDFVGWCS

DRHQVASCSGDQTQSASMFAAMQENACSIDSKGVVCPQSGLGYSAGQNGTHGGGGSMLHQ

NLEGCMYMNQLGQMCGPYPPEQLYDGLSTGFLHRDLAIYAVFGGKMANPVSLGSLKQFLS

QWSSDSVVATRDESVENKKMAPVNKLILPDNLSSEESCWMFEDAEGRRHGPHSLAELSYW

HHSSYLHDLSMIYHVDSKFGPFTLVSLIDWWSGGTEHSESSANDSGSLNALMDDVVEDIS

HQLHAGIMKSARKVFIDEIFSSVLPEMIACRKTEKQMAAKRKSQAAKTDNVSNKNALVLK

GKGDGTSTRPKSLNSYNNKVPEDPSVAVQSTAMQYEFADILSAVWETIYNKSMKSIWDEV

LYDPVMDYCDAWLKRKNESNLLSTVVPGASDNQKMQDTDEMSPKAICDSDAPESDMDFPP

GFGPNQESAEHSHSACVEYVTEKTDGRSGSSITLFSGPLGRVQVMLANELYVAAKEALFQ

HFEEVISEEITNCLCIGFEDDINQERIRTPVHAPEPSSPPGISVHETPSPAEMPRDEISD

MAEMARDEISDMAVDTIPCPADMAASGTSTVPEVTTDKLIIPYVEHQSPSASHASIFEKL

DAHEEAELDDSFDEVPPGTEAGLASLVIMEKNKYQPSKSVDSVLDIYRYTSWAFFRQILH

ESVMKEWASLFSGALSNCFDSWYARKNIVAKTMDDTLRPKEYTYYRKRKLRKNCEASSSE

KPMDEQLSRPLRDLVECKVNMKNIHRSSKAGISQRVSVVEKPSKKRAKPSHNDNINLNIQ

QDLKLLSDKVPKRNRSSHPTSKPLVSSKVPTEDRTTSAMPAKKRKQKNLATESNLKTKAV

ILSPESHGCEAPTEKRTTAIMPVNKRKKNLSGESKLKAKPLTSPESYVCEAPIDNRTTST

MPAKKRKQKNLSNESNLKKKPLVLCPESYGCARASVSGWEWRDWARNATPSERAQVRGYR

VRSILSAPENNVLKSSQVKGSSARTNRVKLRNLLAAAEGTDLLKIMQSKSRKKRLRFQRS

KIHEWGLVALESIDAEDFVIEYVGELIRRQVSDIREDQYEKSGIGSSYLFRLDDDYVVDA

TKRGGLARFINHSCDPNCYTKVITVEGQKKIVIYAKRRIYAGEELTYNYKFPLEEKKIPC

HCGSQRCRGSMN*
