# Supplementary material for: Genome-wide identification and expression profiling of SET DOMAIN GROUP family in Dendrobium catenatum
Source: BMC Plant Biol. 2020 Jan 28;20:40. doi: 10.1186/s12870-020-2244-6 (PMC6986063; doi:10.1186/s12870-020-2244-6)
Supplement: Supplementary file 2 — Additional file 2. Identification and classification of SDG genes in P. equestris. Sequences and information of P. equestris SDG genes and proteins came from NCBI database (https://www.ncbi.nlm.nih.gov/). [file 12870_2020_2244_MOESM2_ESM.docx]

| **Table S2**  Classification of SDG genes in *P. equestris*. | | | | | | |
| --- | --- | --- | --- | --- | --- | --- |
| **No.** | **Name** | **Gene ID** | **Clade** | **exon number** | **Length (aa)** | **Isoform** |
| 1 | PeCLF | LOC110023511 | I | 16 | 913 | 0 |
| 2 | PeSWN | LOC110031838 | I | 18 | 826 | 0 |
| 3 | PeASHH1 | LOC110026917 | II | 10 | 498 | 0 |
| 4 | PeASHH2 | LOC110020099 | II | 18 | 1,934 | 2 |
| 5 | PeASHH3 | LOC110034120 | II | 13 | 373 | 0 |
| 6 | PeASHR3 | LOC110023344 | II | 11 | 485 | 1 |
| 7 | PeATX1 | LOC110032450 | III | 24 | 1,076 | 0 |
| 8 | PeATX3a | LOC110018473 | III | 22 | 877 | 0 |
| 9 | PeATX3b | LOC110022600 | III | 23 | 1,068 | 0 |
| 10 | PeATX3c | LOC110022283 | III | 25 | 937 | 3 |
| 11 | PeATX3d | LOC110023995 | III | 20 | 2,048 | 3 |
| 12 | PeATXR3 | LOC110032823 | M | 21 | 2,350 | 0 |
| 13 | PeATXR7 | LOC110025899 | M | 21 | 1,308 | 0 |
| 14 | PeATXR5 | LOC110036029 | IV | 4 | 241 | 0 |
| 15 | PeATXR6 | LOC110025867 | IV | 5 | 342 | 0 |
| 16 | PeSUVH1a | LOC110024649 | V | 8 | 702 | 5 |
| 17 | PeSUVH1b | LOC110027660 | V | 3 | 677 | 0 |
| 18 | PeSUVH2a | LOC110021228 | V | 4 | 625 | 2 |
| 19 | PeSUVH2b | LOC110039425 | V | 2 | 652 | 0 |
| 20 | PeSUVH4 | LOC110031562 | V | 7 | 271 | 0 |
| 21 | PeSUVH45 | LOC110023823 | V | 3 | 778 | 2 |
| 22 | PeSUVH5a | LOC110022007 | V | 5 | 1,034 | 4 |
| 23 | PeSUVH5b | LOC110038667 | V | 1 | 1,101 | 0 |
| 24 | PeSUVR14a | LOC110037500 | V | 10 | 756 | 2 |
| 25 | PeSUVR14b | LOC110033859 | V | 12 | 722 | 6 |
| 26 | PeSUVR3 | LOC110034752 | V | 1 | 230 | 0 |
| 27 | PeSUVR4 | LOC110035853 | V | 3 | 177 | 0 |
| 28 | PeSUVR5 | LOC110031970 | V | 14 | 1,435 | 2 |
| 29 | PeASHR1 | LOC110029940 | VI | 12 | 338 | 0 |
| 30 | PeASHR2 | LOC110037878 | VI | 2 | 384 | 0 |
| 31 | PeATXR1 | LOC110022208 | VI | 1 | 521 | 0 |
| 32 | PeATXR2 | LOC110018090 | VI | 20 | 511 | 6 |
| 33 | PeATXR4 | LOC110027897 | VI | 9 | 318 | 1 |
| 34 | PeSDG42 | LOC110038127 | VI | 16 | 763 | 0 |
| 35 | PeSDG43 | LOC110019799 | VII | 13 | 482 | 0 |
| 36 | PeSDG45 | LOC110025947 | VII | 8 | 496 | 1 |
| 37 | PeSDG46 | LOC110027377 | VII | 5 | 493 | 1 |
| 38 | PeSDG47 | LOC110026574 | VII | 13 | 555 | 2 |
| 39 | PeSDG48 | LOC110027008 | VII | 12 | 414 | 1 |
| 40 | PeSDG49 | LOC110033101 | VII | 15 | 565 | 0 |
| 41 | PeSDG50 | LOC110021556 | VII | 9 | 482 | 3 |
| 42 | PeSDG51 | LOC110030312 | VII | 14 | 512 | 2 |
