# Supplementary figures and images for: Genome-wide identification and expression profiling of SET DOMAIN GROUP family in Dendrobium catenatum
Source: BMC Plant Biol. 2020 Jan 28;20:40. doi: 10.1186/s12870-020-2244-6 (PMC6986063; doi:10.1186/s12870-020-2244-6)

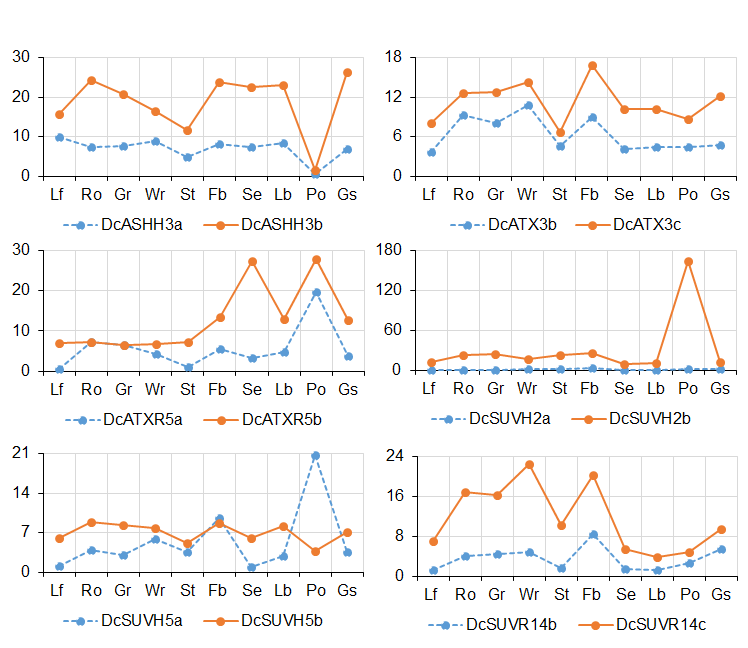

Supplement: Supplementary file 4 — Additional file 4. Expression patterns of duplicated SDG gene pairs. The FPKM values of the duplicated DcSDG genes in different tissues and organs was used for comparison. Lf: leaf, Ro: root, Gr: green root tip, Wr: white part of root, St: stem, Fb: flower bud, Se: sepal, Lb: labellum, Po: pollinia, and Gs, gynostemium. [file 12870_2020_2244_MOESM4_ESM.tif]

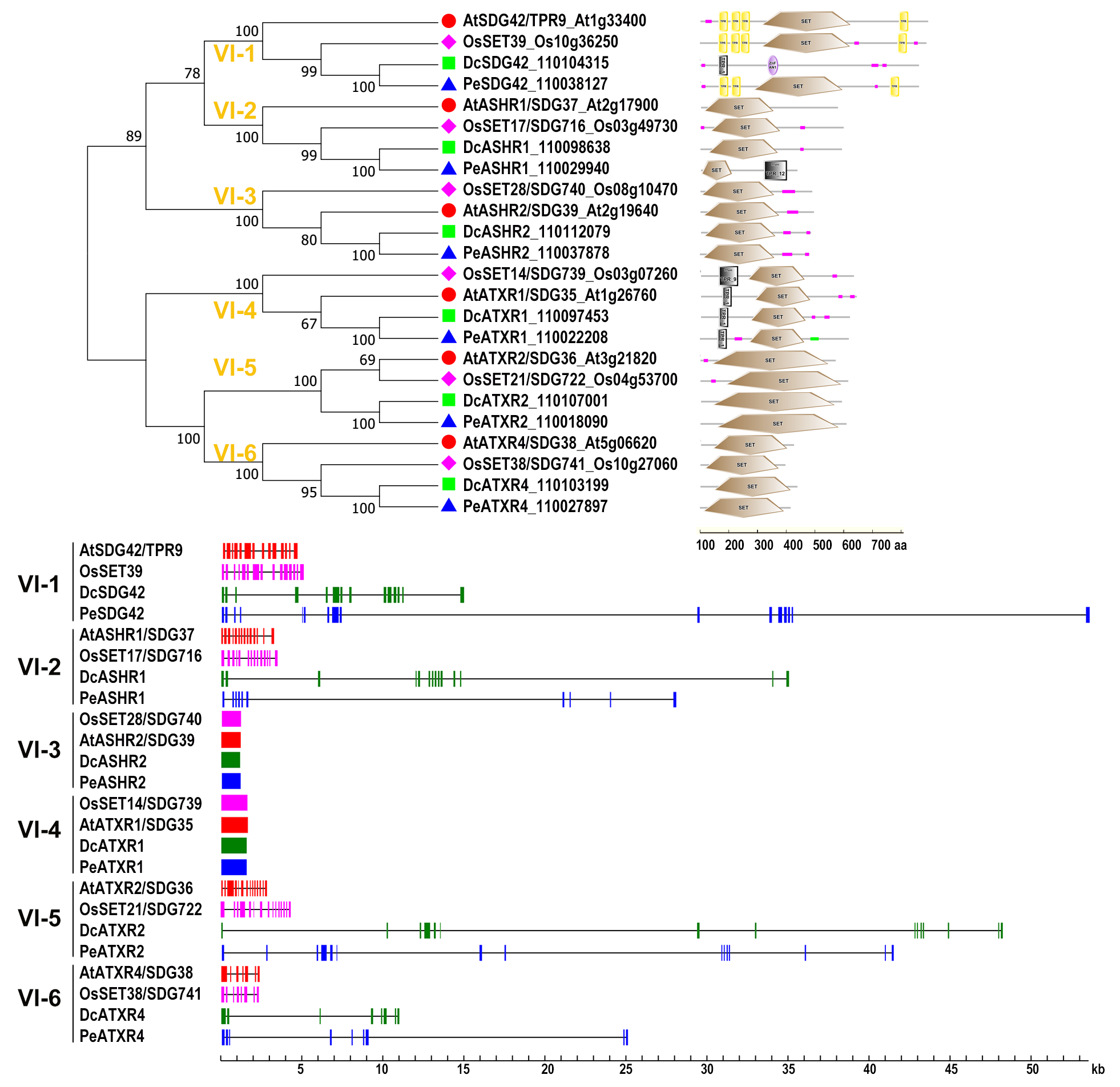

Supplement: Supplementary file 7 — Additional file 7. Domain organization and gene structure of the class-VI DcSDGs. The NJ tree was generated using MEGA7 with parameter settings as Fig. 1 based on full-length amino acid sequences of Class-VI SDGs in D. catenatum, P. equestris, Arabidopsis and rice. The number along the tree branch indicates bootstrap value. Different conserved protein domains are colored as indicated. Gene structures of SDGs in each species were indicated in distinct colors. The solid boxes represent exons and black lines represent introns. [file 12870_2020_2244_MOESM7_ESM.tif]

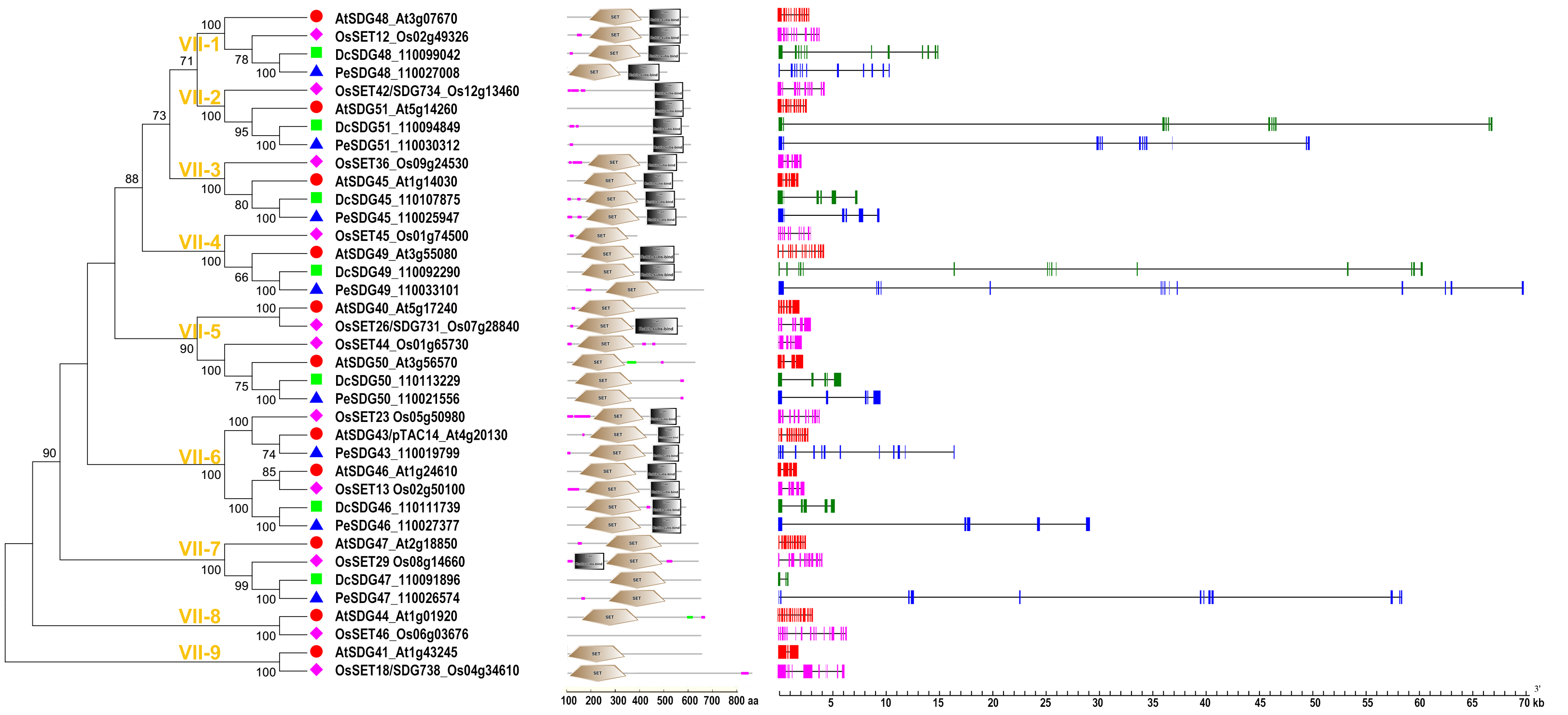

Supplement: Supplementary file 8 — Additional file 8. Domain organization and gene structure of the class-VII DcSDGs. The NJ tree was generated using MEGA7 with parameter settings as Fig. 1 based on full-length amino acid sequences of Class-VII SDGs in D. catenatum, P. equestris, Arabidopsis and rice. The number along the tree branch indicates bootstrap value. Different conserved protein domains are colored as indicated. Gene structures of SDGs in each species were indicated in distinct colors. The solid boxes represent exons and black lines represent introns. [file 12870_2020_2244_MOESM8_ESM.tif]
